# Synthesis, Structure, Thermal Behavior and *cis*/*trans* Isomerization of 2,2′-(EMe_3_)_2_ (E = C, Si, Ge, Sn) Substituted Azobenzenes

**DOI:** 10.3390/molecules24020303

**Published:** 2019-01-15

**Authors:** Jonas Hoffmann, Thomas Josef Kuczmera, Enno Lork, Anne Staubitz

**Affiliations:** 1 Institute for Analytical and Organic Chemistry, University of Bremen, Leobener Straße 7, D-28359 Bremen, Germany; jonas.hoffmann@uni-bremen.de (J.H.); kuczmera@uni-bremen.de (T.J.K.); 2 MAPEX Center for Materials and Processes, University of Bremen, Bibliothekstraße 1, D-28359 Bremen, Germany; 3 Otto-Diels-Institute for Organic Chemistry, University of Kiel, Otto-Hahn-Platz 4, D-24098 Kiel, Germany; 4 Université Rennes, CNRS, ISCR-UMR 6226, 263 Av. du Général Leclerc, F-35042 Rennes, France; 5 Institute for Inorganic Chemistry and Crystallography, University of Bremen, Leobener Straße 7, 28359 D-Bremen, Germany; enno.lork@uni-bremen.de

**Keywords:** azobenzene, tetrels, group 14 elements, transmetalation, molecular switches, cross-coupling, photo responsiveness

## Abstract

The synthesis of a series of 2,2′-bis(trimethyltetrel) azobenzenes is reported, evaluating the different synthetic approaches that different group 14 element substituents individually require. The synthetic access to the carbon substituted congener is very different from the heavier tetrels, in that the key step is the formation of the N=N bond in azobenzene, rather than the azobenzene-C bond. Sn could be introduced with a cross-coupling route, whereas the Si and Ge congeners were prepared by a stannylation-lithiation-electrophilic quenching sequence. Iodo-lithium exchange was also a possible route to obtain the dilithiated species, which can be attributed to the chelating effect of the nitrogen atoms. However, the organo-lead species could not be obtained via these routes. The resulting structures were fully characterized (NMR, FTIR, HRMS and XRD). Furthermore, their thermal properties (TGA and DSC) and their photoswitching behavior in solution (UV-VIS & NMR experiments) were investigated and compared for the different tetrels (C, Si, Ge, Sn).

## 1. Introduction

In main group chemistry, trends within a group play an important role for the understanding of how particular properties of a molecule can be tuned. By selecting a congener, in which an atom is replaced by another element from the same group, the geometric shape of the molecules can be changed. For instance, in organic compounds, carbon atoms can be replaced by heavier tetrels (Si, Ge, Sn and Pb) to give semi-inorganic hydrocarbons [1]. However, size is not the only change; optophysical properties can also be influenced substantially. For example, it has been shown that for cyclopentadiene analogs, in which the sp^3^ substituted C atom is replaced by a heavier group 14 element, the HOMO-LUMO gap (HOMO = highest occupied molecular orbital; LUMO = lowest unoccupied molecular orbital) is reduced due to σ*-π* conjugation [2,3,4,5,6]. Due to our interest in both main group chemistry and photoswitchable molecules, we systematically investigated the influence that could be exerted by tetrels in the *ortho*-position of azobenzenes, as well as the structural influences this substitution might trigger: Azobenzenes are thermally and photochemically stable molecular switches that can undergo a reversible *trans*–*cis* isomerization under illumination. The properties of azobenzenes, such as absorption maxima and switching behavior, can be tuned by their substitution pattern [7,8,9,10,11]. *Ortho*-substituted azobenzenes distinguish themselves from most molecular switches by their significantly longer thermal relaxation times of the *cis*-isomer back to their *trans*-isomers [12,13]. This property makes *ortho*-substituted azobenzenes suitable for several applications where long half-life times of the thermal relaxation are required, giving rise to new materials and dyes, e.g., external stimuli responsive polymers [14,15].

**Scheme 1:**
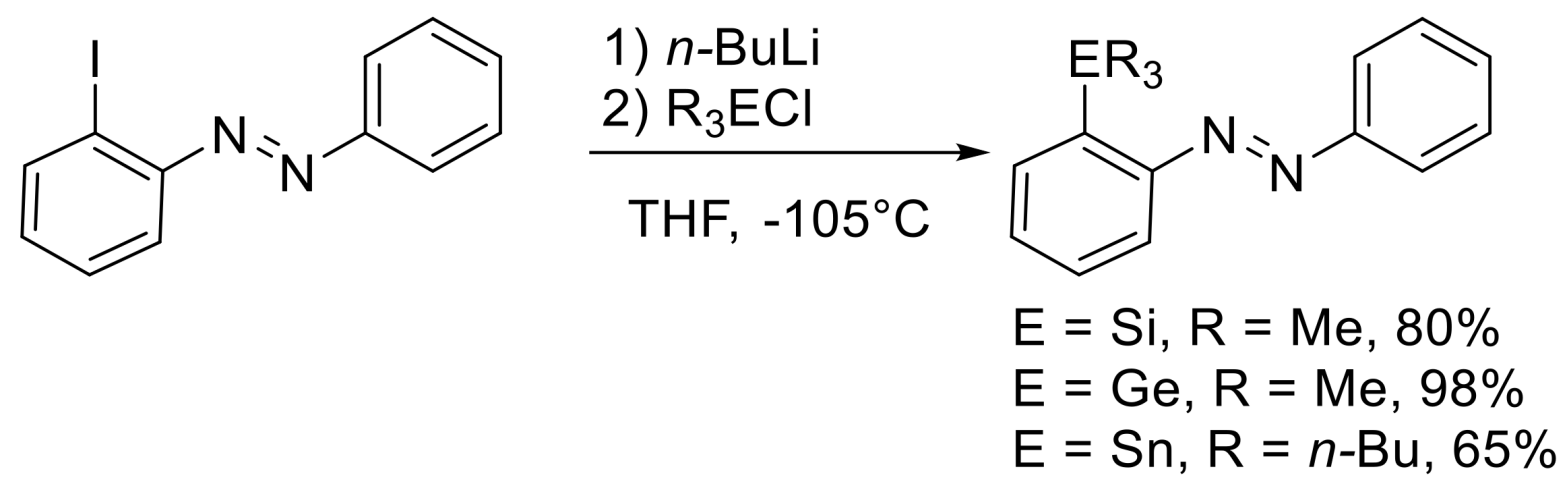
Functionalization of monosubstituted azobenzenes with organotetrels [21].

In general, *ortho*-azobenzenes can be accessed either by using C–H activation methods with late transition metals like nickel, palladium, platinum or ruthenium [16,17], by deprotonation with metal bases [18,19], or by direct halogen-lithium exchange [19,20]. The latter has been shown to proceed with high efficiency in the synthesis of several *ortho*-substituted organometallic azobenzenes (Scheme 1) [21].

This methodology is superior to the classical azocoupling approach and could be used with a high variety of electrophiles. It should also be mentioned that in this study, the *ortho*-bis(silyl) azobenzene (compound **6** in our study) was synthesized by oxidation of the respective amine. This product was used for mono-desilylation to give the monosilylated azobenzene displayed above [21]. Other metalation methods were used to introduce pentacoordinated silyl atoms in azobenzene motifs [20,22]. It was also found that substitution with heavier group 14 elements, which convey a positive inductive effect on the aromatic ring, resulted in a slight bathochromic shift of the absorption maxima compared to unsubstituted azobenzene.

Since we developed a method for the lithiation of azobenzenes by halogen-lithium exchange reactions for *para*- and *meta*-substituted azobenzenes [23,24], we were interested to transfer this methodology towards disubstituted *ortho*-azobenzenes. This process involved the synthesis of tin-substituted azobenzenes through a Kelly-Stille cross-coupling reaction, followed by a low temperature tin-lithium exchange. Functionalization could be achieved by quenching the lithiated species with a variety of electrophiles at low temperatures. Initial results showed that the structure-reactivity relationship of the *meta*-, *para*- and *ortho*-azobenzenes in the Kelly-Stille cross-coupling reaction led to significant differences in yields (*para*: 93%, *meta*: 88% and *ortho*: 43%). An advantage of a functionalization methodology that involves stannylated groups in the reagents is the good shelf-life and the potential use of these reagents as nucleophilic components in Stille type cross-coupling reactions. This was already reported for *para*-azobenzenes and gives access to substitutions with aromatic or vinylic groups [25].

In this work, we were interested in the detail of the structure/property relationship of different tetrel-substituted azobenzenes. In contrast to previous studies, the carbon congener is included and both *ortho*-positions of the azobenzene are substituted.

## 2. Results

**Scheme 2:**
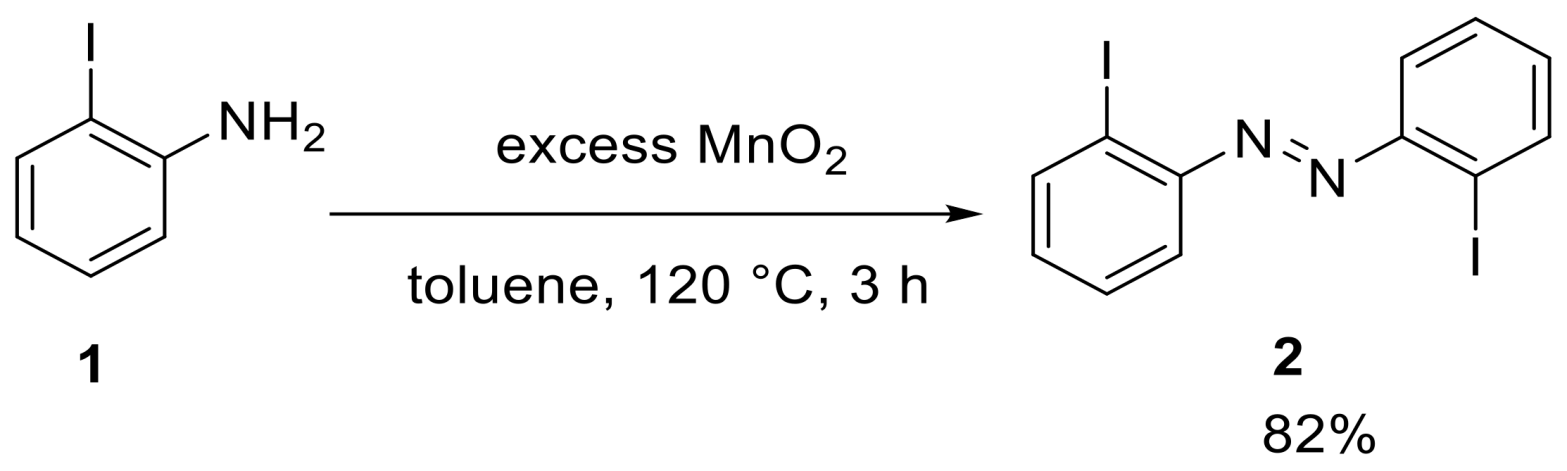
Oxidative azocoupling to give the difunctional azobenzene **2**, which can be used as the electrophilic component in cross-coupling reactions.

The investigation started with the synthesis of *ortho*-substituted azobenzenes, in which the substituent was always EMe_3_ with E = C, Si, Ge and Sn (E = Pb proved inaccessible). Therefore, we prepared a 2,2′-substituted difunctional azobenzene through the oxidation of 2-iodoaniline (**1**) with manganese dioxide to give 2,2′-diiodoazobenzene (**2**) in a yield of 82% (Scheme 2) [26].

It needs to be pointed out that azobenzenes with di-*ortho*-substituents are more difficult to prepare than those with the same substituents in *meta*- and *para*-position. Oxidizing reagents, like copper(I) bromide/air [25], copper(II)/silver(I)/tetra-*n*-butylammonium bromide [27] or diacetoxyiodobenzene [28], were explored in the synthesis, but in all cases no conversion was observed. 

**Scheme 3:**
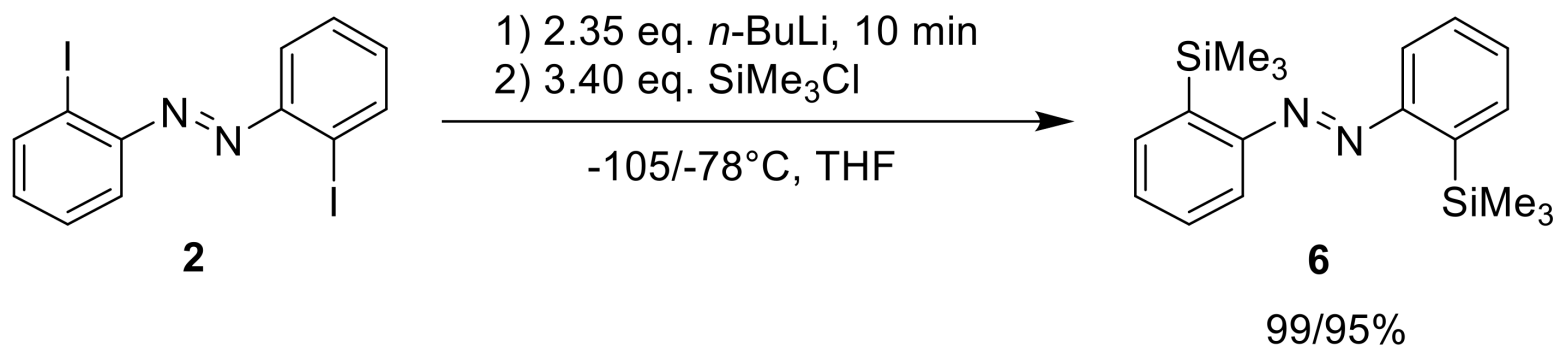
Direct metalation of azobenzene **2** to give the trimethylsilyl-substituted azobenzene **6**. (Experimental details can be found in the SI).

Following the iodo-lithium exchange, which was already reported [20], 2,2′-diiodo-azobenzene (**2**) was treated with *n*-butyllithium at a low temperature (−105 or −78°C) and quenched with a trimethylsilyl chloride electrophile (Scheme 3). The reactions proceeded very cleanly, which in our experience is different to *meta*- and *para*-iodinated azobenzenes, where the electrophilic N=N group can be attacked by the nucleophilic *n*-butyllithium. This suggests a significant stabilization of the *ortho*-lithiated species by an N→Li coordination.

**Scheme 4:**
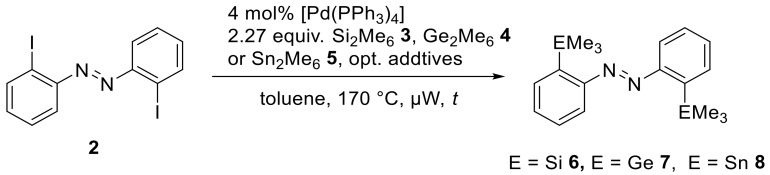
Different hexamethyl dimetals were used in a Stille-Kelly type cross-coupling protocol.

Although this is a very effective route to synthesize these *ortho*-substituted azobenzenes, we were interested in obtaining the tetrel-substituted compounds under cross-coupling conditions. Therefore, the next step to generate the EMe_3_ *ortho*-substituted azobenzenes was a palladium catalyzed cross-coupling procedure with Me_3_E-EMe_3_ reagents. Since this reaction has been already reported for *meta*- and *para*-substituted azobenzenes for E = Sn, we adapted to the reaction conditions also for other tetrels, for which this type of reagent was available (Si, Ge and Sn) (Scheme 4, Table 1).

**Scheme 5:**
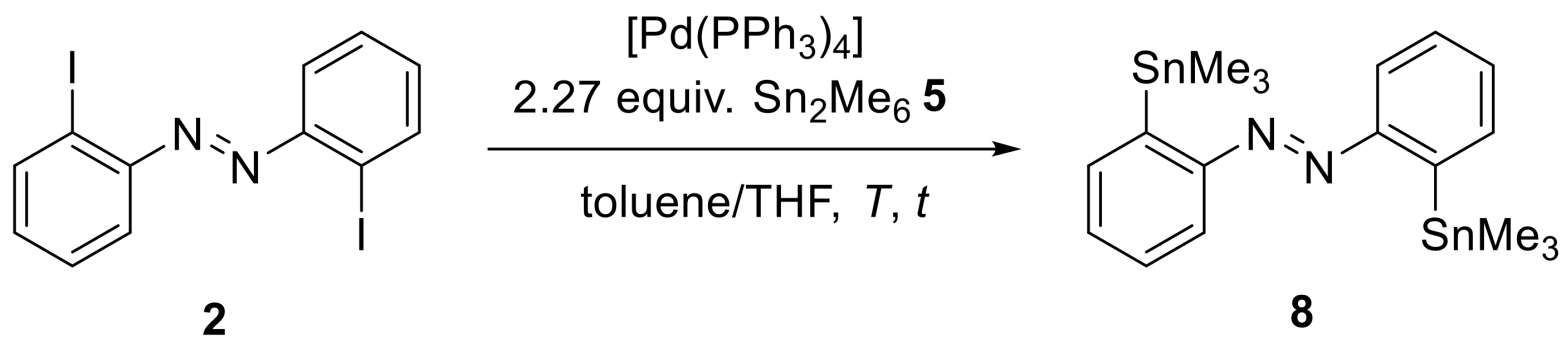
Stille-Kelly type coupling of azobenzene **8**.

We initially assumed that the metal-metal bond dissociation energy (BDE) might have a substantial impact on the reactivity towards the oxidative addition of the catalyst. The bond dissociation energies for hexamethyl dielement species have been reported: BDE (Me_3_C-CMe_3_) = 76.0 kcal/mol [33], BDE (Me_3_Si-SiMe_3_) = 69.1 kcal/mol [34] and 79.7 kcal/mol [35], BDE (Me_3_Ge-GeMe_3_) = 69.1 kcal/mol [34], BDE (Me_3_Sn-SnMe_3_) = 61.6 kcal/mol and BDE (Me_3_Pb-PbMe_3_) = 54.6 kcal/mol [34]. Since the BDE of the tin dimer is relatively lower than the other hexamethyl dielement compounds, we assumed that a more labile tin-tin bond would react more easily, but it was surprising that neither the Me_3_Si-SiMe_3_ (**3**) reagent, nor the Me_3_Ge-GeMe_3_ (**4**) reagent provided any conversion. Steric reasons may be a potential issue: The C–Sn bond is longer and thus may lead to less congestion in the *ortho*-positions. Thus, the Stille-Kelly type cross-coupling reaction with the hexamethylditin (**5**) was optimized towards the reaction temperature, reaction time and catalyst load (Scheme 5, Table 2): We envisaged that with this reagent in hand, alternative routes to the other *ortho*-substituted azobenzenes may be also accessible via tin-lithium exchange and electrophilic quenching processes (see below).

It was found that increasing the reaction temperature from 130 °C to 170 °C, along with changing from conventional to microwave assisted heating (entries 2 and 4), led to an increase in yield and a decrease in reaction time. Furthermore, the catalyst to hexamethyldistannane (**5**) ratio had a crucial impact on the success of the reaction. Increasing only the catalyst load led to a decrease in the yield (60% to 14%, entries 6 and 8). Additives like copper (I) halides [36] and lithium chloride [37] also lowered the yield of the product (entry 7). Interestingly, a high catalyst load in combination with a high amount of hexymethyldistannane (**5**) resulted in nearly quantitative yield. Combined with the fact that the reaction vessels always contained an insoluble black precipitate after the reaction, we assumed that the autocatalytic agglomeration of the palladium catalyst that can occur more easily with higher catalyst loadings may be the cause of this observation [38]. With an excess of hexamethyldistannane (**5**), agglomeration was not observed (see the high difference in yield for entries 6, 8 and 9), presumably because the desired reaction was accelerated compared to the agglomeration process. Since hexamethyldistannane (**5**) is an expensive and toxic compound, a high excess and high amount of organotin waste is not desirable. Therefore, we performed further reactions with only 2.27 equivalents of this organotin compound, accepting the somewhat lower yield. We also tested whether the stannylated product **8** might react with the iodinated starting material **2** in a standard Stille reaction, but this was not the case, which excludes this reaction as a cause for the lower yields from entries 7 to 8 (see SI).

**Scheme 6:**
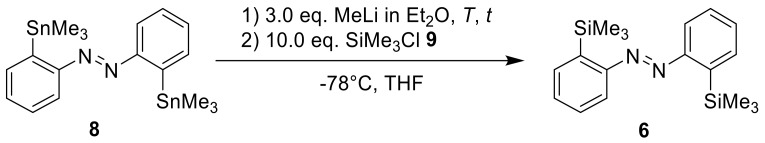
Trimethylsilyl chloride **9** was used as a test electrophile to obtain optimized reaction parameters.

To access the other *ortho*-substituted azobenzenes for EMe_3_ (E = C, Si, Ge), we followed a transmetalation procedure which was already reported for di-stannylated *meta*- and *para*-azobenzenes [23]. Initially, the metalation temperature and time were optimized, with trimethylsilyl chloride (**9**) being used as an electrophile (Scheme 6, Table 3).

**Scheme 7:**
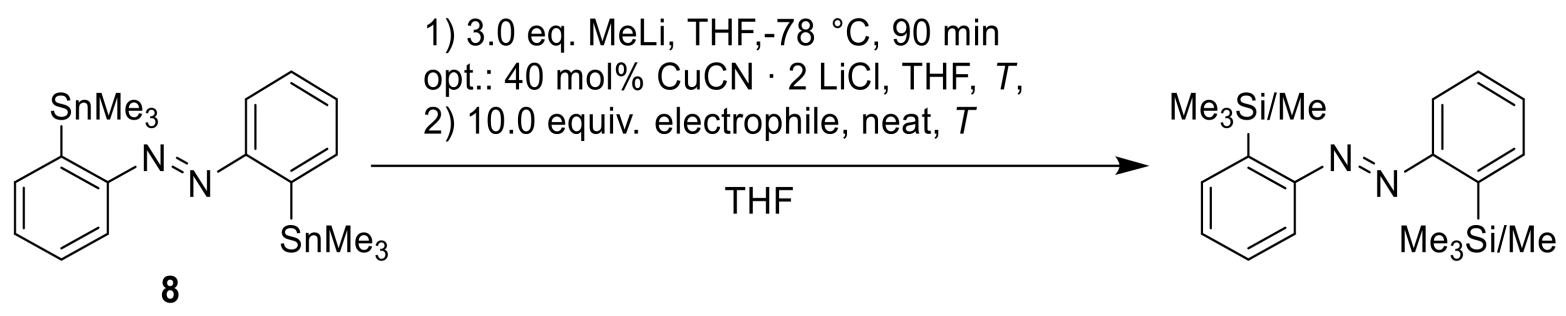
In addition to the tin-lithium exchange, we investigated the subsequent transmetalation to organocuprates and further quenching with electrophiles.

It was found that increasing the temperature for the lithiation reaction led to a decrease in product and an emergence of unassignable signals in the ^1^H NMR spectra. However, the metalation time played only a minor role in the formation of the product (Entries 6–10). The best conditions were found to when the lithiation temperature was −78 °C and a metalation time of 90 min was used. To expand the variety of electrophiles to less hard organometals, we made use of a transmetalation from the hard, reactive lithium towards the softer and less reactive cuprate, which was initially reported by Knochel [39]. This methodology has so far not been applied to azobenzene chemistry (Scheme 7).

We tested this reaction sequence with several electrophiles and different metalation temperatures (Table 4).

The yield decreased if the temperature for the lithiated species was changed from −78 °C to −40 °C (Entries 1 and 2), which may be an indication of a reductive attack by the organolithium reagent on the azo group [18]. The transmetalation to copper at −78 °C provided largely the same yields (Entry 3), although at −40 °C, using the cuprate gave a very low yield of 7% (Entry 4). For Me_3_SiI, a decrease in yield after the transmetalation to copper was observed (Entries 5–7), which was unexpected as this electrophile is softer. With methyl iodide as an electrophile, we could observe an increase in the yield by increasing the transmetalation temperature from −78°C to −40 °C (Entries 8 and 9). In that case, using the cuprate was essential for the reaction, because the lithiated species did not react with the electrophiles (Entry 10).

**Scheme 8:**
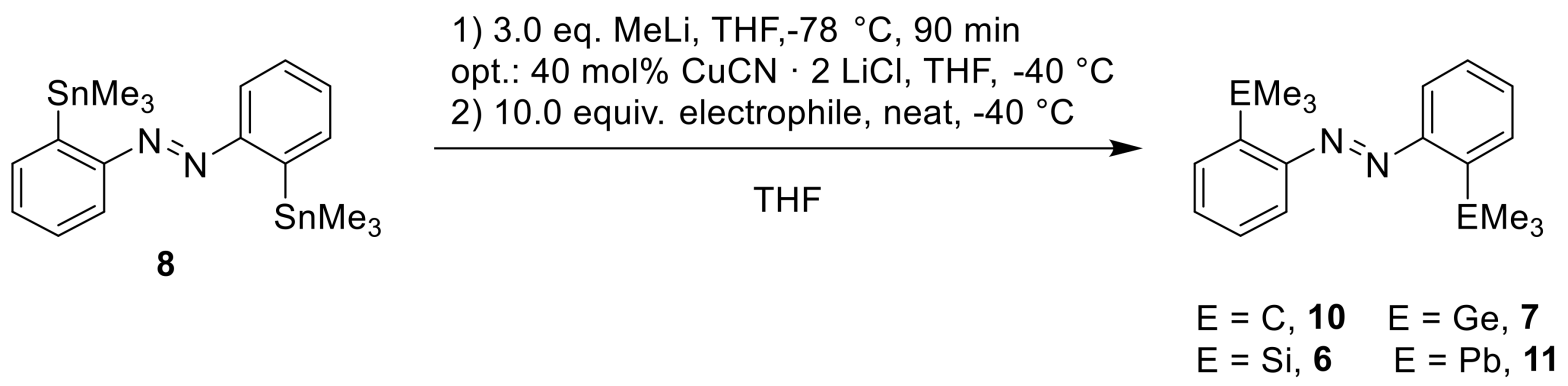
Metalation to obtain di-metalated azobenzenes **10**, **11** and **6**, **7**.

With both methods in hand, we incorporated new trimethyltetrel halides. In dependence of the nature of the respective electrophile, we were interested in the reactivity towards the hard organolithium or the soft organocuprate (Scheme 8, Table 5).

Besides the above mentioned trimethylsilyl chloride (**9**), the reaction of trimethylgermanyl chloride (**12**) to give the digermanylated azobenzene (**7**) could be performed in a good yield of 60% (Entry 3). With *tert*-butyl chloride (**13**) and trimethyllead bromide (**14**), neither reaction was successful, irrespective of whether the lithium or copper species (Entries 1 and 4) were used, but for different reasons: The nucleophilic substitution of *tert*-butyl chloride (**13**) would be highly disfavored for an S_N_2 reaction due to its steric hindrance, and also because a S_N_1 reaction is unlikely under these nonpolar reaction conditions. The reaction with trimethyllead bromide (**14**) was unsuccessful with both the lithium or copper nucleophile. It is possible that the trimethyllead bromide did react, but that the lead(IV)-substituted azobenzene was then too reactive to be isolated; recent examples of this are given in Reference [41]. This hypothesis is supported by the fact that after the reactions, a shiny insoluble black precipitate could be observed, which are probably decomposition products from lead.

**Scheme 9:**
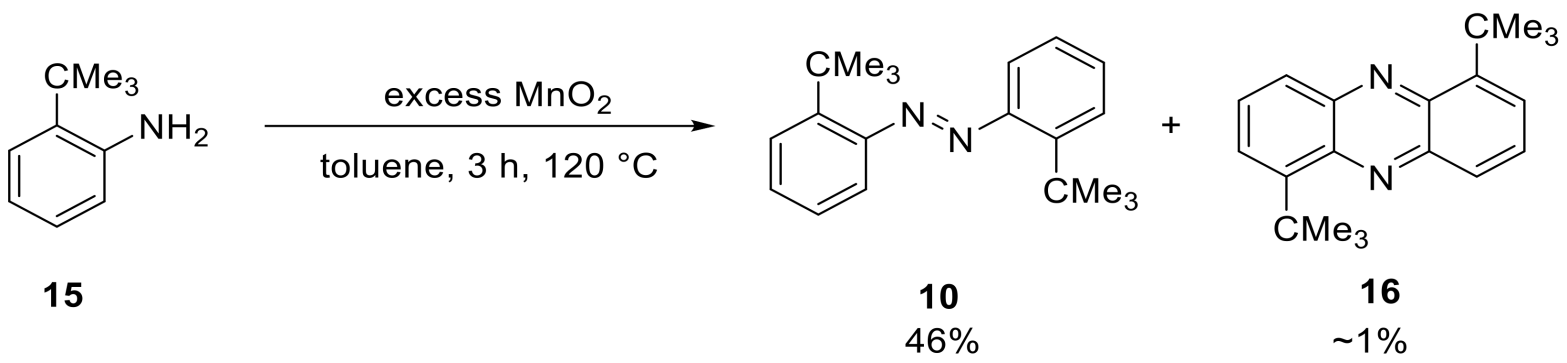
Oxidative azocoupling of aniline **15** with manganese dioxide as an oxidant. The product **10** and by-product **16** could be separated by column chromatography.

Since we were interested in comparing the influence of different trimethyltetrel substituents in *ortho*-position of the azobenzenes, we obtained the carbon-derivative via oxidation of the respective aniline (Scheme 9).

**Figure 1:**
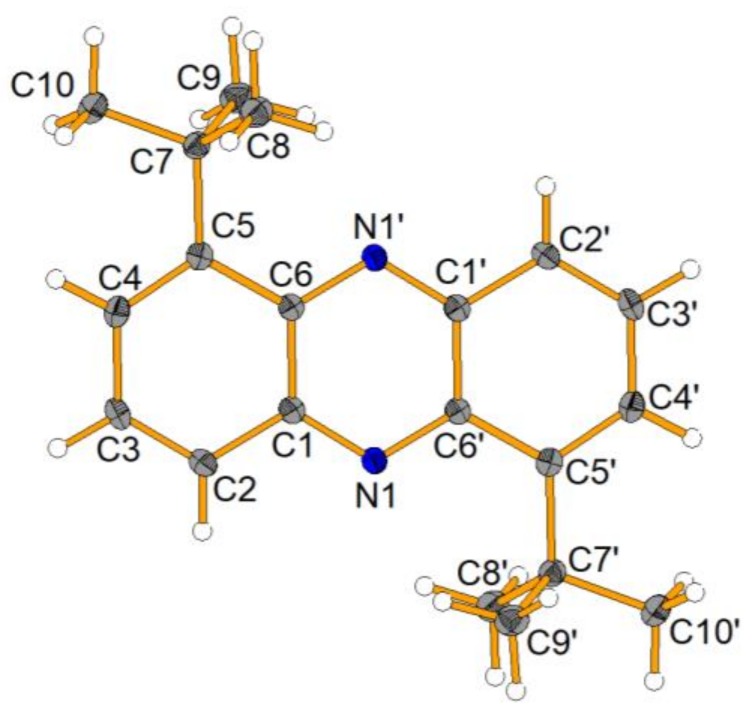
Molecular structure of the isolated phenazine **16**, showing 50% probability ellipsoids.

The product **10** was isolated in a moderate yield of 46%, but surprisingly, we could also isolate a side product, which was characterized by NMR, HRMS, FTIR and XRD and was found to be a phenazine derivative (Scheme 9 and Figure 1). 

It is not clear at present how this side product formed, but the literature suggests that elevated temperature and oxidative conditions favor the formation of the very thermodynamically stable phenazines [27].

With the series of *ortho*-tetrel substituted azobenzenes in hand, we were interested in the comparison of their structures and the effect of the group 14 elements on the physical properties of the azobenzenes. 

**Figure 2:**
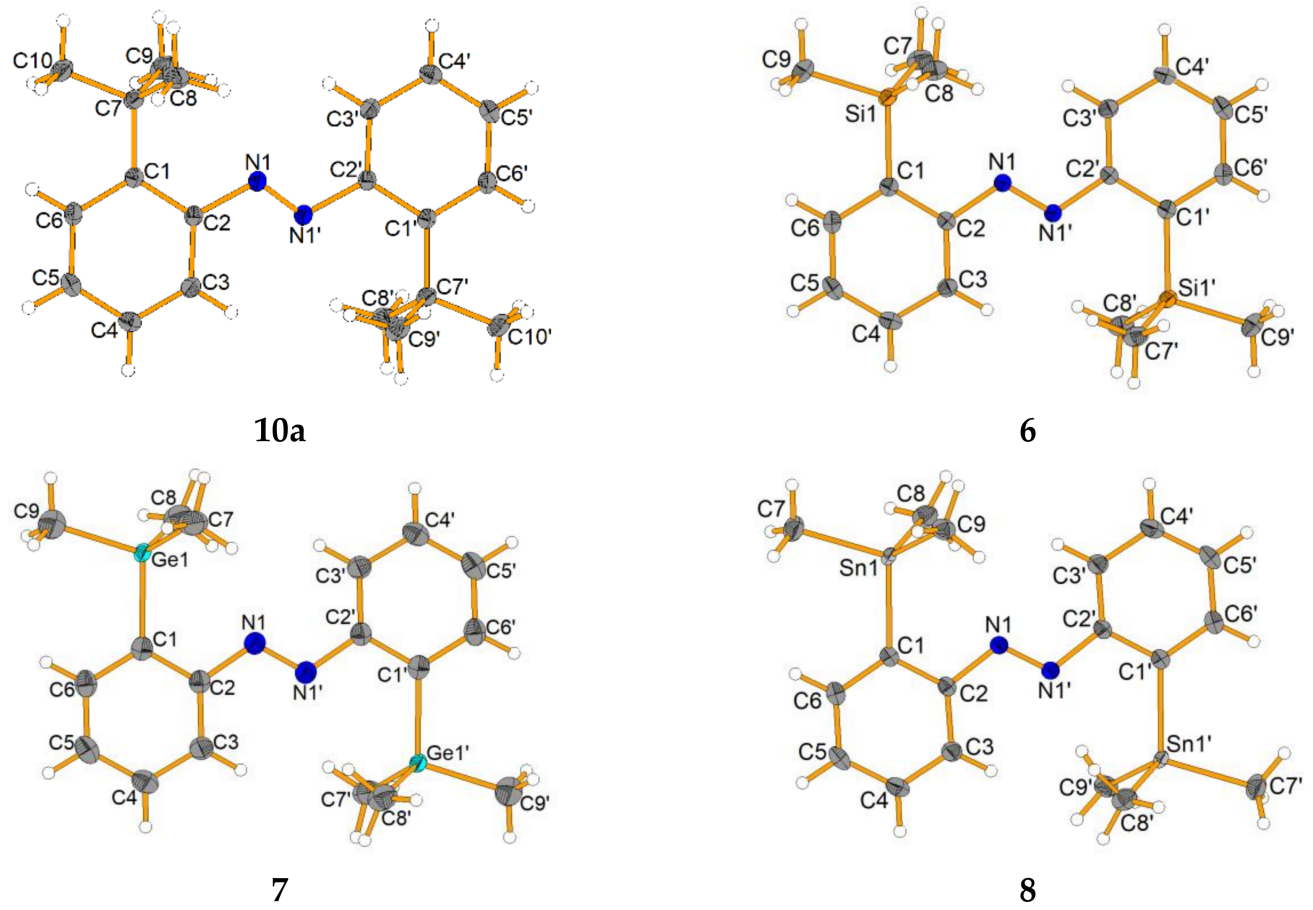
Molecular structures of molecules **10a**, **6**, **7** and **8**, showing a 50% probability of ellipsoids. **10** crystalizes with two molecules in the asymmetric unit, only one is shown (**10a**). The Sn-atom of **8** is disordered over two positions with occupancies of 90% and 10%, respectively. For reasons of clarity only the major component is shown.

The crystal structures of all molecules were determined (Figure 2). Since the interaction of tetrels in the *ortho*-position of azobenzenes has been reported for electron deficient fluorinated silyl groups [42,43], we were interested in how the structural parameters of our crystals would compare with the mono-substituted compounds described by Kano. A special interest was the N=N and carbon-tetrel bond, as well as the possible interaction of the group 14 element with the azo group, which is here indicated by the distance and the torsion from the phenyl ring and the azo group (Table 6).

The *ortho*-substitution had only a marginal effect on the length of the azo group (from 1.2509(18) Å to 1.258(2) Å). However, as expected, the distance of the carbon-tetrel bond increased from the lighter to the heavier elements (1.5348(14) Å (For E = C) to 2.144(1) Å (for E = Sn)). The nitrogen-tetrel distance did not indicate any interaction of the azo group with the respective tetrels (2.9091(18) Å for Si to 3.0471 Å for Sn). This can be compared with azobenzenes with SiMe_2_F (2.585(3) Å) and SiF_3_ (2.371(4) Å) [41]: These much-shortened distances indicated that the lone pair of the nitrogen atom was able to coordinate to the Lewis acidic silicon center, which was not observed in our case. Furthermore, the ^29^Si NMR shift of this compound was to be found at ^29^Si{^1^H} NMR: δ = −4.04 ppm, which is a typical shift for tetracoordinated silicon species. This is well aligned with Kano’s work [41], who reported a ^29^Si{^1^H} NMR-shift of −3.8 ppm for a trimethylsilyl substituted azobenzene, as compared to −16.8 ppm if one of the Me groups was replaced by F, or even −57.8 ppm for a SiF_3_ group in this position. Furthermore, the crystal structure differs in the fact that in the case of a nitrogen-tetrel interaction, a four-membered ring would result, whereas the structures from Kano showed interaction with the second nitrogen atom of the azo group, resulting in a five membered ring; for this to happen, the aromatic rings would need to rotate by approximately 180°, which was not observed. Upon substitution with higher congeners than carbon or silicon, the germanium and tin substituted azobenzene exhibited a carbon-tetrel bond length of 2.0234(18) Å and 2.144(1) Å, which can be considered as an ordinary organotetrel bond length. However, the torsion angles for the CMe_3_ substituted azobenzene were surprisingly far from planar (by ca. 15–17°), which was in contrast to Kano’s work on Si and also our results on the SiMe_3_ substituted compounds, which show almost no distortion. We assumed that the sterical hinderance of the *tert*-butyl group, in comparison with the short C(Ph)–C(CMe_3_) bond, forced the system to undergo distortion. Due to more flexible and longer C–Si, C–Ge and C–Sn bonds, we observed less planar distortion (3–6°).

**Scheme 10:**
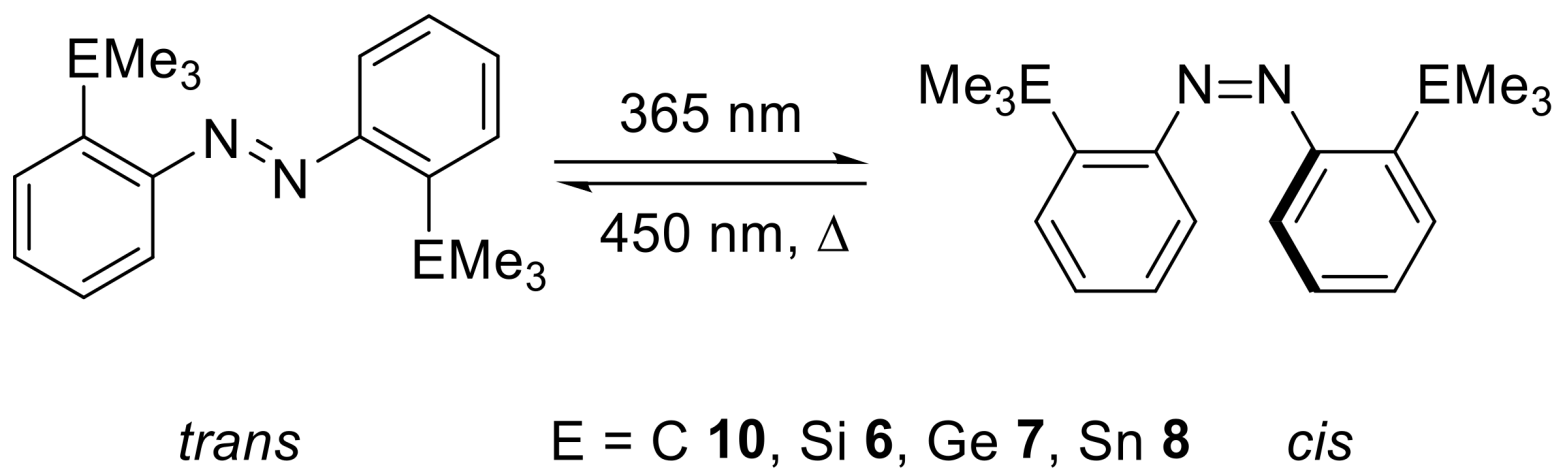
Light/thermally induced isomerization of synthesized azobenzenes **10** and **6**–**8**.

Since azobenzenes can undergo *trans*/*cis* isomerization, we studied the effect of the different group 14 elements on the general absorption properties, the *cis*/*trans* equilibrium in the dark and the photostationary state (PSS) at 365 nm and 450 nm through UV and NMR spectroscopy (Scheme 10).

**Figure 3:**
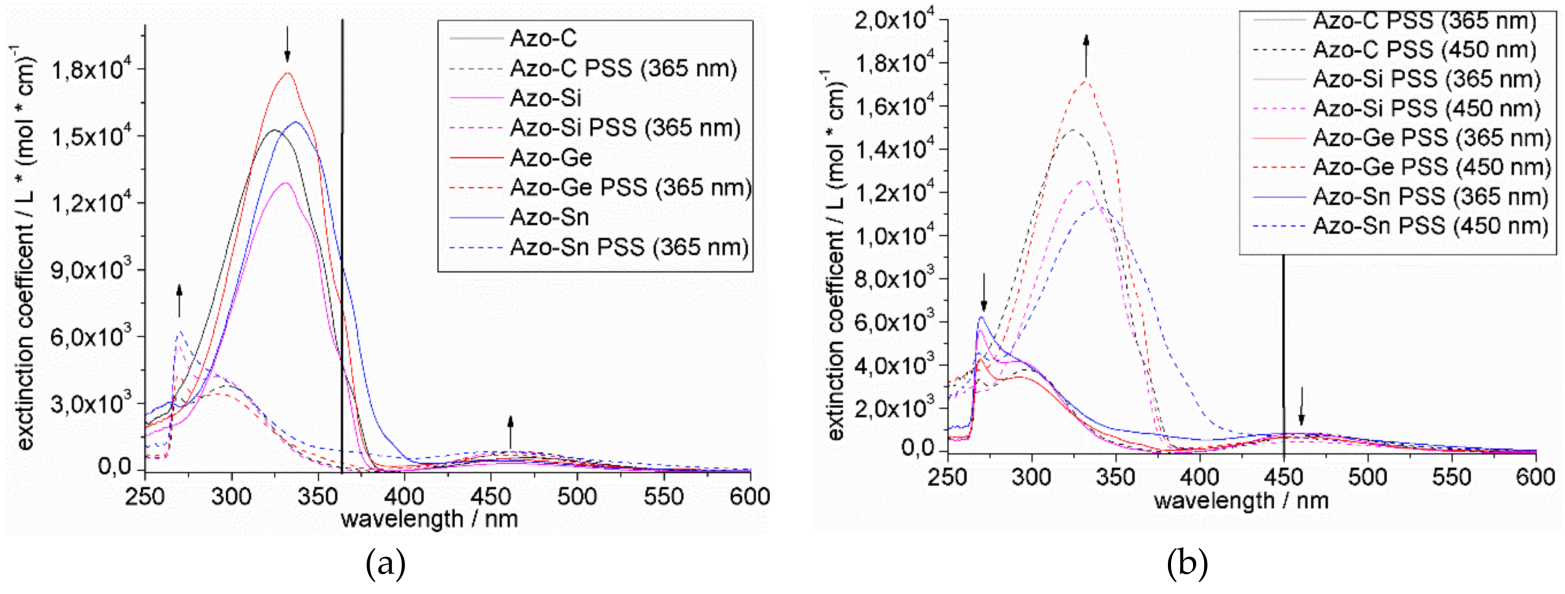
(**a**) Absorption spectra of azobenzenes **6**, **7**, **8** and **10** before and after irradiation with 365 nm light for 3 min. (**b**) Absorption spectra of azobenzenes **6**, **7**, **8** and **10** after irradiation with 365 nm light for 3 min, followed by irradiation with 450 nm light for 3 min. In each case, the black vertical lines indicate the irradiation wavelengths.

In general, the UV spectra of the azobenzenes exhibit a strong ππ* absorption around 330 nm and a weak absorption for the nπ* band of the *cis*-isomer around 475 nm (Figure 3a,b). Upon irradiation of the azobenzenes with UV light (365 nm), we observed a significant decrease in the absorbance of these molecules (Figure 3a), which can be assumed to switch to the respective *cis*-isomer. Blue light (450 nm) shifts the photostationary state (PSS) to the *trans*-isomers (Figure 3b).

In all cases, the switching was totally reversible but the tin-substituted azobenzene **8** showed a decrease and broadening of the ππ* band (Figure 3b), which was not expected, and may indicate a photochemical decomposition process. 

**Figure 4:**
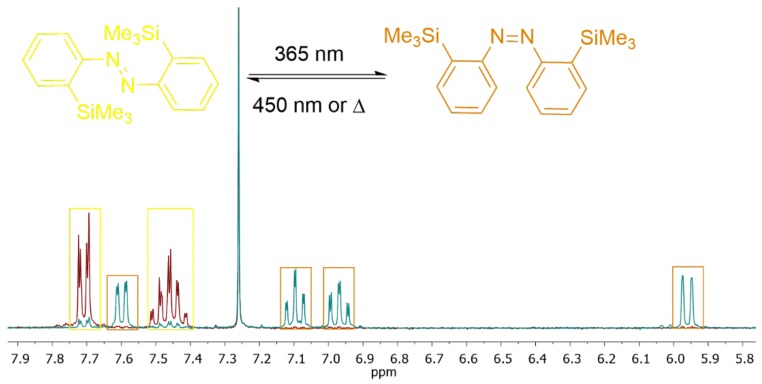
^1^H NMR (300 MHz) spectra of compound **6** before (red) and after (blue) irradiation with 365 nm light (15 min) in CDCl_3_.

To obtain the ratio of the *cis*/*trans*-isomer in the PSS, we combined irradiation experiments with ^1^H NMR analysis. The result of this experiment for molecule **6** is shown below (Figure 4).

To compare the general trends upon substitution with different tetrels on the azobenzenes, we compared the collected isomerization information (Table 7).

Upon substitution of these compounds with heavier group 14 elements, we observed a slight bathochromic shift of the ππ* band (325 nm to 338 nm). This could be explained by the effect of the decreasing torsion angle due to the bulky trimethyltetrel groups, allowing extended π-conjugation (Table 8). However, the effect of the heavier tetrels on the extinction coefficient of the azobenzene system is rather small.

All systems contained only a small amount of the *cis*-isomer ‘as synthesized’. Upon irradiation with 365 nm light, the maximum amount of the *cis*-isomer was found to range from 80% to 91% from E = C to E = Ge, respectively. Therefore, the PSS is substantially influenced by the tetrel. For E = Sn, however, the ratio appeared much lower, but this could also be attributed to photodecomposition. Irradiation with 450 nm light did not result in a complete switching to the *trans*-isomer, which can be observed by the existence of the nπ*-band in the UV-VIS spectra. Unfortunately, irradiation with 365 nm and 450 nm light caused the tin-substituted azobenzene **8** to experience some decomposition, which was detected by UV and ^1^H NMR spectroscopy. This may be caused by a small C–Sn bond-dissociation energy. We further observed the thermal relaxation of the *cis*-azobenzenes that had been obtained after irradiation with 365 nm light with UV-VIS and NMR spectroscopy. The half-life times (τ) in the NMR experiments were determined to be between 55.61 h to 65.84 h, in which an increasing half-life time with heavier tetrels trend could be observed. The UV experiments for compounds **6**, **7** and **10** revealed half-life times of 17.07 h to 20.45 h. In this case, no correlation between the tetrel substitution and the half-life time could be found (for all data see the SI). There is a substantial discrepancy between the half-life times measured by the two techniques. It needs to be pointed out in this context that the concentrations in the NMR were higher by approximately 2 orders of magnitude. Although half-life times should not be concentration dependent in general, they can be different if agglomerations and the stacking of molecules occur, which is likely to be the case for the compounds under investigation.

These systems were also analyzed by dynamic scanning calorimetry (DSC). To ensure thermal stability in the temperature range of investigation of these compounds, thermogravimetric analysis (TGA) was conducted (see SI). The compounds showed mass loss at 208 °C (C, **10**), 188 °C (Si, **6**), 196 °C (Ge, **7**) and 248 °C (Sn, **8**). However, when a TGA experiment on molecule **10** was interrupted at mass loss and the residue was analyzed by NMR spectroscopy, no decomposition was observed. Therefore, we assumed that the azobenzene evaporated at the higher temperature, accounting for the observed loss of mass.

**Figure 5:**
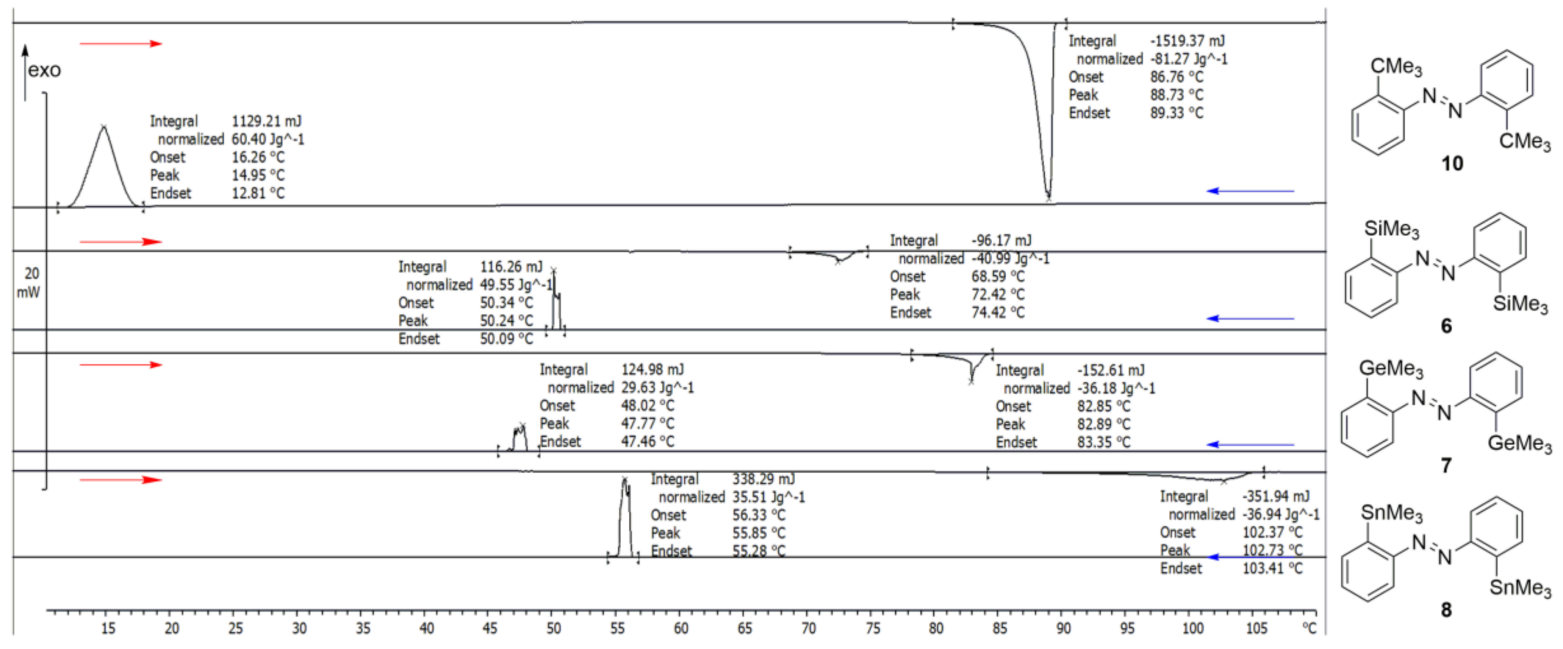
Stacked dynamic scanning calorimetry (DSC) spectra of all target compounds. The DSC experiments were performed with crystals with a heating rate of 0.5 K/min and a flow of nitrogen gas of 20 mL/min. As an example, compound **10** was analyzed after three cycles by ^1^H- and ^13^C{^1^H} NMR spectroscopy and showed no degree of decomposition.

In general, these compounds showed endothermic melting signals at onset temperatures of 87 °C (E = C), 69 °C (E = Si **6**), 83 °C (E = Ge) and 102 °C (E = Sn **8**), and exothermic crystallization peaks of 16 °C (E = C **10**), 50 (E = Si), (48 °C (E = Ge) and 56 °C (E = Sn **8**) (Figure 5, Table 8)). In general, the melting temperature increased within the main group of compounds **6**–**8**, but compound **10** proved to be an exception. Although the temperature ramps were slow (0.5 K/min), the melting peaks were not completely sharp, additionally, the crystallization peaks showed multiple crystallization events due to spontaneous seeding (for all data see the SI).

We observed that with faster DSC measuring modes (10 K/min) there was a high degree of amorphousness in the structures (see SI). With slower DSC methods (0.5 K/min), the difference between the fusion and solidification measured with the slower DSC method enthalpies are still existent and may be related to a non-crystallizing part of the molecules. The fusion enthalpy of compound **10** was 23.93 KJ/mol, which is rather high in comparison of the other azobenzenes (**6**: 13.37 KJ/mol, **7**: 15.13 KJ/mol, **8**: 15.05 KJ/mol). 

## 3. Discussion

We have described the synthesis, characterization (XRD, NMR, HRMS FTIR), switching and thermal behavior for four azobenzenes in detail. The synthesis for azobenzene **8** was conducted via Stille-Kelly cross-coupling with optimized reaction conditions and a high yield. Attempts to synthesize azobenzenes **6** and **7** using the respective Me_6_M_2_ (M = Si, Ge) compound and the same method did not succeed. However, the synthesis for azobenzenes **6** and **7** was performed through tin-lithium exchange with further reaction with the respective trimethyltetrel halides. For *ortho*-halogenated azobenzenes, *ortho*-lithiated species can also be obtained by a simple halogen-metal exchange. The lithium atom *ortho*-position is particularly stabilized by coordination with the *N* atoms of the azogroup, which facilitates the reaction. Also, the first tin-lithium-copper transmetalation on *ortho*-azobenzenes has been tested to enhance the reactivity of the nucleophile towards softer electrophiles. Since the reaction with *tert*-butyl chloride (**13**) (whether via the tin-lithium or tin-lithium-copper transmetalation procedure) was not successful, we decided to obtain azobenzene **10** via the oxidation of the corresponding aniline. Furthermore, as a byproduct, a phenazine **16** could be isolated and completely characterized. Due to the nucleophilic character of the three *ortho*-metalated azobenzenes (Si, Ge, Sn), these molecules could be prospective molecules for cross-coupling reactions, see for example silicon [43,44], germanium [45,46,47] or tin [48,49]. 

## 4. Materials and Methods

### 4.1. General Information

For inert reactions, a nitrogen filled glovebox from Pure Lab^HE^ and standard Schlenk techniques were used. Except for the preparation of the 2,2′-diiodoazobenzene (**2**) and 2,2′-di(*tert*-butyl)azobenzene (**10**), all reactions were carried out under inert conditions.

All glassware for the inert reactions was dried in an oven at 200 °C for several hours prior to use. Additionally, before starting the reaction, the glassware was heated under vacuum (about 5 × 10^−2^ mbar) and flushed at least three times with argon. The NMR tubes were dried at 110 °C for several hours.

Microwave irradiated reactions were carried out using an Emrys^TM^ Optimizer instrument (Biotage, Uppsala, Sweden), in which the temperature was measured with an external IR detector. MeLi was titrated prior to usage with menthol, using 2,2′-bipyridine as an indicator [50].

For chromatographic purification, silica gel 60 (MERCK, Darmstadt, Germany, 0.015–0.40 mm) was used. If stated, Celite^®^ 503 (Carl Roth GmbH & Co. KG, Karlsruhe, Germany) was used as a filtration aid. Thin layer chromatography was performed by using thin layer chromatography (TLC) Silicagel 60 F254 from MERCK. A UV lamp (λ = 254 nm) was used for detection.

NMR spectra were recorded at 300 K on a Bruker AvanceNeo 500 (Bruker, Rheinstetten, Germany) (500 MHz (^1^H), 125 MHz (^13^C{^1^H}), 187 MHz (^119^Sn{^1^H}), 100 MHz (^29^Si {^1^H})). Where possible, NMR signals were assigned using ^1^H COSY, ^1^H/^13^C HSQC and ^1^H/^13^C HMBC experiments. ^1^H and ^13^C {^1^H} NMR spectra (125 MHz) were referenced against the residual solvent signal, CDCl_3_ (^1^H: δ = 7.26 ppm, ^13^C: δ = 77.16 ppm). ^119^Sn{^1^H} and ^29^Si{^1^H} NMR spectra (187 and 100 MHz) were measured based on the external reference of the ^1^H-NMR signal of tetramethylsilane.

Reaction controls by ^1^H-NMR and some ^13^C{^1^H} spectra were performed on a Bruker Avance WB 360 instrument (Bruker, Rheinstetten, Germany) (^1^H: 360 MHz, ^13^C{^1^H}: 91 MHz). The switching experiments were performed on a Bruker ARX300 (Bruker, Rheinstetten, Germany) (300 MHz (^1^H)) or Bruker AvanceNeo 500 (Burker, Rheinstetten, Germany) (500 MHz (^1^H).

Thermal analyses were performed on a standalone Mettler Toledo DSC 3+ STAR (Mettler-Toledo, Columbus, OH, USA) or a Mettler Toledo TGA/DSC 3+ System (Mettler-Toledo, Columbus, OH, USA), where 40 µL and 100 µL aluminum crucibles were used. For TGA experiments, no lids were used, whereas in DSC experiments pierced lids were used. Thermo analytical data was analyzed with the STARe software (Version 14.01, Mettler-Toledo, Columbus, OH, USA) by Mettler Toledo.

Infrared spectra were recorded on a NICOLET i510 FT-IR spectrometer from Thermo Fisher SCIENTIFIC (Thermo Fisher SCIENTIFIC, Waltham, MA, USA) with a diamond window in an area ranging from 500–4000 cm^−1^ with a resolution of 4 cm^−1^. All samples were measured 16 times against a background scan. Melting points were measured by DSC or using a BÜCHI Melting Point M-560 instrument (BÜCHI, Essen, Germany).

Electron impact (EI) mass experiments were measured using the direct inlet or indirect inlet methods, with a source temperature of 200 °C on a MAT95 XL double-focusing mass spectrometer from Finnigan MAT (Thermo Fisher SCIENTIFIC, Waltham, MA, USA). The ionization energy of the electron impact ionization was 70 eV. Atmospheric pressure chemical ionization (APCI) experiments were performed on a Bruker Impact II from Bruker Daltonics (Bruker Daltonics, Bremen, Germany).

For the UV switching experiments, a 365 nm (Ocean Optics USB 4000, Sahlmann Photochemical Solutions, Bad Segeberg, Germany, full width at half maximum (FWHM) 10 nm, 1.0 W) and a 443 nm LED lamp (Ocean Optics USB 4000, Sahlmann Photochemical Solutions, Bad Segeberg, Germany, FWHM 19 nm, 0.9 W) were used, while ensuring a constant distance towards the cuvettes of 1 cm. The NMR switching experiments were performed with a circular aligned lamp consisting of four high power UV-LEDs (365 nm) 300 mW in power each, produced by “Sahlmann Photochemichal Solutions”. For switching experiments, quartz cuvettes from Hellma Analytics (Hellma Analytics, Muehlheim an der Ruhr, Germany) (10 mm) or Quartz NMR tubes from Deutero (Deutero GmbH, Kastellaun, Germany) were used.

UV-VIS spectra were recorded at a resolution of 0.1 nm on a UV-2700 spectrometer from Shimadzu (Shimadzu, Kyoto, Japan) with a double monochromator. In all cases, cyclohexane was used as a solvent.

X-ray measurements were carried out at 100 K on a Bruker Venture D8 diffractometer (Bruker, Karlsruhe, Germany) with Mo-Kα (0.7107 Å) radiation. All structures were solved by intrinsic phasing and refined based on F2 by use of the SHELX program package, as implemented in OLex 1.2 [51]. All non-hydrogen atoms were refined using anisotropic displacement parameters. Hydrogen atoms attached to carbon atoms were included in geometrically calculated positions using a riding model. All crystals were obtained by slow evaporation of an acetonitrile/dichloromethane mixture at 25 °C.

### 4.2. Syntheses

#### 4.2.1. 2,2′-Diiodoazobenzene (**2**)

Adapted with changes from Takahashi et al. [26]. A solution of 2-iodoaniline (**1**) (2.00 g, 9.13 mmol) and MnO_2_ (20.0 g, 230 mmol) in toluene (200 mL) was stirred for 3 h at 120 °C. After the reaction, the mixture was cooled to 25 °C, filtered through Celite^®^, washed with toluene (200 mL) and the solvent was removed under reduced pressure. The red solid that was obtained was dissolved in DCM (5 mL) and purified by filtration through a short plug of silica (eluent: *n*-pentane). After evaporation of the solvent, the dark orange solid was dried (4.6 × 10^−2^ mbar, 25 °C, 4 h) to give a red compound (1.63 g, 3.76 mmol, 82%).

^1^H NMR (500 MHz, CDCl_3_) δ = 8.04 (dd, ^3^*J* = 7.9 Hz, ^4^*J* = 1.3 Hz, 2H, H-3), 7.77 (dd, ^3^*J* = 7.9 Hz, ^4^*J* = 1.6 Hz, 2H, H-6), 7.46 (ddd, ^3^*J* = 7.9, 7.3 Hz, ^4^*J* = 1.3 Hz, 2H, H-5), 7.20 (ddd, ^3^*J* = 7.9, 7.3 Hz, ^4^*J* = 1.6 Hz, 2H, H-4) ppm. ^13^C{^1^H} NMR (126 MHz, CDCl_3_) δ = 150.87 (C-1), 139.94 (C-3), 132.80 (C-4), 129.06 (C-5), 118.26 (C-6), 103.22 (C-2) ppm. IR (ATR): ν = 3055 (w), 2921 (w), 2851 (w), 1924 (w), 1838 (w), 1806 (w), 1699 (w), 1561 (m), 1455 (m), 1013 (s), 953 (m), 760 (s), 714 (s) cm^−1^. HRMS (EI, MAT 95 XL): *m/z* calcd. C_12_H_8_N_2_I_2_^+^ 433.87715 found 433.87701. R*_f_* (*n*-pentane): 0.51. Mp (Büchi): 146 °C

#### 4.2.2. 2,2′-Bis(trimethylstannyl)azobenzene (**8**)

Adapted with changes from Strüben et al. [25] In a glovebox, a microwave vial was charged with 2,2′-diiodoazobenzene (**2**) (200 mg, 0.46 mmol), hexamethylditin (**5**) (422 mg, 1.03 mmol), tetrakis(triphenyl)phosphinopalladium(0) (15.7 mg, 13.0 µmol), THF (0.5 mL) and toluene (4.0 mL). The reaction mixture was heated for 1 h at 170 °C using microwave irradiation. The solution was cooled to 25 °C, filtered, rinsed with toluene (10.0 mL) and all volatiles were removed under reduced pressure. Then, the compound was dissolved in DCM (2 mL) and filtered through a short plug of silica (eluent: *n*-pentane). After the solvent was removed, an orange solid (142 mg, 0.28 mmol, 61%) was received. ^1^H NMR (500 MHz, CDCl_3_) δ = 7.78 (dd, ^3^*J* = 7.8 Hz, ^4^*J* = 1.4 Hz, 2H, H-6), 7.74 (dd, ^3^*J* = 7.1 Hz, ^4^*J* = 1.4 Hz, 2H, H-3), 7.47 (td, ^3^*J* = 7.1 Hz, ^4^*J* = 1.4 Hz, 2H, H-5), 7.43 (td, ^3^*J* = 7.1 Hz, ^4^*J* = 1.4 Hz, 2H, H-4), 0.32 (s, 18H, CH_3_) ppm. ^13^C{^1^H} NMR (126 MHz, CDCl_3_) δ = 157.12 (C-1), 146.53 (C-2), 136.65 (C-3), 130.33 (C-4), 129.52 (C-2), 117.73 (C-6), −7.33 (C-7) ppm. ^119^Sn{^1^H} NMR (187 MHz, CDCl_3_) −34.36 ppm. IR (ATR): ν = 3050 (w), 2974 (w), 2909 (w), 2609 (w), 2354 (w), 1965 (w), 1932 (w), 1902 (w), 1853 (w), 1820 (w), 1432 (w), 1294 (w), 1188 (m), 1110 (m), 754 (s), 706 (s) cm^−1^. HRMS (APCI): *m/z* calcd. [C_18_H_26_N_2_Sn_2_ + H]^+^ 509.02140 found 509.02126. R*_f_*: (*n*-pentane): 0.84. Mp (DSC; Onset): 102.37 °C

#### 4.2.3. 2,2′-Bis(trimethylsilyl)azobenzene (**6**)

In an inert tube 2,2′-bis(trimethylstannyl)azobenzene (**4**) (80.0 mg, 0.16 mmol) was dissolved under Schlenk conditions in THF (5.00 mL) and cooled to −78 °C. MeLi (1.88 M in THF, 0.25 mL, 0.47 mmol) was added within 5 min and after 1 h at this temperature, trimethylsilyl chloride (**9**) (200 µL, 171 mg, 1.57 mmol) was added to the black reaction mixture in one portion. The reaction mixture was warmed to 25 °C over 14 h and the solvent was removed under reduced pressure. The brown solid, dissolved in DCM (3.00 mL), was purified by a short plug of silica (eluent: *n*-pentane). The first orange fraction was filtered through a PTFE filter (0.45 µm). From the filtrate, the solvent was removed to obtain an orange solid (43 mg, 0.132 mmol, 82%). ^1^H NMR (500 MHz, CDCl_3_): δ = 7.72 (dd, ^3^*J* = 7.7 Hz, ^4^*J* = 1.3 Hz, 4H, H-3 and H-6), 7.48 (ddd, ^3^*J* = 7.7, 7.2 Hz, ^4^*J* = 1.3 Hz, 2H, H-5), 7.44 (ddd, ^3^*J* = 7.7, 7.2 Hz, ^4^*J* = 1.3 Hz, 2H, H-4), 0.40 (s, 18H, C*H*_3_) ppm. ^13^C{^1^H} NMR (125 MHz, CDCl_3_): δ = 157.27 (C-1), 142.95 (C-2), 134.97 (C-3), 130.14/130.11 (C-4 and C-5), 114.68 (C-6), 0.70 (C-7) ppm. ^29^Si{^1^H} NMR (100 MHz, CDCl_3_): δ = 4.04 ppm. IR (ATR): ν = 3059 (w), 2946 (w), 2987 (w), 2853 (w), 1968 (w), 1937 (w), 1859 (w), 1737 (w), 1581 (w), 1561 (w), 1465 (w), 1424 (w); 1296 (w), 1241 (m), 1119 (m), 1075 (w), 831 (s), 778 (s), 747 (m), 720 (s), 676 (m) cm^−1^. HRMS(EI): *m/z* calcd. C_18_H_26_N_2_Si_2_^+^ 326.16290 found 326.16245. R*_f_* (*n*-pentane): 0.63. Mp (DSC; Onset): 68.59 °C

#### 4.2.4. 2,2′-Bis(trimethylgermanyl)azobenzene (**7**)

A schlenk tube was filled with 2,2′-bis(trimethylstannyl)azobenzene (**4**) (80.0 mg, 0.16 mmol) in THF (5.00 mL) and cooled to −78 °C. Then, MeLi (1.88 M in THF, 0.25 mL, 0.47 mmol) was added within 5 min and after 1 h at this temperature, trimethylgermanium chloride (**14**) (200 µL, 171 mg, 1.57 mmol) was added to the dark reaction mixture in one portion. The reaction mixture was warmed to 25 °C over 14 h and the solvent was removed under reduced pressure. The brown solid, dissolved in DCM (3.00 mL), was purified by column chromatography (silica, *n*-pentane). From the filtrate, the solvent was removed to obtain an orange solid (31 mg, 0.09 mmol, 60%). ^1^H NMR (500 MHz, CDCl_3_): δ = 7.78–7.64 (m, 4H, H-3 and H-6), 7.49–7.39 (m, 4H, H4 and H5), 0.49 (s, 18H, C*H*_3_) ppm. ^13^C{^1^H} NMR (125 MHz, CDCl_3_): δ = 156.59 (C-1), 146.12 (C-2), 134.21 (C-3), 130.11 (C-4), 129.52 (C-2), 114.98 (C-6), 0.32 (C-7) ppm. IR (ATR): ν = 3057 (w), 2962 (w), 2905 (w), 1563 (w), 1463 (w), 1432 (w), 1407 (w), 1295 (w), 1234 (m), 1114 (m), 1064 (w), 953 (w), 818 (m), 777 (s), 751 (m), 719 (m), 658 (m) cm^−1^. HRMS (APCI): *m/z* calcd. [C_18_H_26_N_2_Ge_2_ + H]^+^ 417.06085 found 417.06072. R*_f_* (*n*-pentane): 0.78. Mp (DSC; Onset): 82.85 °C

#### 4.2.5. 2,2′-Di(*tert*-butyl)azobenzene (**10**)

Adapted with changes from Takahashi et al. [26] 2-*tert*-butylaniline (**15**) (5.00 g, 33.5 mmol) was dissolved in toluene (800 mL) and MnO_2_ (50.0 g, 575 mmol) was added portionwise. The reaction mixture was heated to 120 °C. After 3 h at 120 °C, the orange reaction mixture was filtered with the help of Celite^®^. The solvent was evaporated, the organic phase was washed with aq. HCl (2 M, 300 mL), and then the organic phase was dried over MgSO_4_. After removal of the solvent, the product was dried in a vacuum (4 mbar, 110 °C, 5h) and could be isolated without further purification (2.26 g, 7.68 mmol, 46%, purity > 95%). For even higher purity, the compound was subjected to column chromatography (silica, *n*-pentane). The first isolated band was the mentioned yellow byproduct (62 mg, 0.22 mmol, 1.31%). The second band was the orange product (620 mg, 2.11 mmol, 13%). ^1^H NMR (500 MHz, CDCl_3_) δ = 7.54–7.50 (m, 4H, H-3 and H-6), 7.40 (td, ^3^*J* = 7.6 Hz, ^4^*J* = 1.4 Hz 2H, H-5), 7.32 (mc, 2H, H-4), 1.56 (s, 18 H, CH_3_).^13^C{^1^H} NMR (126 MHz, CDCl_3_): δ = 151.93 (*C*-1), 148.60 (*C*-2), 130.37 (*C*-5), 126.80 (*C*-4), 126.76 (*C*-3), 117.05 (*C*-6), 36.33 (*C*(CH_3_)_3_, 32.16 (CH_3_) ppm. IR (ATR): ν = 2948 (w), 2918 (w), 2904 (w), 2859 (w), 1478 (m), 1465 (w), 1435 (w), 1388 (w), 1354 (w), 1289 (w), 1277 (w), 1250 (w), 1196 (w), 1160 (w), 1086 (w), 1050 (w), 947 (w), 926 (w), 769 (s), 750 (s) cm^−1^. HRMS (EI): *m/z* calcd. C_20_H_26_N_2_^+^ 294.20905 found 294.20851. R*_f_* (*n*-pentane): 0.76. Mp (DSC; Onset): 86.76 °C

#### 4.2.6. Isolated By-Product: 1,6-Di-(*tert*-butyl)phenanzine (**16**)

^1^H NMR (500 MHz, CDCl_3_) δ = 8.11 (m_c_, 2H, H-4,9), 7.70 (m_c_, 4H, H-3,8 and H-2,7), 1.76 (s, 18H, CH_3_) ppm. ^13^C{^1^H} NMR (126 MHz, CDCl_3_) δ = 148.46 (*C*-1,6), 142.19 (*C*-4a,10a or *C*-5a,10), 141.89 (*C*-4a, 10a or *C*-5a,10a), 129.48 (*C*-3,8), 129.01 (*C*-4,9), 126.05 (*C*-2,7), 36.97 (*C*(CH_3_)_3_, 31.22 (CH_3_) ppm. IR (ATR): ν = 2997 (w), 2949 (w), 2902 (w), 2864 (w), 1616 (w), 1532 (w), 1481 (w), 1471 (w), 1383 (w), 1353 (w), 1343 (w), 1271 (w), 1126 (w), 997 (m), 932 (w), 857 (w), 813 (m), 748 (s) cm^−1^. HRMS (EI): *m/z* calcd. C_20_H_24_N_2_^+^ 292.19340 found 292.19284, calcd. [M-CH_3_]^+^ 277.16993 found 277.16993, calcd. [M-C_3_H_6_]^+^ 250.14645 found 250.14621, calcd. [M-C_4_H_9_]^+^ 235.12297 found 235.12263. R*_f_* (*n*-pentane): 0.81. Mp (DSC; Onset): 193.89 °C

## 5. Conclusions

Four 2,2′-bis(trimethylelement)azobenzenes have been synthesized with E = C, Si, Ge and Sn. The synthetic route that has to be selected is highly dependent on the element: With carbon, substitution reactions with a nucleophilic azobenzene and a carbon electrophile were unsuccessful because of the small size of the carbon atom and its low reactivity. With Si and Ge however, a dilithiated azobenzene or the corresponding cuprate could be used as a precursor, illustrating the higher electrophilic character. However, this species can only easily be produced from a stannylated precursor, which can be prepared through a Stille-Kelly cross-coupling reaction. While the latter procedure proceeds very well for tin, a similar reaction was not possible for the corresponding hexamethyldigermanium and hexamethyldisilane. The lead azobenzene could not be isolated at all, which we ascribe to the instability of Pb(IV) compared to Pb(II).

## Figures and Tables

**Table 1:** Overview of used substrates and reagents for the synthesis of dimetalated azobenzenes.

Entry	M	Additive	Time/h	Yield
1	Si	-	1/8	0%, no conversion ^a^
2	Si	2.00 equiv. Cs_2_CO_3_	1	0%, no conversion ^a^
3	Ge	-	1/8	0%, no conversion ^b^
4	Ge	2.00 equiv. Cs_2_CO_3_	1	0%, no conversion ^b^
5	Sn	-	1	60% isolated product ^c^
6	Pb	-	1	not performed ^d^

^a^ For a similar procedure, see References [29,30]. ^b^ For a similar procedure, see Reference [31]. ^c^ For a similar procedure used for azobenzenes, see References [24,25]. ^d^ This toxic substance decomposes at higher temperatures [32].

**Table 2:** Overview of the Stille-Kelly reaction optimization for the di-stannylated azobenzene **8**.

Entry	Cat. Load	Temp.	Time/h	Yield
1	4 mol%	130 °C, conv. Heating	8	17%
2	4 mol%	130 °C, conv. Heating	16	19%
3	6 mol%	170 °C, μW	1	31%
4	4 mol%	170 °C, µW	1	27%
5	2 mol%	170 °C, µW	1	49%
6	3 mol%	170 °C, µW	1	60%
7	3 mol% ^a^	170 °C, µW	1	7%
8	11 mol%	170 °C, µW	1	14%
9	11 mol% ^b^	170 °C, µW	1	96%

^a^ Copper(I) chloride (6.0 equiv.) and lithium chloride (12.0 equiv.) were added. ^b^ 6.70 equiv. Sn_2_Me_6_ was used.

**Table 3:** Optimization of the tin-lithium exchange and the reaction with **9**.

Entry	T	Lithiation Time	Yield ^a^
1	−78 °C	60 min	87%
2	−60 °C	60 min	55%
3	−40 °C	60 min	49%
4	−20 °C	60 min	20%
5	0 °C	60 min	5%
6	−78 °C	1 min	69%
7	−78 °C	3 min	64%
8	−78 °C	9 min	71%
9	−78 °C	30 min	74%
10	−78 °C	90 min	79%

^a^ Yields were determined by using 1,3,5-tri*iso*-propylbenzene as an internal reference for ^1^H NMR analysis.

**Table 4:** To widen the scope of the transmetalation to softer electrophiles, an organocuprate was introduced by using CuCN · 2 LiCl as softer organometallic reagent. The transmetalation temperature varied from −78 °C to −40 °C.

Entry	E	Metal	Metalation T.	Yield ^a^
1	Me_3_SiCl	[Li]	−78 °C	87%
2	Me_3_SiCl	[Li]	−40 °C	73%
3	Me_3_SiCl	[Cu]	−78 °C	90%
4	Me_3_SiCl	[Cu]	−40 °C	7%
5	Me_3_SiI	[Li]	−78 °C	50% ^b^
6	Me_3_SiI	[Cu]	−78 °C	17% ^b^
7	Me_3_SiI	[Cu]	−40 °C	30% ^b^
8	MeI	[Cu]	−78 °C	5%
9	MeI	[Cu]	−40 °C	56%
10	MeI	[Li]	−78°C	0%

^a^ Yields were determined by using 1,3,5-tri*iso*-propylbenzene as an internal reference for ^1^H NMR analysis. ^b^ As a side reaction, THF might have undergone a ring-opening ether cleavage to form 4-iodo-butyloxytrimethylsilane [40].

**Table 5:** Transmetalation of di-stannylated azobenzene **8** with methyl lithium or further metalation to the cuprate and the reactions of these organometallic reagents with electrophilic trimethyltetrel species.

Entry	E	Metal	Yield
1	Me_3_CCl	[Li] or [Cu]	both 0%
2	Me_3_SiCl	[Li] or [Cu]	87%/90%
3	Me_3_GeCl	[Li] or [Cu]	60%/0% ^a^
4	Me_3_PbBr	[Li] or [Cu]	both 0%

^a^ The reaction resulted in a complex mixture of products in which **7** could not be isolated.

**Table 6:** Overview of the important crystallographic parameters of compounds **10**, **6**, **7** and **8**.

	10a ^1^	10b ^1^	6	7	8
Crystal system	Triclinic		Monoclinic		
Space group	P-1		P2_1_/c		
N-N bond length (Å)	1.2556(17)	1.2509(18)	1.258(2)	1.256(3)	1.2511(19)
C1-tetrel bond length (Å)	1.5348(14)	1.5374(14)	1.8856(14)	2.0234(18)	2.144(1) ^2^
N1-tetrel distance (Å)	2.9402(16)	2.909118	3.0151(11)	3.0441(14)	3.047(1) ^2^
Torsion angle C1-C2-N1-N1′ (°)	−163.3(1)	−164.8(1)	175.0(1)	177.1(2)	−177.3(1)
CCSD No.	1880127		1880130	1880129	1880128

^1^ The asymmetric unit consists of two crystallographic independent molecules. ^2^ Distances to main component are given.

**Table 7:** Collected data of the absorption spectra and switching experiments.

Compound	ππ* Band ε (L mol^−1^ cm^−1^)	PSS (as Synthesized) NMR *cis*:*trans*	PSS (365 nm) *cis*:*trans*	τ_1/2_ NMR (h)	τ_1/2_ UV (h)
**10** (C)	15272 (325 nm)	1:99	80:20	55.61	17.07
**6** (Si)	12907 (331 nm)	1:99	83:17	62.17	20.45
**7** (Ge)	17818 (333 nm)	1:99	91:9	65.84	16.75
**8** (Sn)	15632 (338 nm)	1:99	73:27 ^a^	11.46 ^a^	17.38 ^a^

^a^ Compound **8** presumably underwent decomposition upon irradiation.

**Table 8:** Results of the thermoanalytic experiments.

	10	6	7	8
T_fusion_ (Onset) (°C)	86.76	68.59	82.85	102.37
H_fusion_ (KJ/mol)	23.93	13.37	15.13	15.05
T_solidif._ (Onset) (°C)	16.26	50.34	48.02	56.33
H_solidif._ (KJ/mol)	−17.78	−16.16	−12.39	−18.03

**Figure A1:**
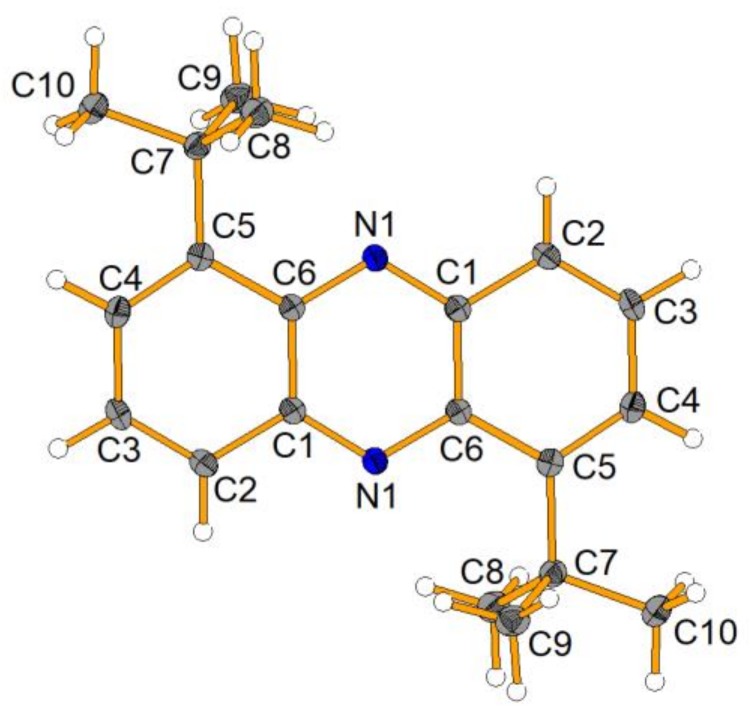
Molecular structure of phenazine **16**, showing a 50% probability of ellipsoids.

## Supplementary Materials

The following are available online at http://www.mdpi.com/1420-3049/24/2/303/s1: A list of all reagents and solvents, attempted syntheses, images of all NMR spectra, UV-VIS spectra and ^1^H NMR spectra of all switching experiments, images of the data of all thermoanalytical experiments (DSC and TGA) and ^1^H NMR spectra of the samples after the DSC experiments.

## Appendix A

### X-Ray Crystallography

There are only a few crystal structures reported for di-*ortho*-substituted azobenzenes: A structure with methyl groups in *ortho*-position [52], with fluorodimethylsilyl groups [43] and boron derivatives [42] have been reported. Compared with the N=N bond in pure *trans*-azobenzene (1.249(4) Å) [52], the azo groups in our compounds have very similar N=N bond lengths (**10**: 1.251(2), **6**: 1.257(3), **7**: 1.256(3), **8**: 1.251(2) Å. The Crystal data and structure refinement for azobenzenes **10**, **6**–**8** are given (Table A1); therefore, these substituents have hardly any influence on the character of the azo group.

**Table A1:** Crystal data and structure refinement for azobenzenes **10**, **6**–**8**.

	10	6	7	8
Chemical formula	C_20_H_26_N_2_	C_18_H_26_N_2_Si_2_	C_18_H_26_N_2_Ge_2_	C_18_H_26_N_2_Sn_2_
Formula weight (g mol^−1^)	294.43	326.59	415.59	507.79
Crystal system	Triclinic	Monoclinic	Monoclinic	Monoclinic
Space group	P-1	P2_1_/c	P2_1_/c	P2_1_/c
a (Å)	8.6142(2)	10.8005(3)	10.8027(5)	10.7087(3)
b (Å)	9.7497(2)	6.9267(2)	7.0849(3)	7.1755(2)
c (Å)	11.5720(3)	13.1555(3)	13.0359(6)	13.1481(4)
α (°)	70.3510(10)	90	90	90
β (°)	71.0950(10)	91.0610(10)	91.1550(10)	91.1950(10)
γ (°)	77.6290(10)	90	90	90
V (Å^3^)	859.85(4)	984.02(5)	997.51(8)	1010.08(5)
Z	2	2	2	2
Dcalc (g cm^−3^)	1.137	1.102	1.384	1.670
µ (mm^−1^)	0.066	0.179	3.012	2.472
F (000)	320.0	352.0	424.0	496.0
Crystal size (mm^3^)	0.27 × 0.24 × 0.14	0.25 × 0.10 × 0.10	0.32 × 0.25 × 0.19	0.20 × 0.10 × 0.10
2Theta range for data collection (°)	4.468 to 62.148	6.194 to 56.992	7.524 to 61.08	6.198 to 59.982
Reflections. collected	51512	32222	9934	64977
Independent Reflections	5512	2500	3018	2937
Final R indexes [I >= 2simga(I)]	R_1_ = 0.0471, wR_2_ = 0.1086	R_1_ = 0.0356, wR_2_=0.0785	R_1_ = 0.0247, wR_2_ = 0.0675	R_1_ = 0.0127, wR_2_ = 0.0287
Final R indexes [all data]	R_1_ = 0.0755, wR_2_ = 0.1240	R_1_ = 0.0560, wR_2_ = 0.0889	R_1_ = 0.0278, wR_2_ = 0.0699	R_1_ = 0.0156, wR_2_ = 0.0299
GooF (F^2^)	1.015	1.045	1.065	1.081
Largest diff. peak/hole (e Å^−3^)	0.39/−0.23	0.29/−0.28	0.76/−0.42	0.48/−0.50
CCSD No.	1880127	1880130	1880129	1880128

So far, only one of the crystal structures of 2,6-dimethoxy substituted phenazines was reported [53]. In the following, the structural parameters for our obtained phenazine are given (Figure A1, Table A2).

**Table A2:** Crystal data and structure refinement for phenazine **16**.

	16
Chemical formula	C_20_H_24_N_2_
Formula weight (g mol^−1^)	292.41
Temperature (K)	100
Crystal system	Monoclinic
Space group	P2_1_/c
a (Å)	12.4209(4)
b (Å)	6.2653(2)
c (Å)	12.3389(4)
α (°)	90
β (°)	119.2690(10)
γ (°)	90
V (Å^3^)	837.63(5)
Z	2
D_calc_ (g cm^−3^)	1.159
µ (mm^−1^)	0.068
F (000)	316.0
Crystal size (mm^3^)	0.25 × 0.17 × 0.15
2Theta range for data collection (°)	6.604 to 56.996
Reflections. collected	20978
Independent Reflections	2132
Final R indexes [I >= 2simga(I)]	R_1_ = 0.0417, wR_2_ = 0.0948
Final R indexes [all data]	R1 = 0.0632, wR_2_ = 0.1082
GooF (F^2^)	1.024
Largest diff. peak/hole (e Å^−3^)	0.33/−0.19
CCSD No.	1880126

CCDC 1880126-1880130 (**16**, **10**, **8**, **7**, **6**) contain the supplementary crystallographic data for this paper. The data can be obtained free of charge from The Cambridge Crystallographic Data Centre via www.ccdc.cam.ac.uk/structures.

## References

[1] Rivard E. (2016). Group 14 inorganic hydrocarbon analogues. Chem. Soc. Rev..

[2] Yamaguchi S., Tamao K. (1996). Theoretical Study of the Electronic Structure of 2,2′-Bisilole in Comparison with 1,1′-Bi-1,3-cyclopentadiene: σ*-π* Conjugation and a Low-Lying LUMO as the Origin of the Unusual Optical Properties of 3,3′,4,4′-Tetraphenyl-2,2′-bisilole. Bull. Chem. Soc. Jpn..

[3] Yamaguchi S., Itami Y., Tamao K. (1998). Group 14 metalloles with thienyl groups on 2,5-positions:  Effects of group 14 elements on their π-electronic structures. Organometallics.

[4] Linshoeft J., Baum E.J., Hussain A., Gates P.J., Näther C., Staubitz A. (2014). Highly tin-selective stille coupling: synthesis of a polymer containing a stannole in the main chain. Angew. Chem. Int. Ed..

[5] Urrego-Riveros S., Ramirez y Medina I.-M., Hoffmann J., Heitmann A., Staubitz A. (2017). Syntheses and properties of tin-containing conjugated heterocycles. Chem. Eur. J..

[6] Ramirez y Medina I.-M., Rohdenburg M., Mostaghimi F., Grabowsky S., Swiderek P., Beckmann J., Hoffmann J., Dorcet V., Hissler M., Staubitz A. (2018). Tuning the optoelectronic properties of stannoles by the judicious choice of the organic substituents. Inorg. Chem..

[7] Bandara H.M.D., Burdette S.C. (2012). Photoisomerization in different classes of azobenzene. Chem. Soc. Rev..

[8] Beharry A.A., Woolley G.A. (2011). Azobenzene photoswitches for biomolecules. Chem. Soc. Rev..

[9] Dong M., Babalhavaeji A., Samanta S., Beharry A.A., Woolley G.A. (2015). Red-shifting azobenzene photoswitches for in vivo use. Acc. Chem. Res..

[10] Mahimwalla Z., Yager K.G., Mamiya J.-I., Shishido A., Priimagi A., Barrett C.J. (2012). Azobenzene photomechanics: Prospects and potential applications. Polym. Bull..

[11] Merino E., Ribagorda M. (2012). Control over molecular motion using the cis-trans photoisomerization of the azo group. Beilstein J. Org. Chem..

[12] Ahmed Z., Siiskonen A., Virkki M., Priimagi A. (2017). Controlling azobenzene photoswitching through combined ortho-fluorination and -amination. Chem. Commun..

[13] Bléger D., Schwarz J., Brouwer A.M., Hecht S. (2012). o-Fluoroazobenzenes as readily synthesized photoswitches offering nearly quantitative two-way isomerization with visible light. J. Am. Chem. Soc..

[14] Yu Y., Nakano M., Ikeda T. (2003). Directed bending of a polymer film by light. Nature.

[15] Kizilkan E., Strueben J., Jin X., Schaber C.F., Adelung R., Staubitz A., Gorb S.N. (2016). Influence of the porosity on the photoresponse of a liquid crystal elastomer. Roy. Soc. Open Sci..

[16] Nguyen T.H.L., Gigant N., Joseph D. (2018). Advances in direct metal-catalyzed functionalization of azobenzenes. ACS Catal..

[17] Kakiuchi F., Matsumoto M., Tsuchiya K., Igi K., Hayamizu T., Chatani N., Murai S. (2003). The ruthenium-catalyzed silylation of aromatic C–H bonds with triethylsilane. J. Organomet. Chem..

[18] Nguyen T.T.T., Boussonnière A., Banaszak E., Castanet A.-S., Nguyen K.P.P., Mortier J. (2014). Chemoselective deprotonative lithiation of azobenzenes: Reactions and mechanisms. J. Org. Chem..

[19] Li J., Cong W., Gao Z., Zhang J., Yang H., Jiang G. (2018). Rh(III)-catalyzed regioselective mono- and di-iodination of azobenzenes using alkyl iodide. Org. Biomol. Chem..

[20] Yamamura M., Kano N., Kawashima T., Matsumoto T., Harada J., Ogawa K. (2008). Crucial role of N···Si interactions in the solid-state coloration of disilylazobenzenes. J. Org. Chem..

[21] Kano N., Komatsu F., Kawashima T. (2001). Synthesis and structure of azobenzenes bearing silyl, germyl, and stannyl groups at 2-position. Chem. Lett..

[22] Kano N., Komatsu F., Yamamura M., Kawashima T. (2006). Reversible photoswitching of the coordination numbers of silicon in organosilicon compounds bearing a 2-(phenylazo)phenyl group. J. Am. Chem. Soc..

[23] Strueben J., Hoffmann J., Naether C., Staubitz A. (2016). Crystal structures of 3,3’-bis(hydroxydimethylsilanyl)azobenzene and 4,4’-bis(hydroxydimethylsilane)azobenzene. Acta Cryst. E.

[24] Strueben J., Lipfert  M., Springer  J.-O., Gould C.A., Gates P.J., Sönnichsen F.D., Staubitz A. (2015). High-yield lithiation of azobenzenes by tin-lithium exchange. Chem. Eur. J..

[25] Strueben J., Gates P.J., Staubitz A. (2014). Tin-functionalized azobenzenes as nucleophiles in stille cross-coupling reactions. J. Org. Chem..

[26] Takahashi H., Ishioka T., Koiso Y., Sodeoka M., Hashimoto Y. (2000). Anti-androgenic activity of substituted azo- and azoxy-benzene derivatives. Bio. Pharm. Bull..

[27] Seth K., Roy S.R., Kumar A., Chakraborti A.K. (2016). The palladium and copper contrast: A twist to products of different chemotypes and altered mechanistic pathways. Catal. Sci. Tech..

[28] Monir K., Ghosh M., Mishra S., Majee A., Hajra A. (2013). Phenyliodine(III) diacetate (PIDA) mediated synthesis of aromatic azo compounds through oxidative dehydrogenative coupling of anilines: Scope and mechanism. Eur. J. Org. Chem..

[29] Gooßen L.J., Ferwanah A.-R.S. (2000). A mild and efficient protocol for the catalytic silylation of aryl bromides. Synlett.

[30] McNeill E., Barder T.E., Buchwald S.L. (2007). Palladium-catalyzed silylation of aryl chlorides with hexamethyldisilane. Org. Lett..

[31] Komami N., Matsuoka K., Yoshino T., Matsunaga S. (2018). Palladium-catalyzed germylation of aryl bromides and aryl triflates using hexamethyldigermane. Synthesis.

[32] Arnold D.P., Wells P.R. (1976). Hexamethyldilead: I. Preparation, thermal decomposition and methanolysis. J. Organomet. Chem..

[33] Zavitsas A.A. (2003). The relation between bond lengths and dissociation energies of carbon–carbon bonds. J. Phys. Chem..

[34] Lappert M.F., Pedley J.B., Simpson J., Spalding T.R. (1971). Bonding studies of compounds of boron and the group IV element: VI. Mas spectrometric studies on compounds Me_4_M and Me_3_M–M′Me_3_ (M and M′ = C, Si, Ge, Sn, and Pb): Thermochemical data. J. Organomet. Chem..

[35] Dávalos J.Z., Baer T. (2006). Thermochemistry and dissociative photoionization of Si(CH_3_)_4_, BrSi(CH_3_)_3_, ISi(CH_3_)_3_, and Si_2_(CH_3_)_6_ studied by threshold photoelectron−photoion coincidence spectroscopy. J. Phys. Chem. A.

[36] Farina V., Kapadia S., Krishnan B., Wang C., Liebeskind L.S. (1994). On the nature of the “copper effect” in the stille cross-coupling. J. Org. Chem..

[37] Casado A.L., Espinet P., Gallego A.M. (2000). Mechanism of the stille reaction. 2. Couplings of aryl triflates with vinyltributyltin. Observation of intermediates. A more comprehensive scheme. J. Am. Chem. Soc..

[38] Aggarwal V.K., Staubitz A.C., Owen M. (2006). Optimization of the mizoroki−heck reaction using design of experiment (DoE). Org. Process. Res. Dev..

[39] Knochel P., Yeh M.C.P., Berk S.C., Talbert J. (1988). Synthesis and reactivity toward acyl chlorides and enones of the new highly functionalized copper reagents RCu(CN)ZnI. J. Org. Chem..

[40] Krueerke U. (1962). Halogen-austausch an chlorsilanen und die tetrahydrofuran-spaltung durch brom- und jodsilane. Chem. Ber..

[41] Olaru M., Kather R., Hupf E., Lork E., Mebs S., Beckmann J. (2018). A monoaryllead trichloride that resists reductive elimination. Angew. Chem. Int. Ed..

[42] Yoshino J., Kano N., Kawashima T. (2013). Fluorescent azobenzenes and aromatic aldimines featuring an N–B interaction. Dalton Trans..

[43] Kano N., Yamamura M., Kawashima T. (2015). 2,2’-Disilylazobenzenes featuring double intramolecular nitrogen···silicon coordination: A photoisomerizable fluorophore. Dalton Trans..

[44] Denmark S.E., Regens C.S. (2008). Palladium-catalyzed cross-coupling reactions of organosilanols and their salts: practical alternatives to boron- and tin-based methods. Acc. Chem. Res..

[45] Hatanaka Y., Hiyama T. (1988). Cross-coupling of organosilanes with organic halides mediated by a palladium catalyst and tris(diethylamino)sulfonium difluorotrimethylsilicate. J. Org. Chem..

[46] Kosugi M., Tanji T., Tanaka Y., Yoshida A., Fugami K., Kameyama M., Migita T. (1996). Palladium-catalyzed reaction of 1-aza-5-germa-5-organobicyclo[3.3.3]undecane with aryl bromide. J. Organomet. Chem..

[47] Faller J.W., Kultyshev R.G. (2002). Palladium-catalyzed cross-coupling reactions of allyl, phenyl, alkenyl, and alkynyl germatranes with aryl iodides. Organometallics.

[48] Nakamura T., Kinoshita H., Shinokubo H., Oshima K. (2002). Biaryl synthesis from two different aryl halides with tri(2-furyl)germane. Org. Lett..

[49] Cordovilla C., Bartolomé C., Martínez-Ilarduya J.M., Espinet P. (2015). The stille reaction, 38 years later. ACS Catal..

[50] Tanaka R., Kawahara T., Shinto Y., Nakayama Y., Shiono T. (2017). An alternative method for the preparation of trialkylaluminum-depleted modified methylaluminoxane (dMMAO). Macromolecules.

[51] Dolomanov O.V., Bourhis L.J., Gildea R.J., Howard J.A.K., Puschmann H. (2009). OLEX2: A complete structure solution, refinement and analysis program. J. Appl. Crystallogr..

[52] Harada J., Ogawa K., Tomoda S. (1997). Molecular Motion and Conformational Interconversion of Azobenzenes in Crystals as Studied by X-ray Diffraction. Acta Cryst. Sec. B.

[53] Nawata Y., Iwasaki H., Saito Y. (1967). The Crystal Structure of Bis(pyridine-2-carboxamido)nickel(II) Dihydrate. Bull. Chem. Soc. Jpn..

