# Supplementary material for: Synthesis, Structure, Thermal Behavior and cis/trans Isomerization of 2,2′-(EMe3)2 (E = C, Si, Ge, Sn) Substituted Azobenzenes
Source: Molecules. 2019 Jan 15;24(2):303. doi: 10.3390/molecules24020303 (PMC6359049; doi:10.3390/molecules24020303)
Supplement: Supplementary file 1 [file molecules-24-00303-s001.pdf]

Supporting Information

Synthesis, Structure, Thermal Behavior and *cis/trans* Isomerization of 2,2'-$(EMe_3)_2$ (E = C, Si, Ge, Sn) Substituted Azobenzenes

J. Hoffmann[1,2,3,4], T. J. Kuczmera[1], E. Lork[5], A. Staubitz[1,2,3]

[1] Institute for Analytical and Organic Chemistry, University of Bremen, Leobener Straße 7, D-28359 Bremen, Germany, jonas.hoffmann@uni-bremen.de (J.H.); kuczmera@uni-bremen.de (T.F.K.); staubitz@uni-bremen.de (A.S.)

[2] MAPEX Center for Materials and Processes, University of Bremen, Bibliothekstraße 1, D-28359 Bremen, Germany

[3] Otto-Diels-Institute for Organic Chemistry, University of Kiel, Otto-Hahn-Platz 4, D-24098 Kiel, Germany

[4] Université Rennes, CNRS, ISCR - UMR 6226, 263 Av. du Général Leclerc, F-35042 Rennes, France

[5] Institute for Inorganic Chemistry and Crystallography, University of Bremen, Leobener Straße 7, D-28359 Bremen, Germany; enno.lork@uni-bremen.de (E.L.)

Corresponding author: staubitz@uni-bremen.de

Table of Contents

1. Reagents and Solvents **2**

2. Syntheses **3**

*a. Direct lithiation of 2,2-diiodoazobenzene (**2**) with n-butyllithium and quenching with trimethylsilyl chloride (**9**)* *3*

*b. Reaction of the nucleophilic azobenzene **8** with the electrophilic azobenzene **2** under cross coupling conditions* *3*

*c. Attempted synthesis of 2,2'-bis(trimethyllead)azobenzene (**11**)* *4*

3. ^{1}H, $^{13}C\{^{1}H\}$, $^{29}Si\{^{1}H\}$ and $^{119}Sn\{^{1}H\}$ NMR Spectra **6**

4. UV/Vis Spectra and ^{1}H NMR Spectra of the Switching Experiments **14**

5. Thermoanalysis (DSC and TGA) and ^{1}H / $^{13}C\{^{1}H\}$ NMR Spectra of the Respective Compounds After Thermoanalysis **27**

1. Reagents and Solvents

All reagents were used without purification unless stated otherwise.

Chemical	Supplier	Purity	Comments
1,3,5-Tri*iso*propylbenzene	Sigma Aldrich	95%	stored in a glovebox
2-Iodoaniline	TCI	>98%	-
2,2'-Bipyridine	Sigma Aldrich	99%	stored in a glovebox
Bromotrimethyllead(IV)	Sigma Aldrich	97%	stored in a glovebox
n-Butyllithium	ACROS Organics		1.6 M in hexanes
CuCl	Alfa Aesar	>97%	stored in a glovebox
CuCN	Sigma Aldrich	98%	stored in a glovebox
Hexamethyldigermanium	Merck	techn.	stored in a glovebox
Hexamethyldisilane	ABCR	97%	stored in a glovebox
Hexamethylditin	ABCR	99%	stored in a glovebox
LiCl	Sigma Aldrich	99%	stored in a glovebox
MeI	Sigma Aldrich	99%	stored in a glovebox
MeLi	ACROS Organics		1.88 M in diethyl ether
Menthol	MERCK	99%	-
MnO_2	MERCK	>80 %	activated
$[Pd(PPh)_3)_4]$	TCI	>97%	stored in a glovebox
Trimethylsilyl chloride	Sigma Aldrich	99%	stored in a glovebox
Trimethylgermanium chloride	TCI	>98	stored in a glovebox
2-*tert*-Butylaniline	ACROS Organics	98%	-

All solvents for purification and extraction were used as received. In the case of *n*-pentane, the solvent was distilled prior use.

All solvents which were used for synthesis under inert conditions were dried by a solvent purification system from Inert Technologies.

Solvent	Supplier/ Drying Agent
Acetonitrile	Fisher Scientific, analytical grad / -
Chloroform-d_1	Eurisotop, 99.8% / -
Dichlormethane	VWR, HPLC grade / SPS
Dimethylformamide	Acros / stored over molecular sieves
n-Pentane	VWR, techn. grade / SPS
Toluene	Fisher Scientific, HPLC grade / SPS
Tetrahydrofuran	VWR, HPLC grade / SPS

2. Syntheses

a. Direct lithiation of 2,2-diiodoazobenzene (**2**) with *n*-butyllithium and quenching with trimethylsilyl chloride (**9**)

The method was adapted from Kano et al.[1] and used for 2,2'-Diiodo-4,4'-dimethylazobenzene.

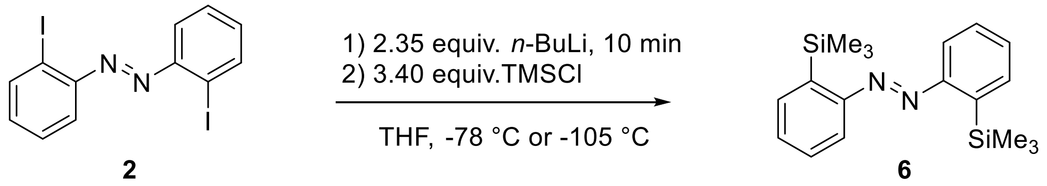

Scheme SI1. Direct lithiation of the iodo-azobenzene **2** with *n*-butyllithium and further reaction with trimethylsilyl chloride (**9**).

In a 100 mL Schlenk flask, 2,2'-diiodoazobenzene (**2**) (1.02 g, 2.35 mmol) was dissolved in THF (30 mL) and the NMR standard 1,3,5-tri*iso*propylbenzene (480 mg, 2.35 mmol) was added. After cooling the reaction mixture to the given temperature *n*-BuLi (3.1 mL, 5.00 mmol, 1.6 M in hexane) was added in one portion resulting in a dark color. Then, trimethylsilyl chloride (**9**) (1.02 mL, 8.00 mmol) was added rapidly. The reaction mixture was warmed to 25 °C over a period of 12 h. After evaporation of all volatiles the yield was found to be 95% for the reaction at -78°C and 99% for the reaction performed at -105°C by ^{1}H NMR spectroscopy.

b. Reaction of the nucleophilic azobenzene **8** with the electrophilic azobenzene **2** under cross coupling conditions

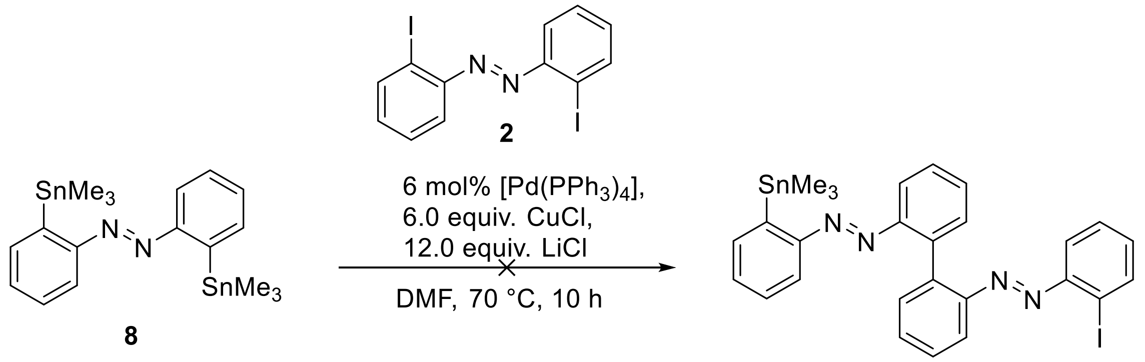

Scheme SI2. The reaction of the stannylated azobenzene **8** with azobenzene **2** under the previously used coupling conditions did not lead to any conversion.

In a glovebox, a pressure tube (total volume = 15.0 mL) was filled with **8** (75 mg, 0.15 mmol), **2** (65.1 mg, 150 µmol) $[Pd(PPh_3)_4]$ (6.80 mg, 59.0 µmol), LiCl (75.0 mg, 1.77 mmol), CuCl (88.1 mg, 890 µmol) and DMF (6.00 mL). The vial was caped and heated for 10 h at 70 °C. The solution was cooled to 25 °C, chloroform (30 mL) was added and the mixture was extracted with hydrochloric acid (2 M, 2 x 55 mL), a saturated sodium carbonate solution (55 mL) and water (55 mL). The combined organic phases were dried over sodium sulfate, filtered, and the solvent was evaporated. A ^{1}H NMR experiment revealed that no reaction had occurred.

[1] Yamamura, M.; Kano, N.; Kawashima, T.; Matsumoto, T.; Harada, J.; Ogawa, K., Crucial Role of N···Si Interactions in the Solid-State Coloration of Disilylazobenzenes. *J. Org. Chem.* **2008,** 73, (21), 8244-8249, doi:10.1021/jo801334a.

c. Attempted synthesis of 2,2'-bis(trimethyllead)azobenzene (**11**)

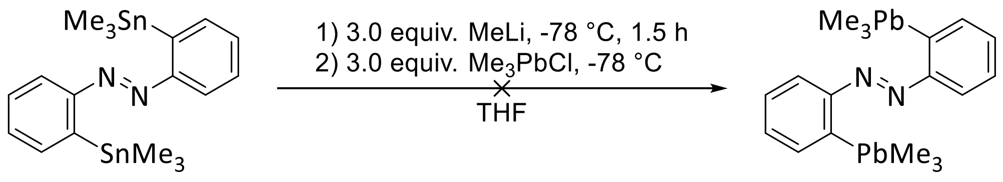

8 **11**

Scheme SI3. Attempt to synthesize plumbinated azobenzene **11** via a tin-lithium exchange followed by quenching with a lead electrophile.

Via tin-lithium-exchange

In a J. Young's tube, **8** (100 mg, 200 µmol) was dissolved in THF (10 mL) and cooled to -78 °C. Then MeLi (0.36 mL, 0.60 mmol, 1.66 M in Et_2O) was added over the course of 5 min. The reaction mixture turned black. After 60 min at this temperature, Me_3PbBr (200 mg, 1.26 mmol, dissolved in 2 mL of THF) was added in one portion. After 10 min at this temperature, the color changed to brown. The reaction mixture was warmed to 23 °C for 14 h, stirred for 2 d, to result in an orange color with a dark precipitate in the flask. Afterwards the solvent was removed; ^1H NMR analysis revealed an undefined mixture of products. The crude product was subjected to column chromatography (silica, pentane) but this purification attempt did not give any product.

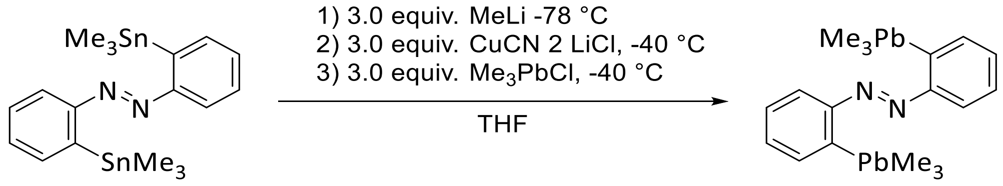

8 **11**

Scheme SI4. Attempt to synthesize plumbinated azobenzene **11** via a tin-lithium exchange reaction followed by transmetallation to copper, followed by quenching with a lead electrophile.

Via tin-lithium-copper transmetalation

In a glovebox, CuCN (215 mg, 2.40 mmol) and LiCl (203 mg, 4.80 mmol) were dissolved in THF (5.0 mL) to obtain a CuCN 2 LiCl solution.

In a J. Young's tube, **8** (406 mg, 800 µmol) was dissolved in THF (20 mL) and cooled to -78 °C. Then MeLi (1.45 mL, 2.40 mmol, 1.66 M in Et_2O) was added over the course of 5 min. The reaction mixture turned black. After 90 min at this temperature the CuCN 2 LiCl –solution (see above) was added at -40 °C. After 1 h, Me_3PbBr (800 mg, 2.40 mmol, dissolved in 5 mL of THF) was added in one portion. The reaction mixture was warmed to 23 °C overnight (9 h). To the black mixture, an aqueous saturated NH_4Cl solution (0.2 mL) was added and the mixture turned orange. The reaction vessel was internally coated with a black precipitate, which was insoluble in organic solvents. The reaction mixture was dried over $MgSO_4$, filtered, and all volatiles were removed i. vac.. ^1H NMR analysis of the crude mixture revealed a complex mixture of products, which could not be identified. After filtration over a short plug

of silica with pentane as eluent a yellow oil (25 mg) could be obtained. Further analysis revealed that no aliphatic proton signals remained.

3. ^{1}H, $^{13}C\{^{1}H\}$, $^{29}Si\{^{1}H\}$ and $^{119}Sn\{^{1}H\}$ NMR Spectra

2,2’-Diiodoazobenzene (**2**)

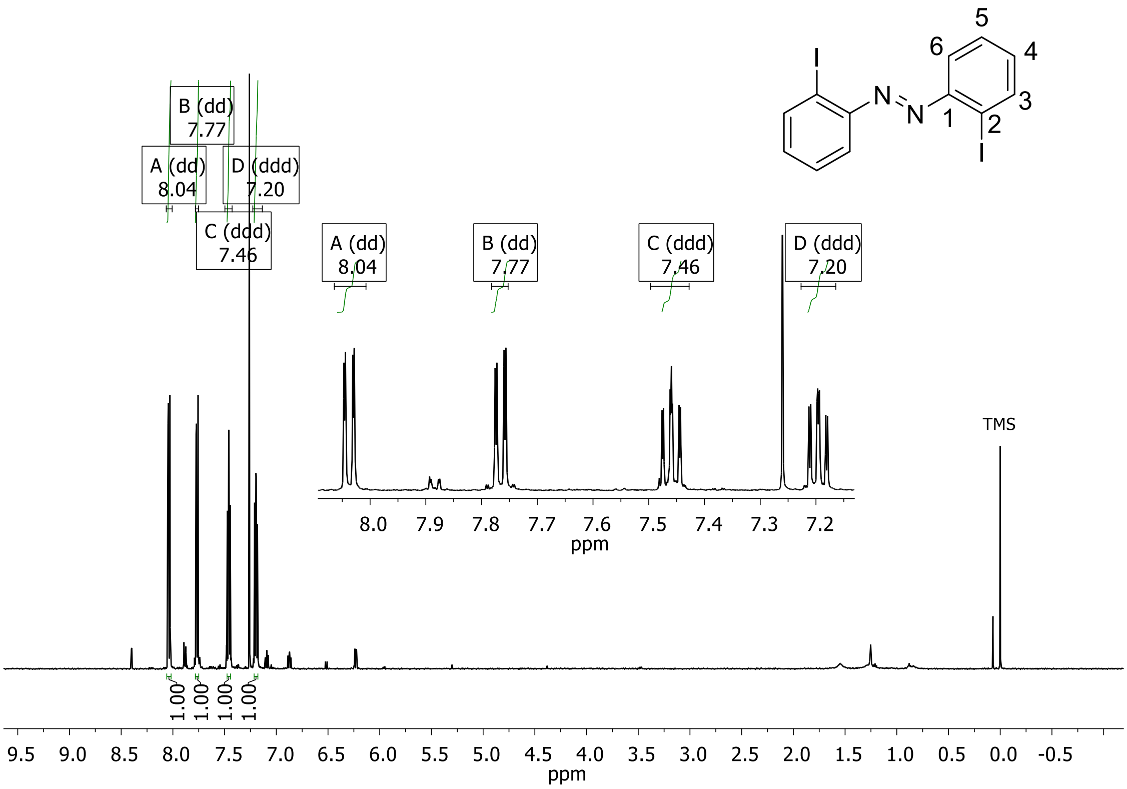

Figure SI1: ^{1}H NMR spectrum of compound **2**. The less intense signals in the aromatic region can be assigned to the *cis*-isomer.

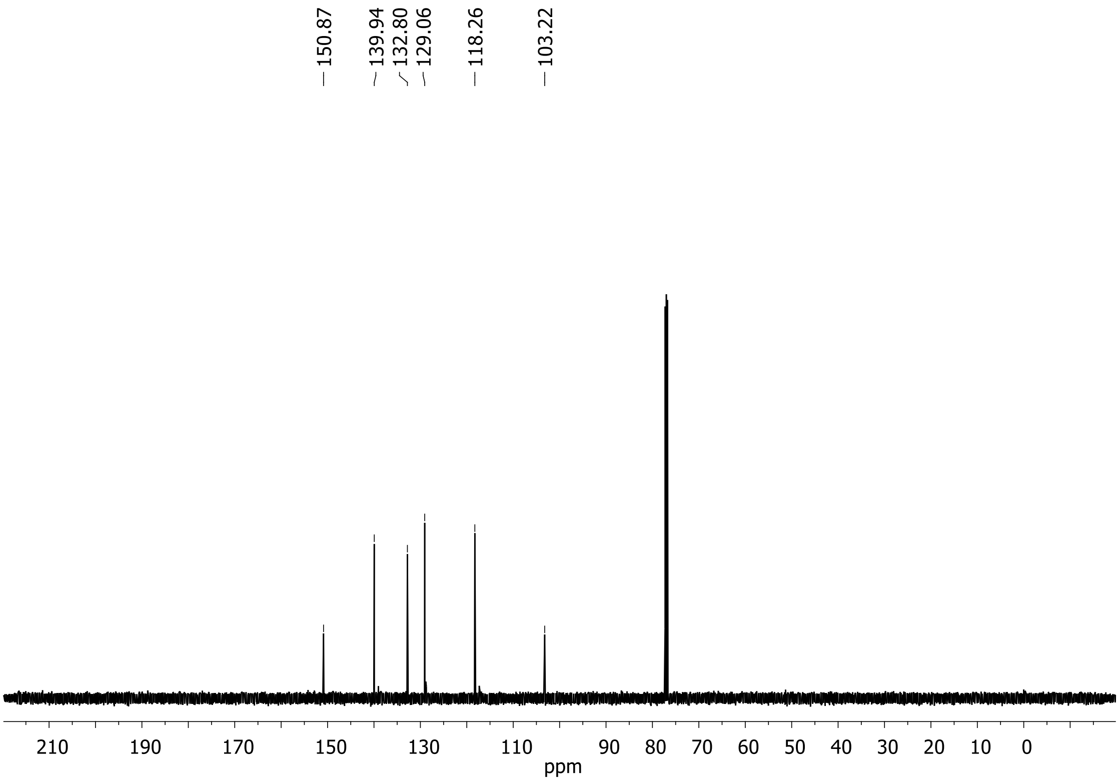

Figure SI2: $^{13}C\{^{1}H\}$ NMR spectrum of compound **2**.

2,2'-Di(*tert*-butyl)azobenzene (**10**)

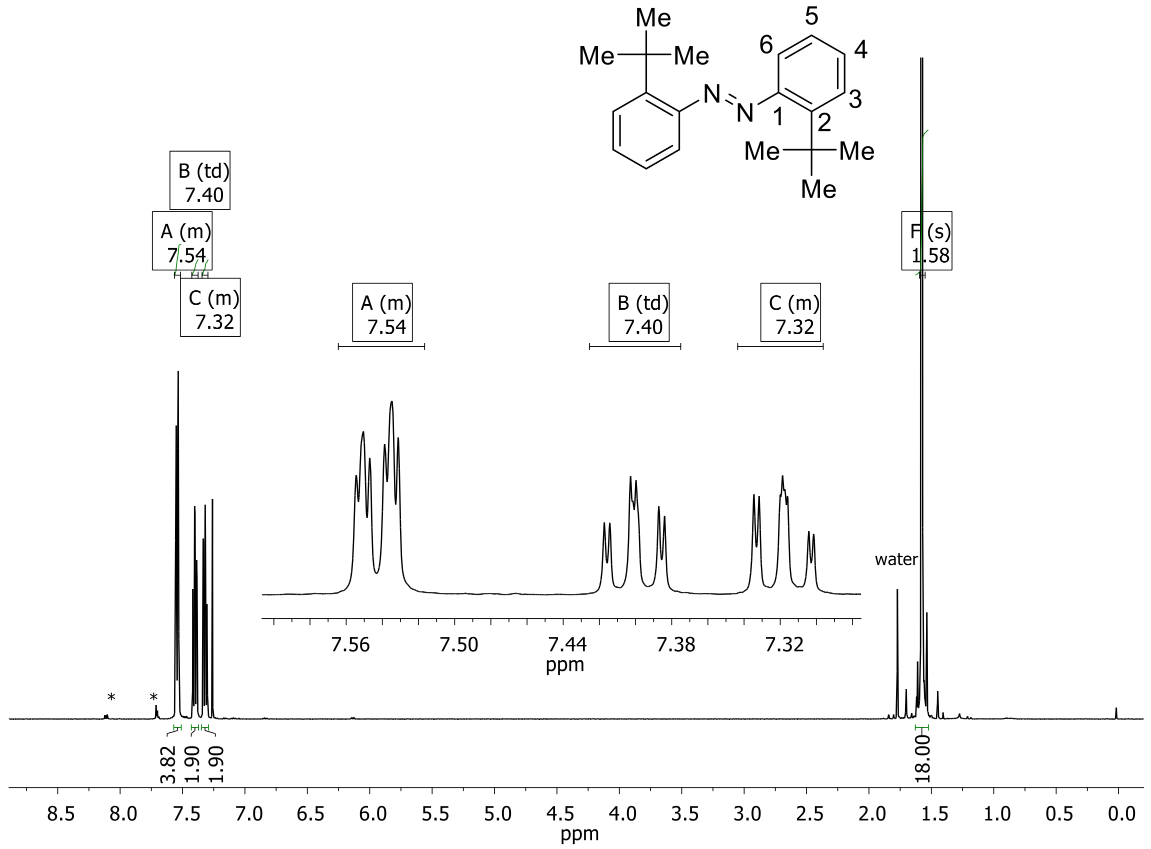

Figure SI3: ^{1}H NMR spectrum of compound **10**. The less intense signals in the aromatic region can be assigned to the *cis*-isomer and traces of compound **16** (indicated with a star).

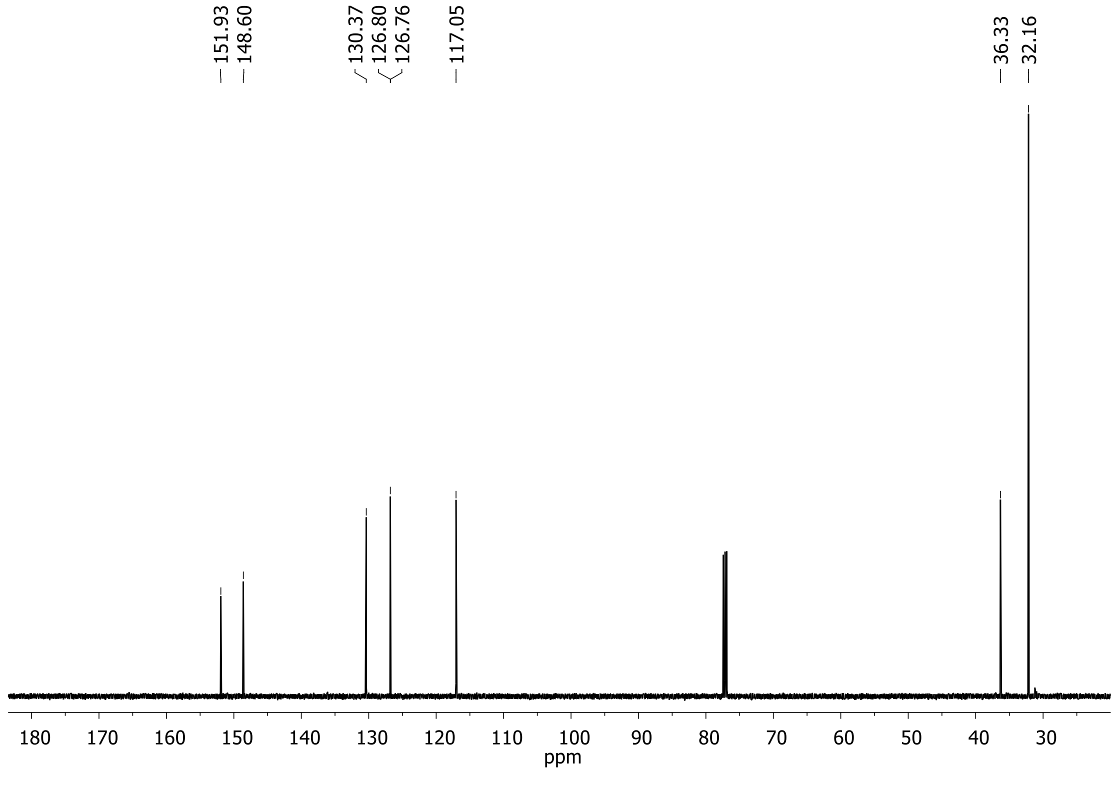

Figure SI4: $^{13}C\{^{1}H\}$ NMR spectrum of compound **10**.

1,6-Di(*tert*-butyl)phenanzine (**16**)

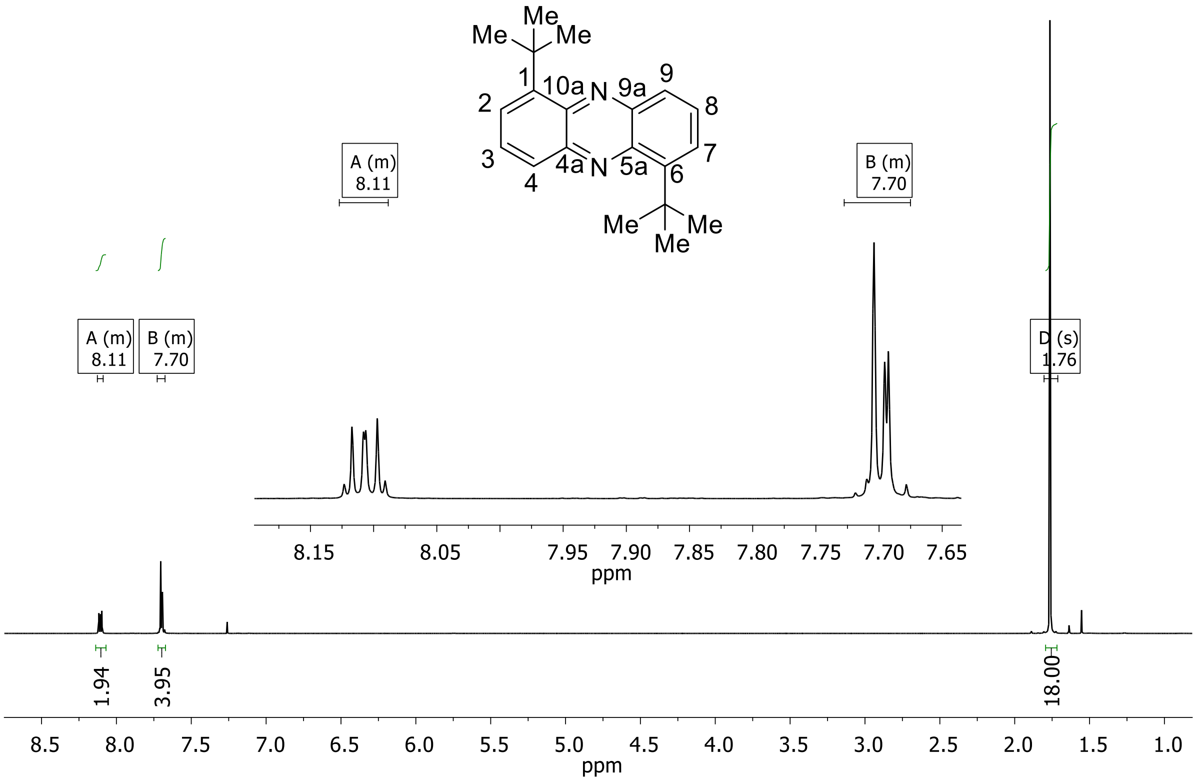

Figure SI5: ^{1}H NMR spectrum of compound **16**.

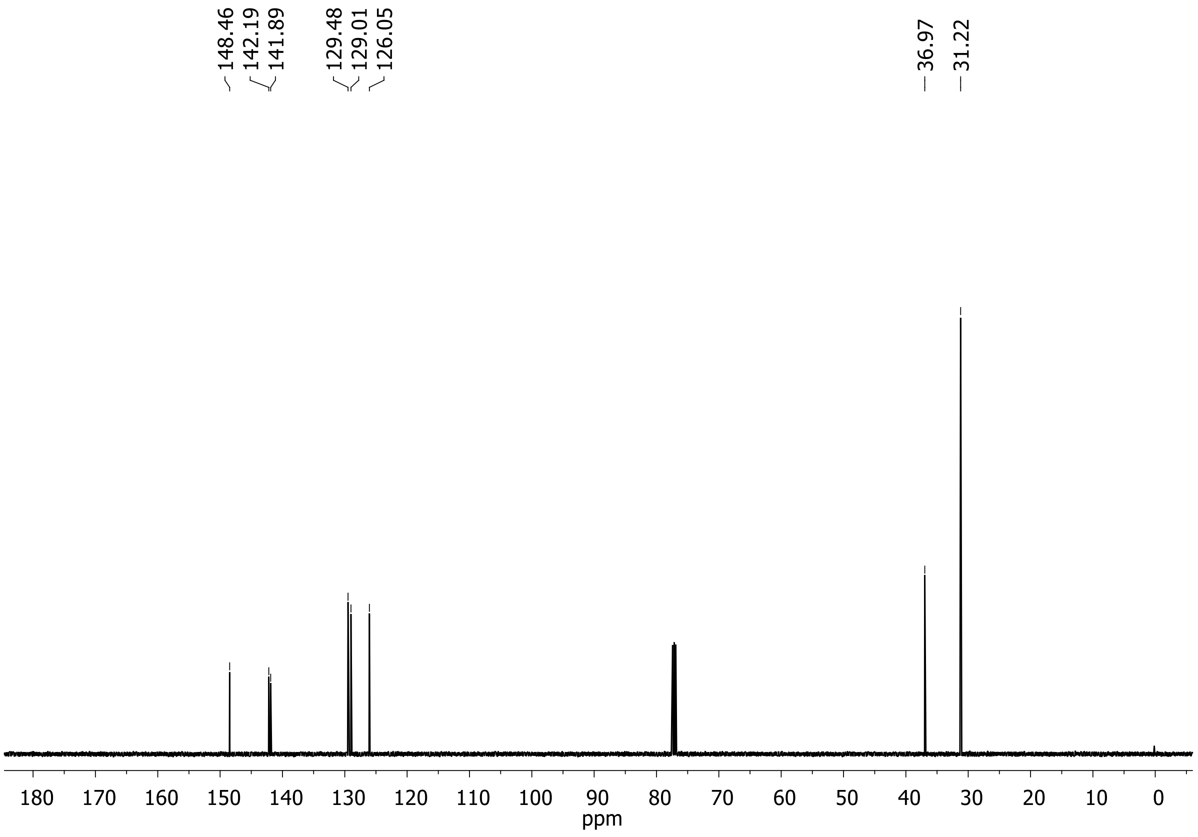

Figure SI6: $^{13}C\{^{1}H\}$ NMR spectrum of compound **16**.

2,2’-bis(Trimethylsilyl)azobenzene (**6**)

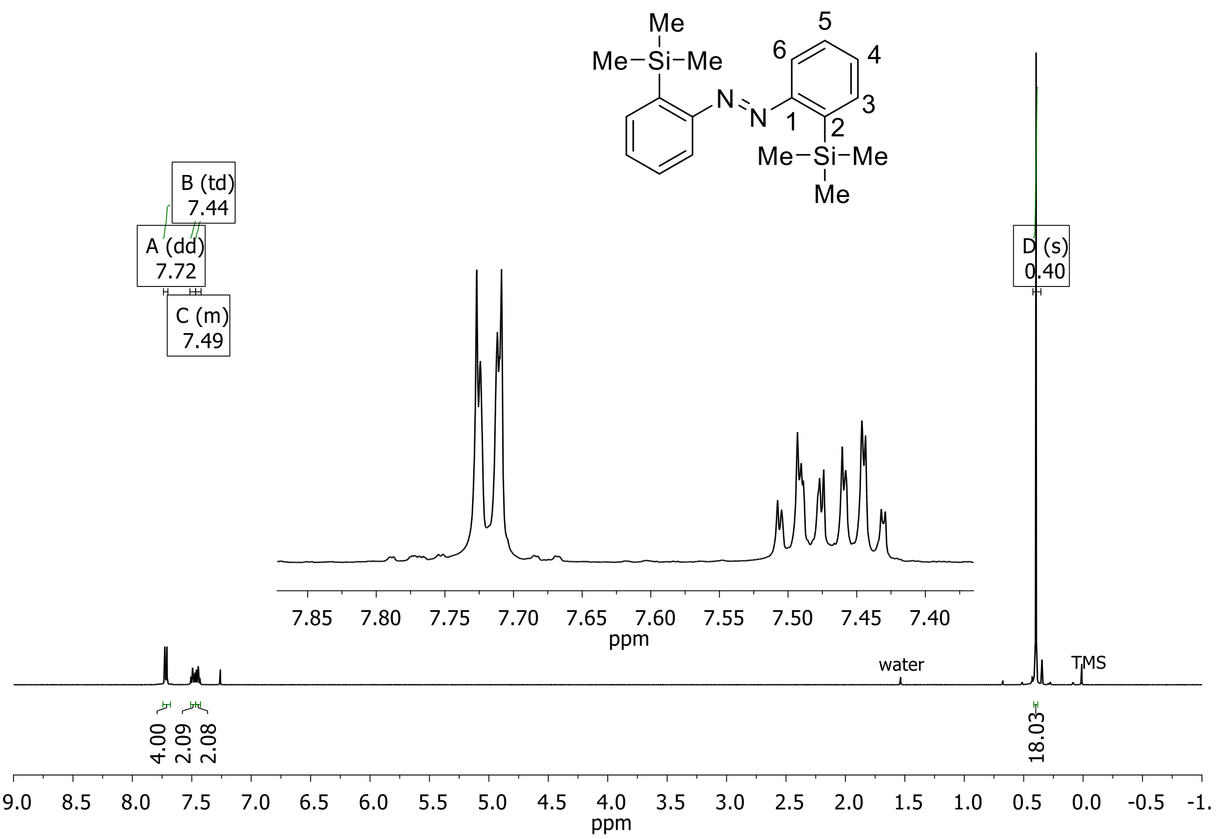

Figure SI7: ^{1}H NMR spectrum of compound **6**.

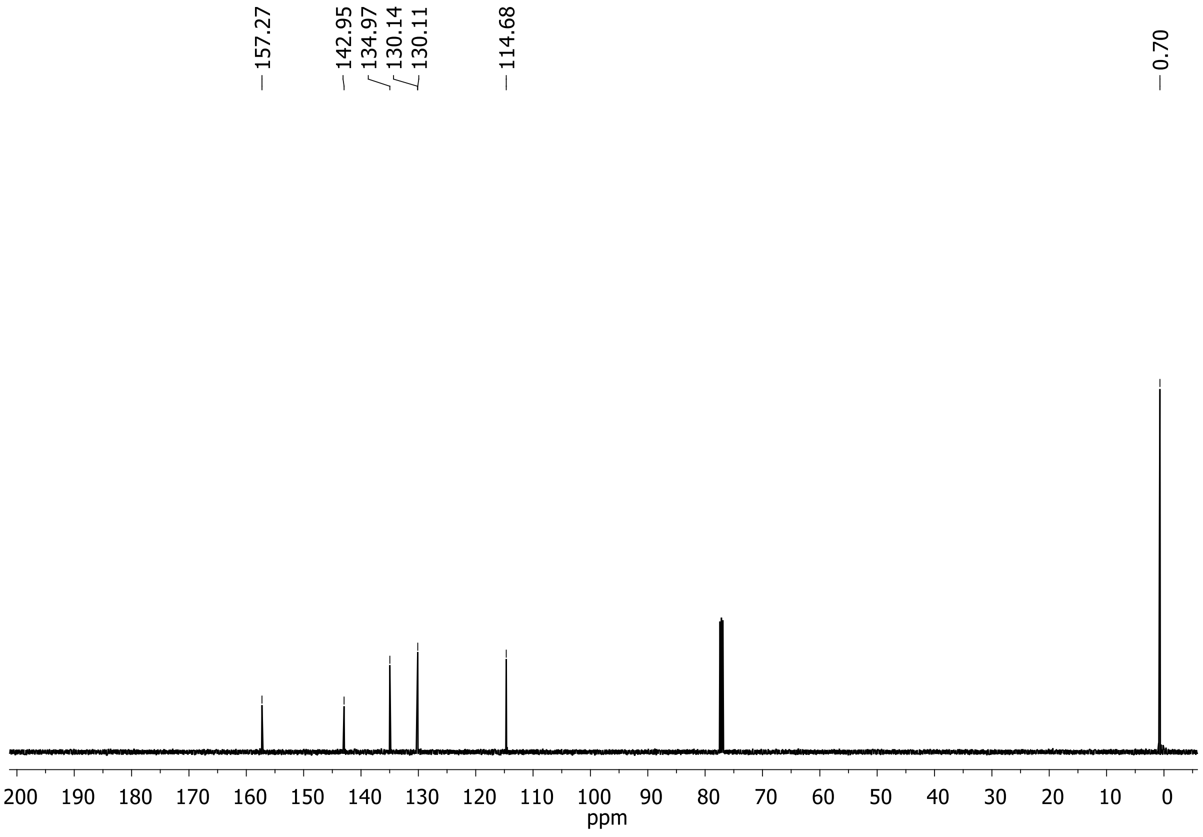

Figure SI8: ^{13}C{^{1}H} NMR spectrum of compound **6**.

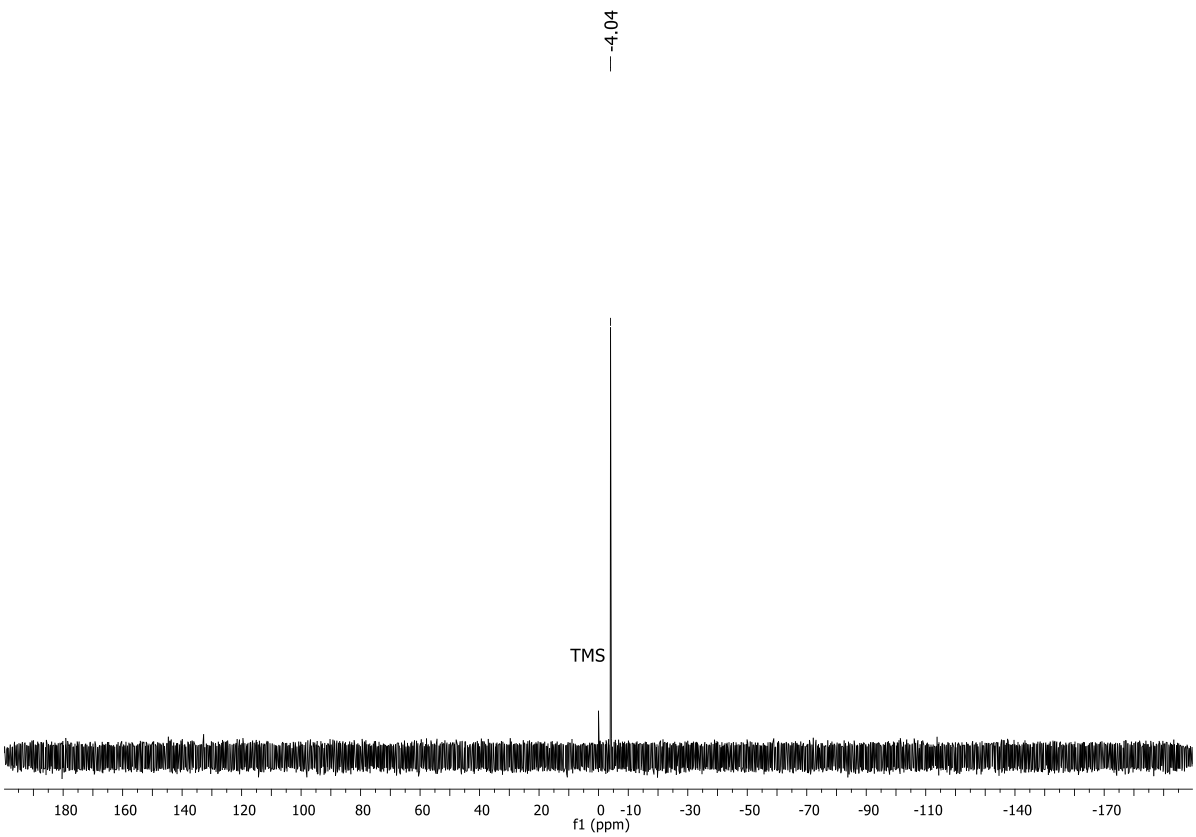

Figure SI9: $^{29}Si\{^{1}H\}$ NMR spectrum of compound **6**.

2,2'-bis(Trimethylgermanyl)azobenzene (**7**)

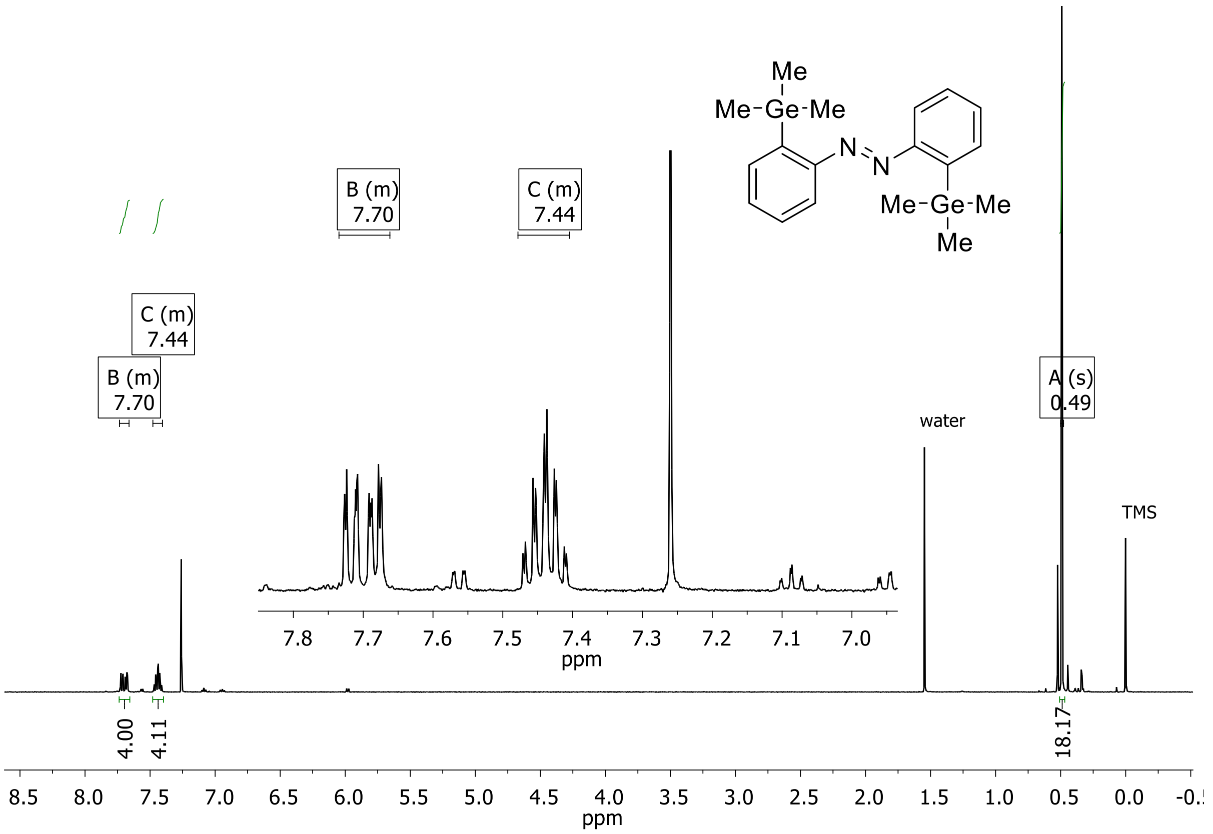

Figure SI10: ^{1}H NMR spectrum of compound **7**. The less intense signals in the aromatic region can be assigned to the *cis*-isomer.

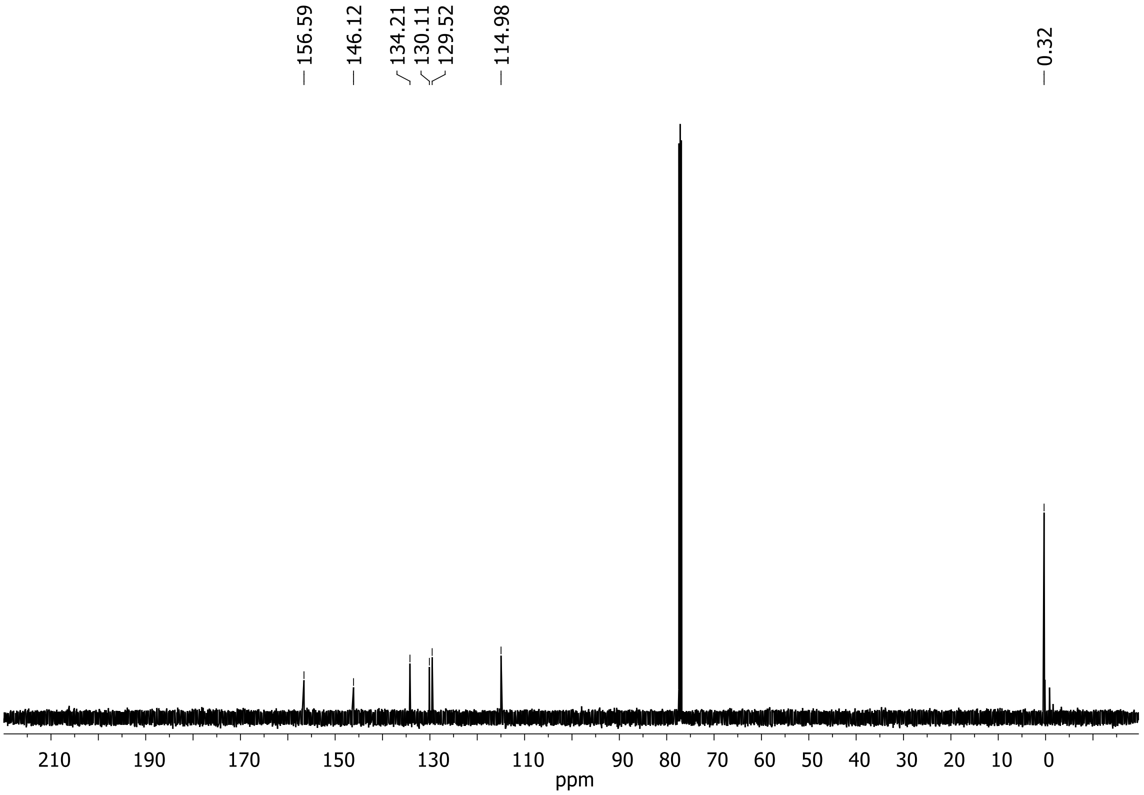

Figure SI11: $^{13}C\{^{1}H\}$ NMR spectrum of compound **7**.

2,2’-bis(Trimethylstanny)azobenzene (**8**)

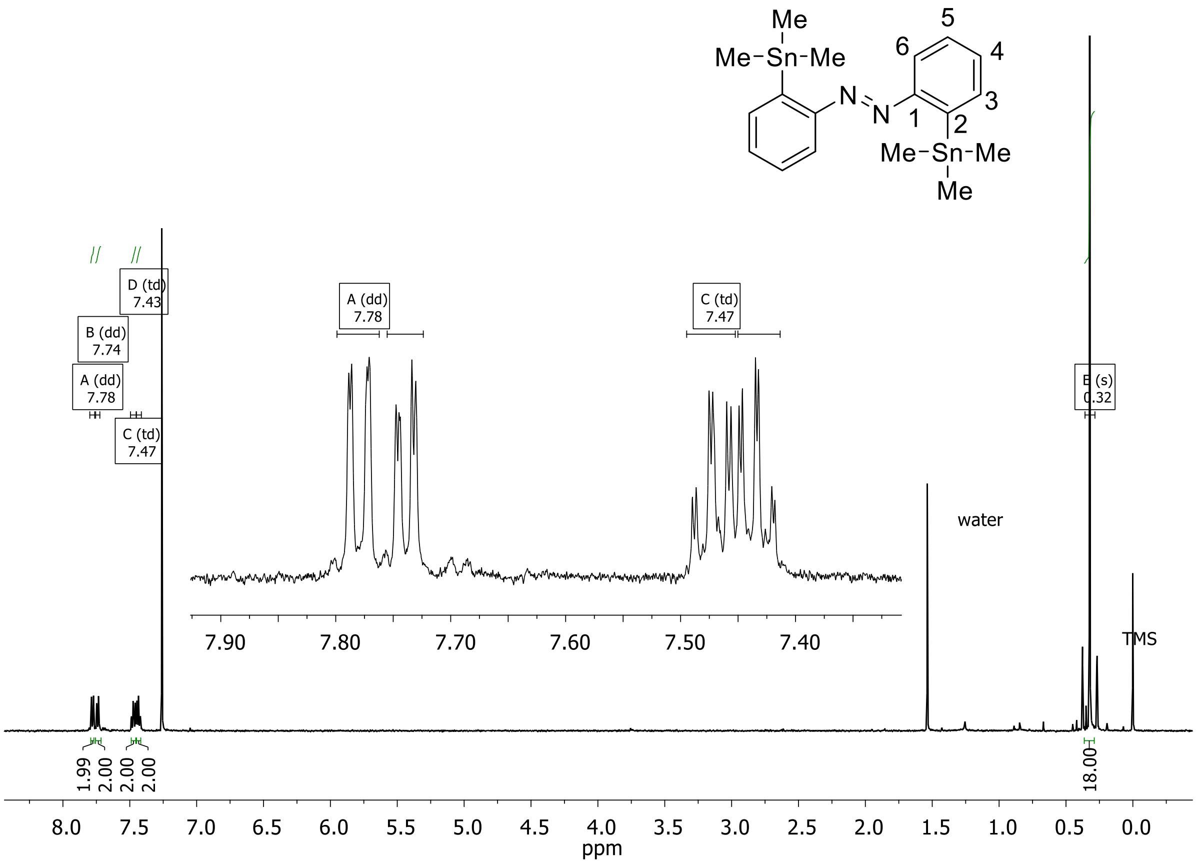

Figure SI12: ^{1}H NMR spectrum of compound **8**.

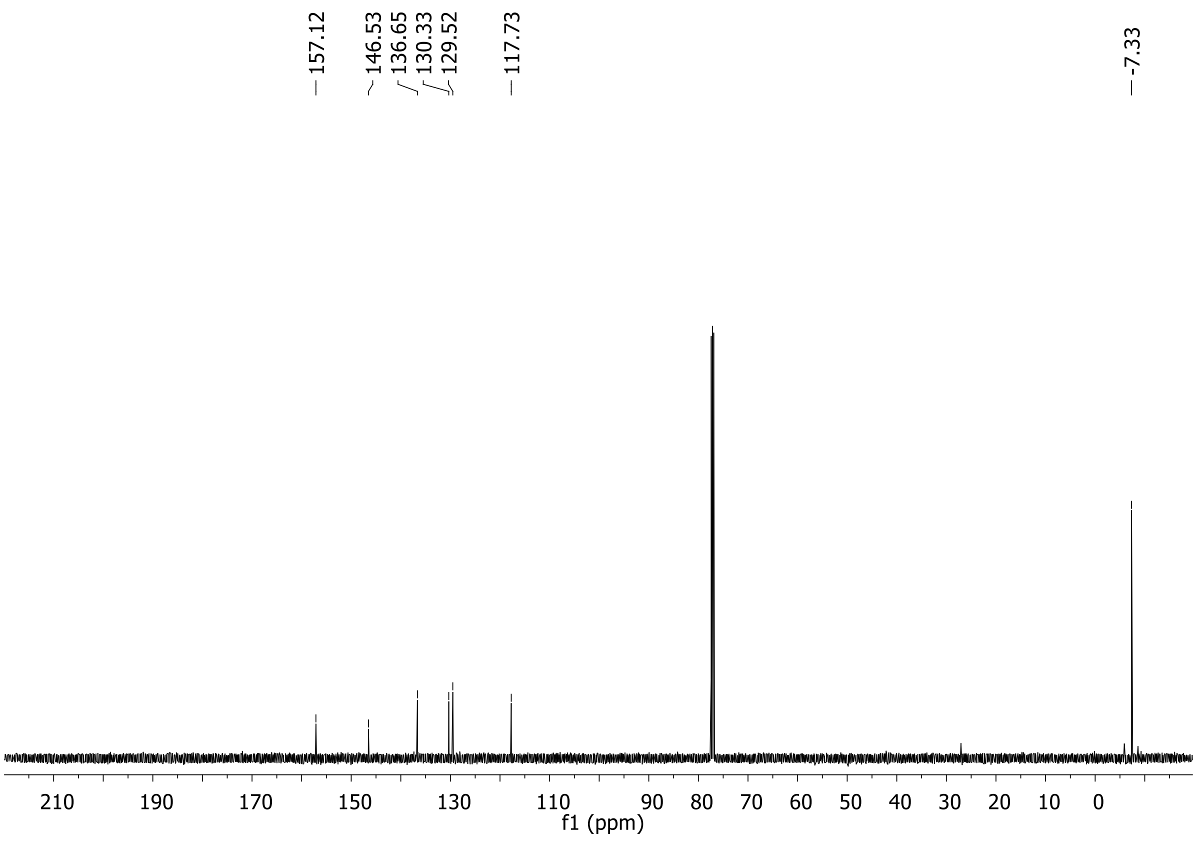

Figure SI13: $^{13}C\{^{1}H\}$ NMR spectrum of compound 8.

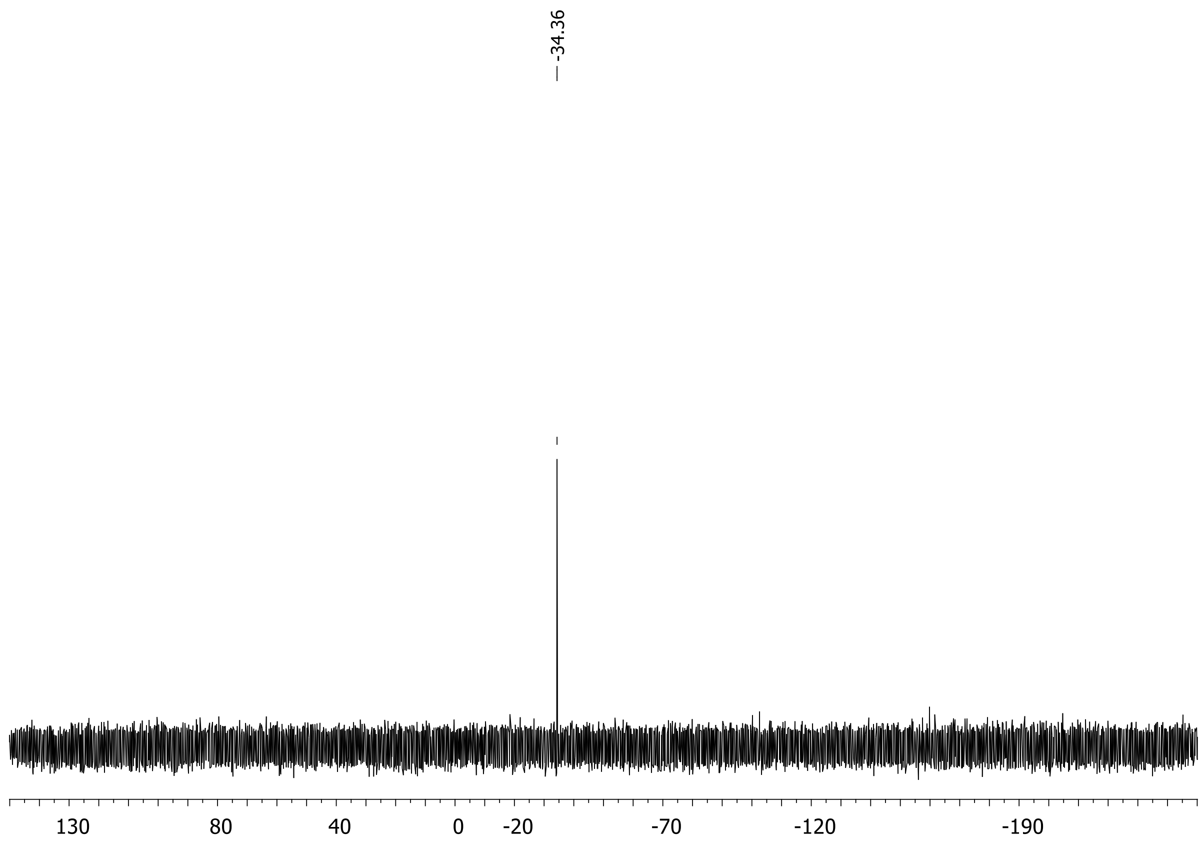

Figure SI14: $^{119}Sn\{^1H\}$ NMR spectrum of compound **8**.

4. UV/Vis Spectra and ^{1}H NMR Spectra of the Switching Experiments

The solutions for the UV-vis spectra were prepared using a stock solution with the appropriate amount of compound in cyclohexane and diluting it to obtain different concentrations.

For the switching experiments we used the maximum of the ππ*-band and not a absorption value at a fixed wavelength, since the position of the absorption maximum shifted slightly during the switching process.

2,2'-Di(*tert*-butyl)azobenzene (**10**)

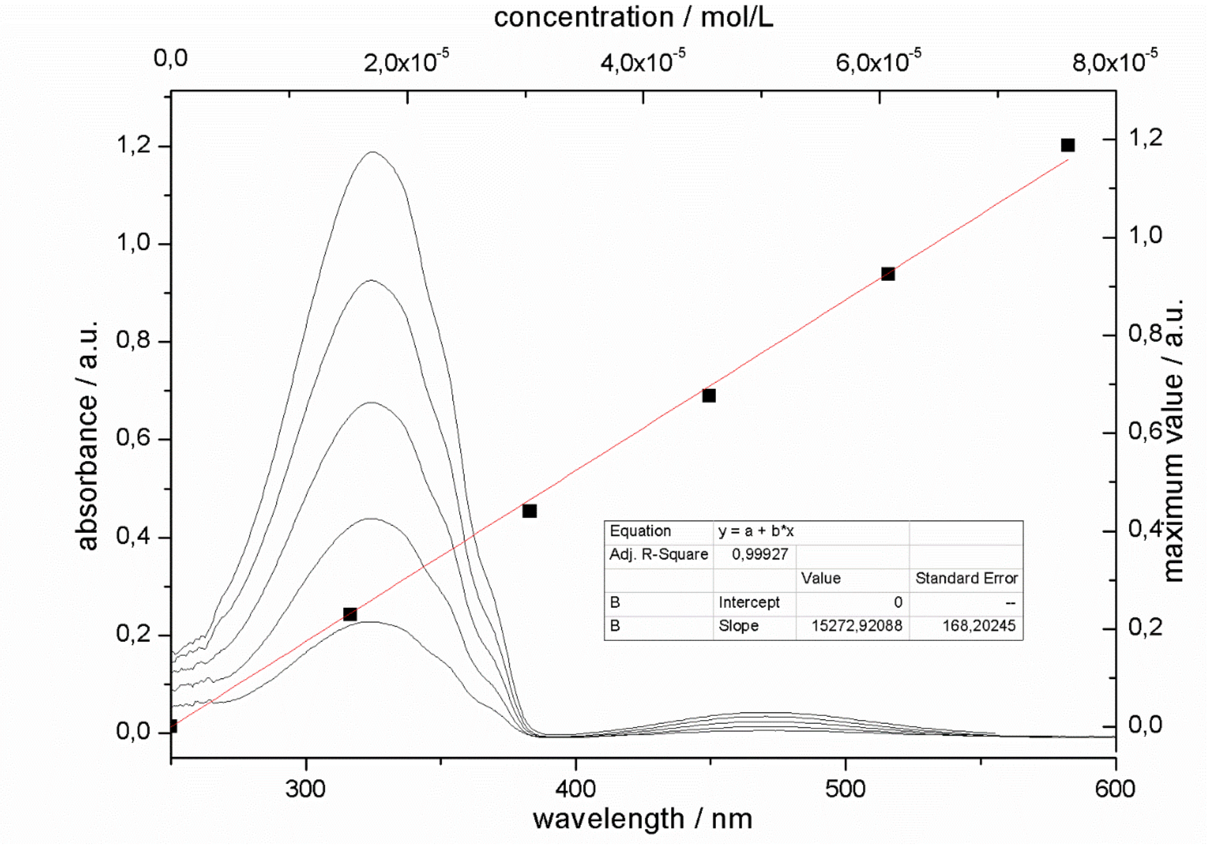

Figure SI15: Absorption spectra of compound **10** and linear fitting to according to Lambert-Beer's-law.

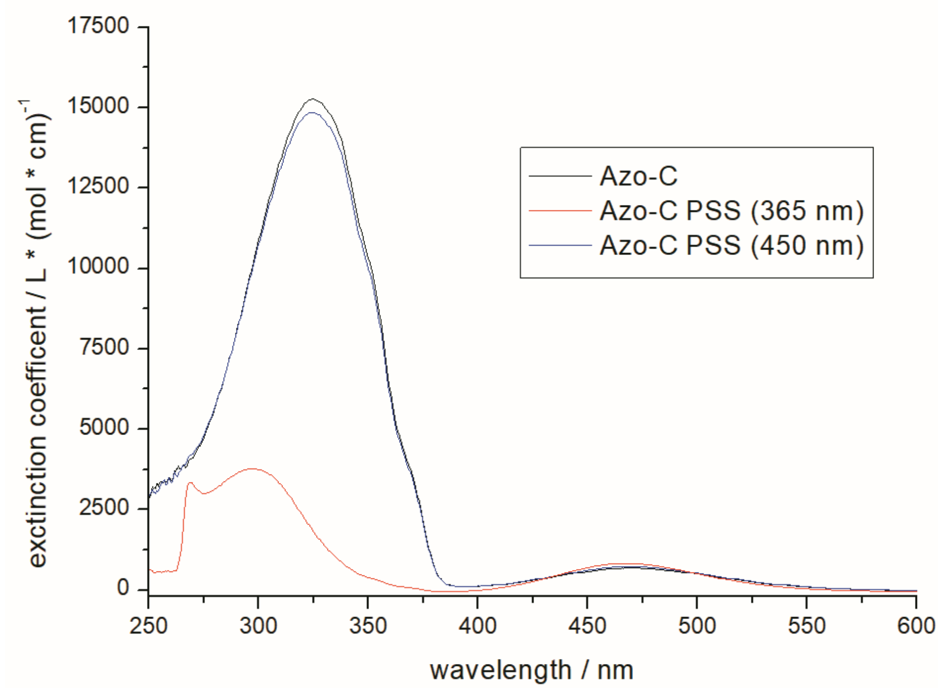

Figure SI16: Comparison of the absorption spectra of compound **10** dissolved as synthesized (black), after 3 min of irradiation with 365 nm light (red) and after 15 min of irradiation with 450 nm light (blue). The concentration was 4.56 x 10^{-5} M in cyclohexane.

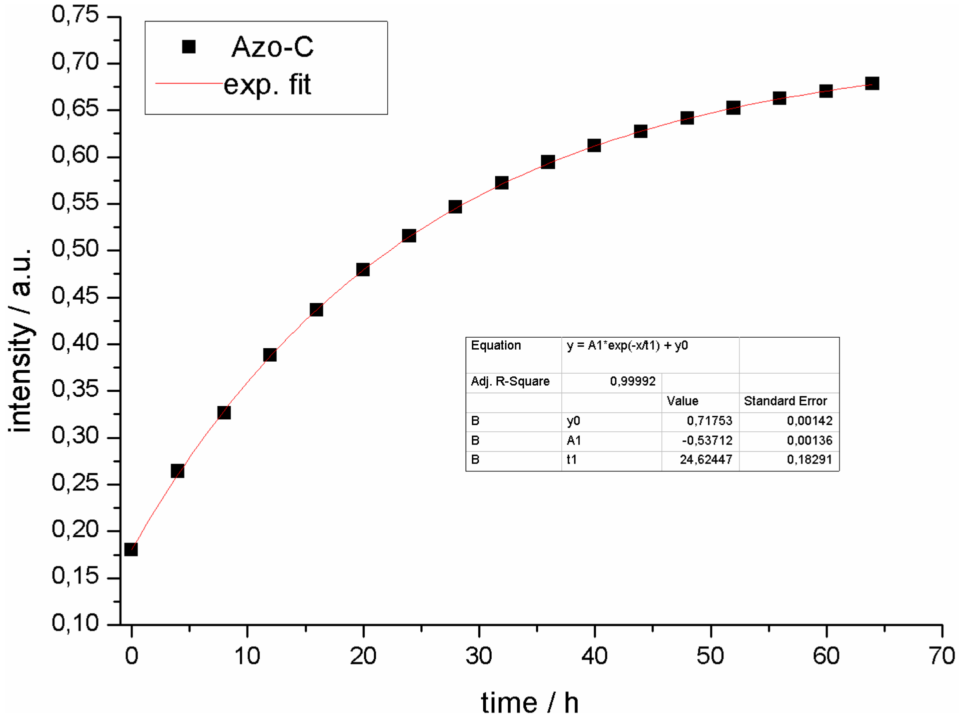

Figure SI17: Absorption value of the ππ* band from compound **10** plotted against the time after irradiation at 365 nm for 3 mins. The concentration was 4.56 x 10^{-5} M in cyclohexane.

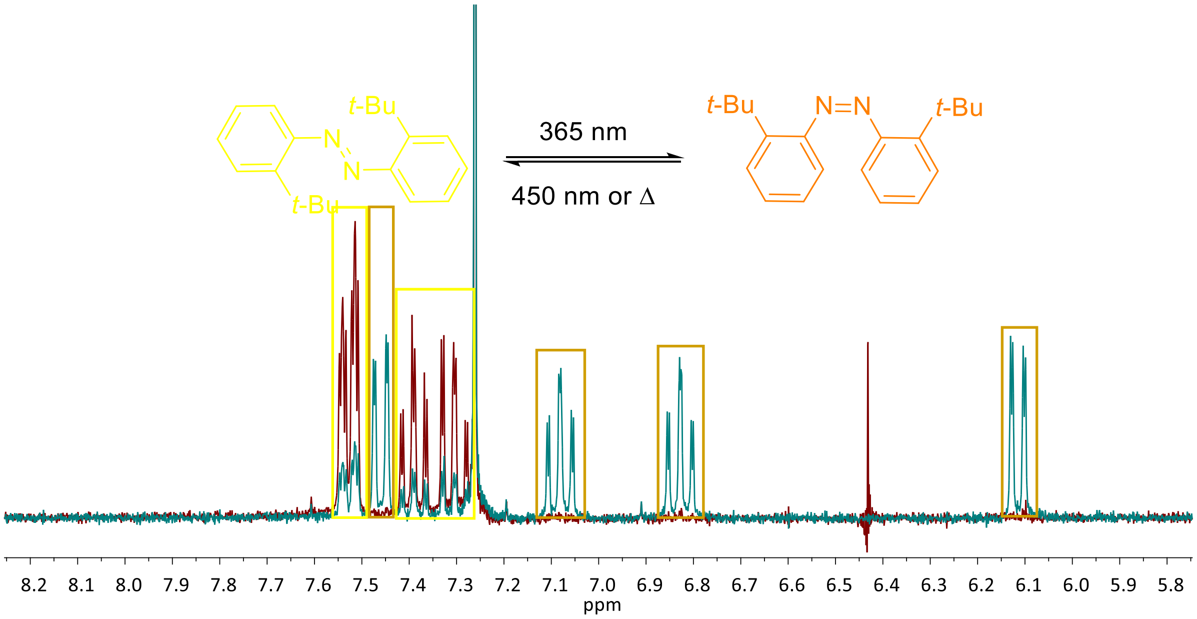

Figure SI18: ^{1}H NMR (300 MHz) spectra of compound **10** before (red) and after irradiation (blue) with 365 nm (15 min) in $CDCl_3$. The concentration was 4.84 x 10^{-3} M.

^{1}H NMR spectroscopic monitoring of the switching of compound **10:**

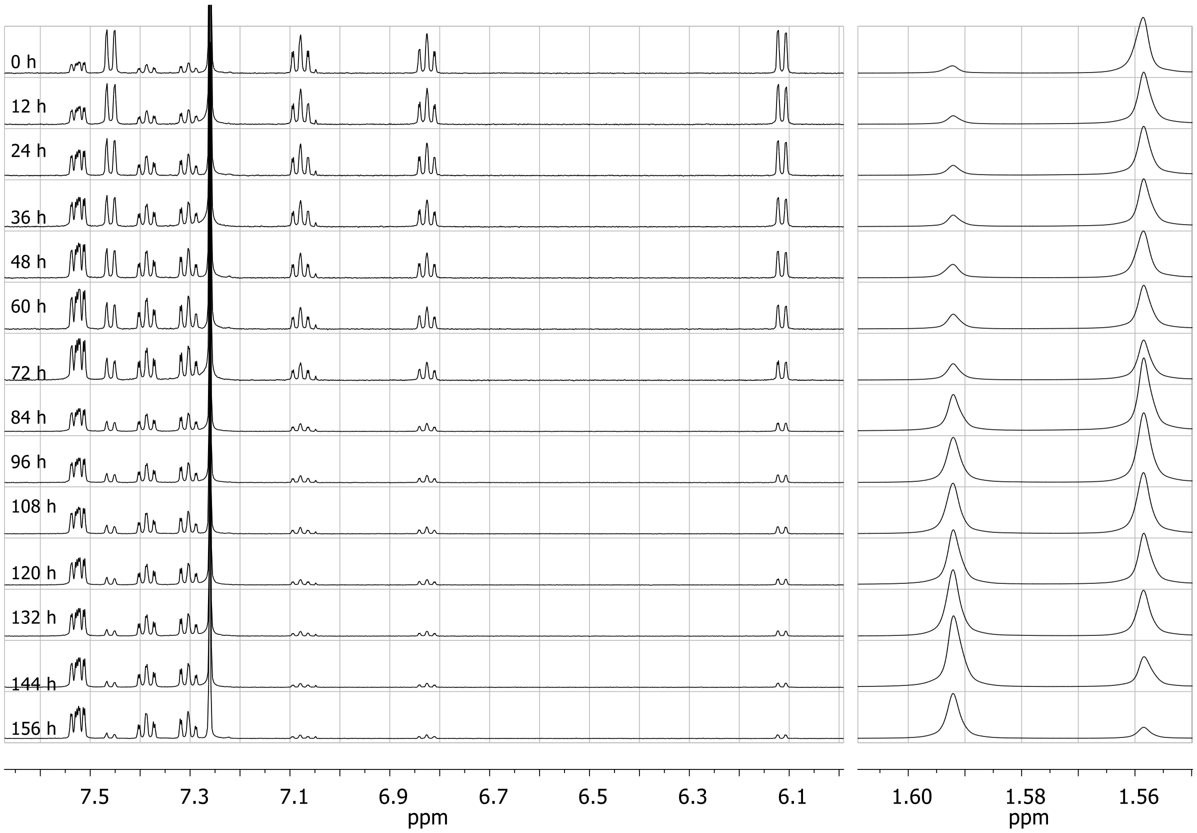

Figure SI19: ^{1}H NMR (500 MHz) spectra of **10** in $CDCl_3$ measured every 12 h after irradiation at 365 nm for 15 mins.

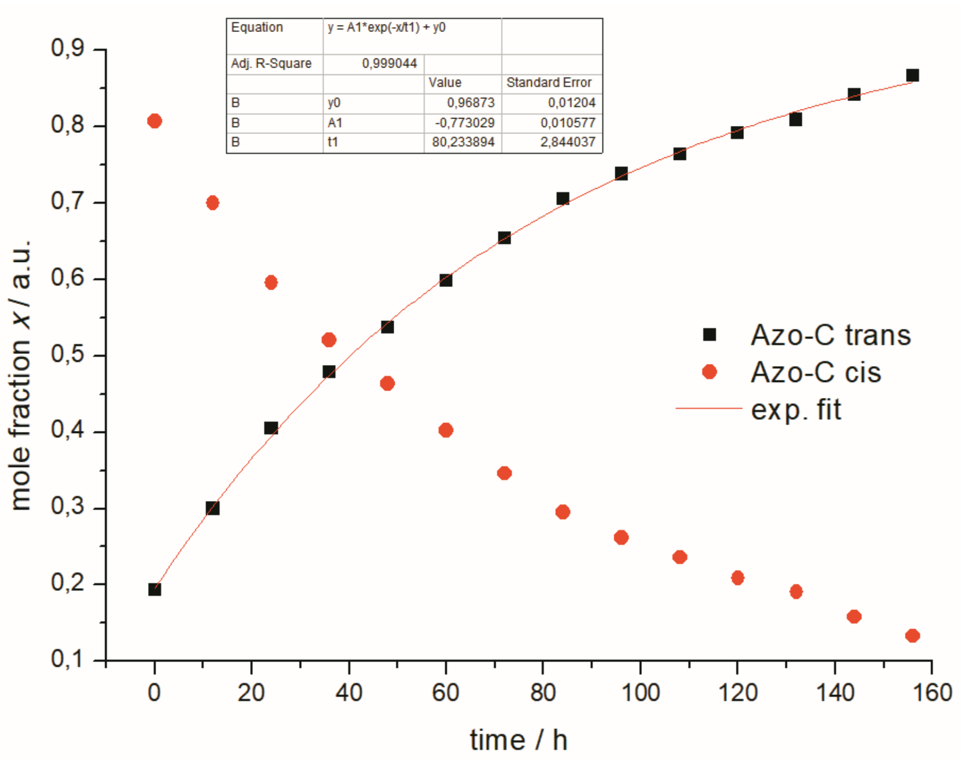

Figure SI20: Mole fraction of *cis* and *trans*-azobenzene **10** vs. time gives the thermal relaxation at 300 K.

2,2’-bis(Trimethylsilyl)azobenzene (**6**)

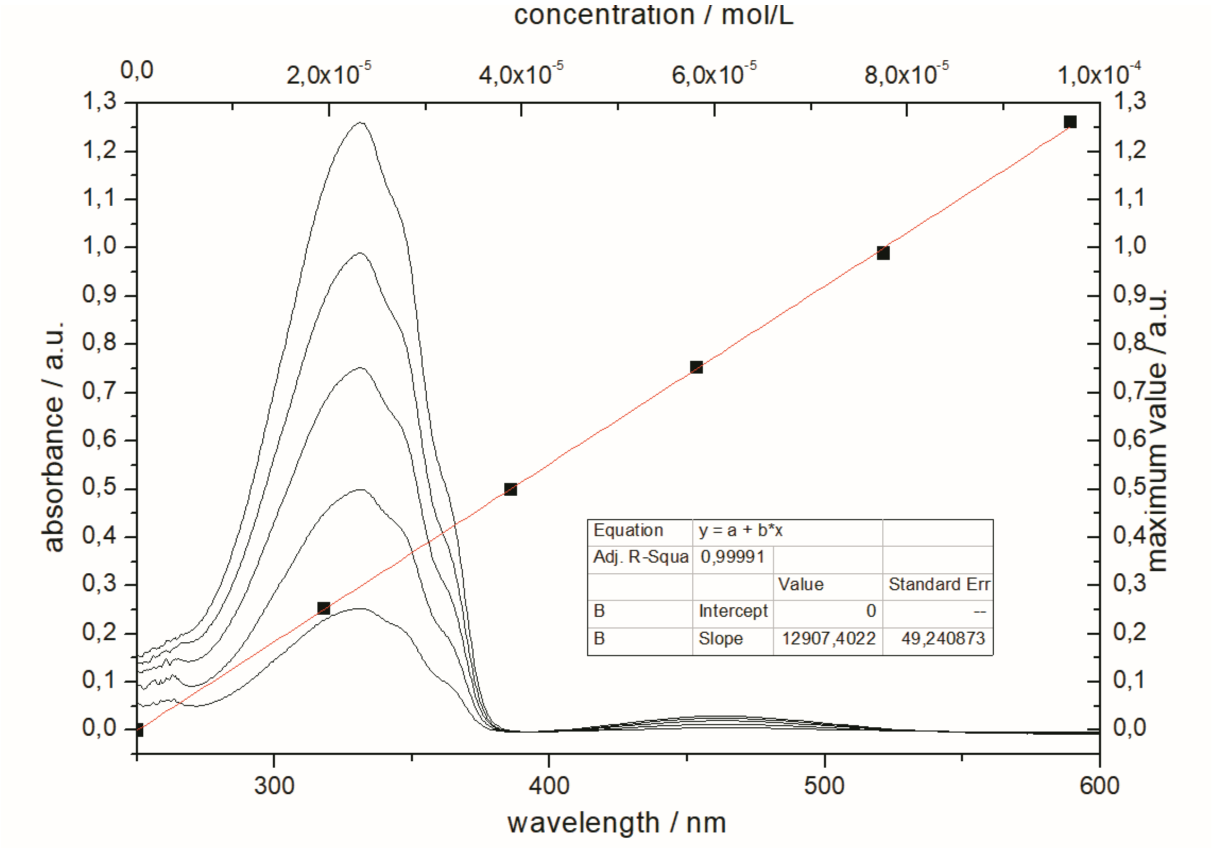

Figure SI21: Absorption spectra of compound **6** and linear fitting to according to Lambert-Beer’s-law.

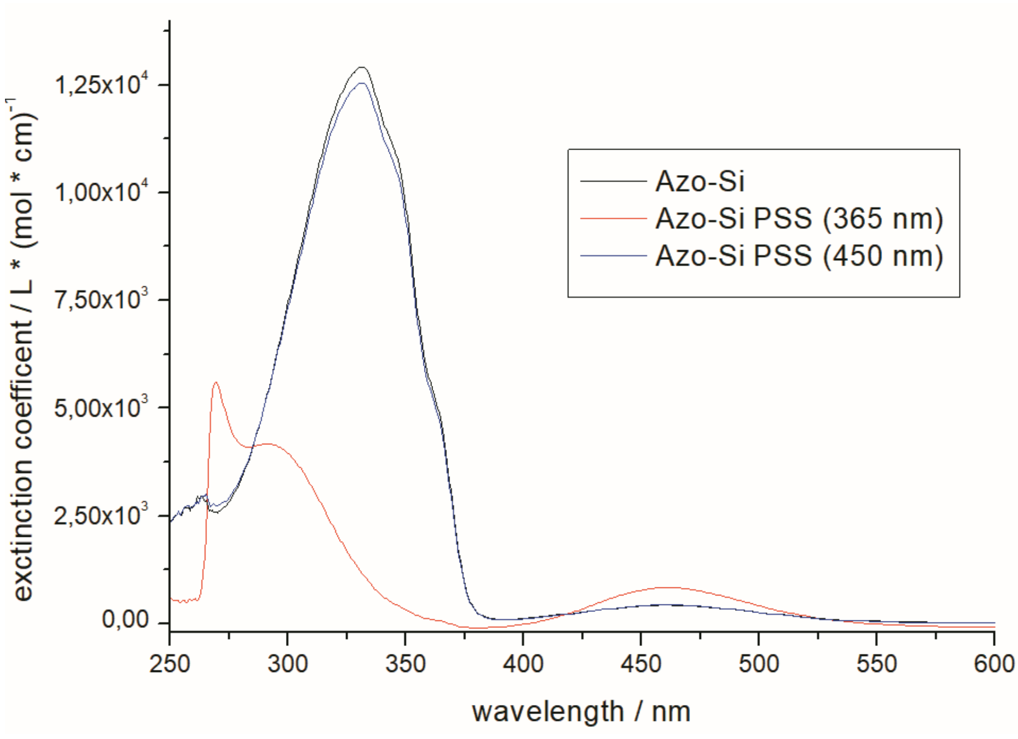

Figure SI 22: Comparison of the absorption spectra of compound **6** as dissolved (black), after 3 min irradiation with 365 nm light (red) and after 15 min irradiation with 450 nm light (blue). The concentration was 9.70 x 10^{-5} M in cyclohexane.

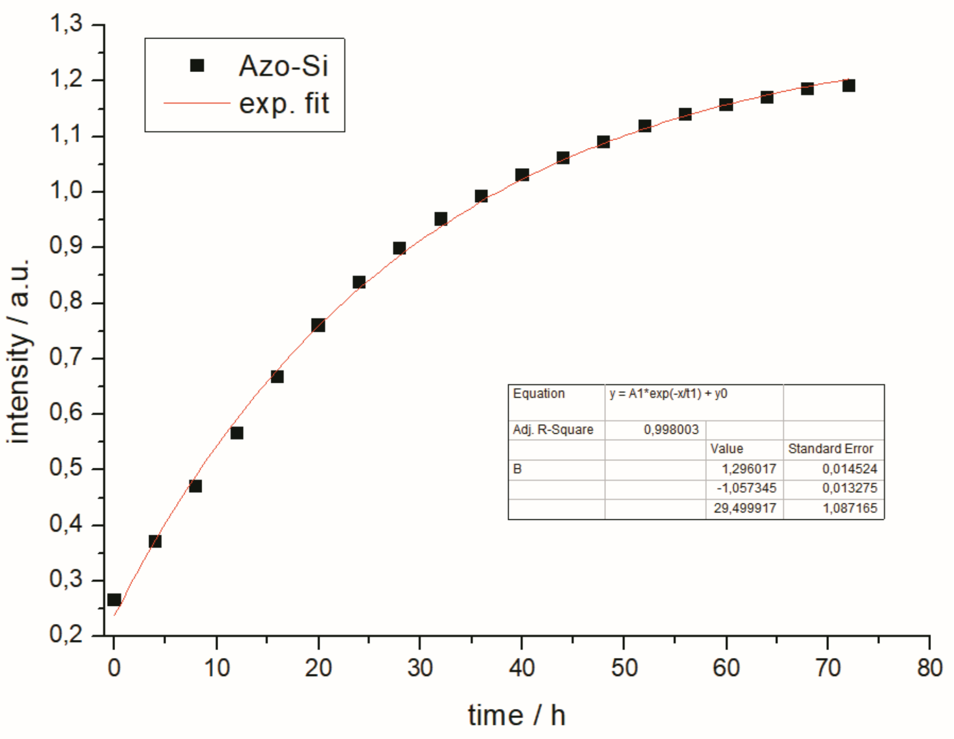

Figure SI23: Absorption value of the ππ* band from compound **6** plotted against the time after irradiation at 365 nm for 3 mins. The concentration was 9.70 x 10^{-5} M in cyclohexane.

^{1}H NMR spectroscopic monitoring of the switching of **6**:

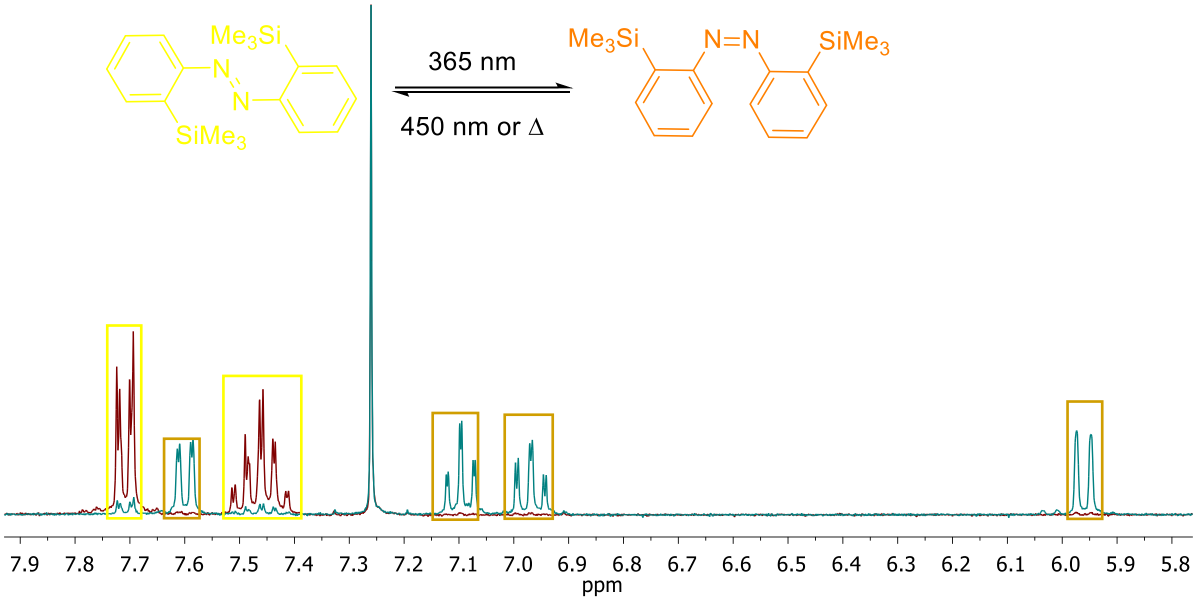

Figure SI24: ^{1}H NMR (300 MHz) spectra of compound **6** before (red) and after (blue) irradiation with 365 nm (15 min) in $CDCl_3$. The concentration was 1.07 *x* 10^{-2} M.

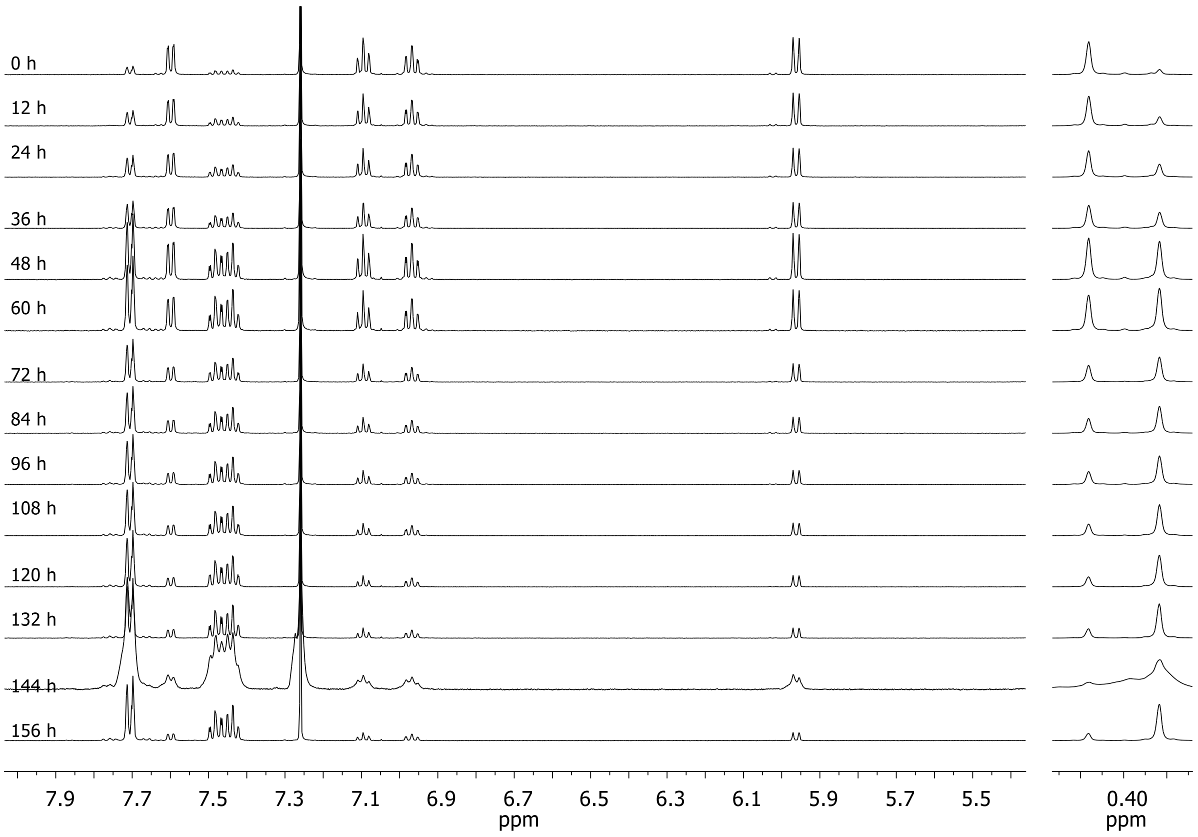

Figure SI25: ^{1}H NMR (500 MHz) spectra of **6** in $CDCl_3$ measured every 12 h after irradiation at 365 nm for 15 min. The second to last NMR spectrum failed to shim but integrals could be determined.

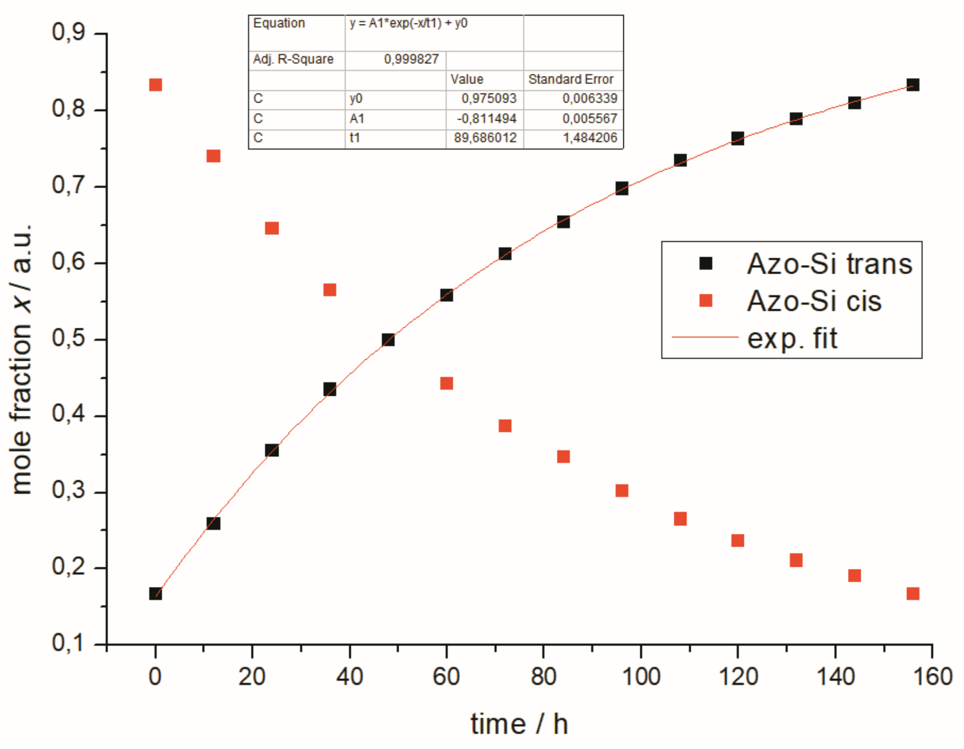

Figure SI26: Mole fraction of cis and trans-azobenzene **6** vs. time gives the thermal relaxation at 300 K.

2,2'-bis(Trimethylgermanyl)azobenzene (**7**)

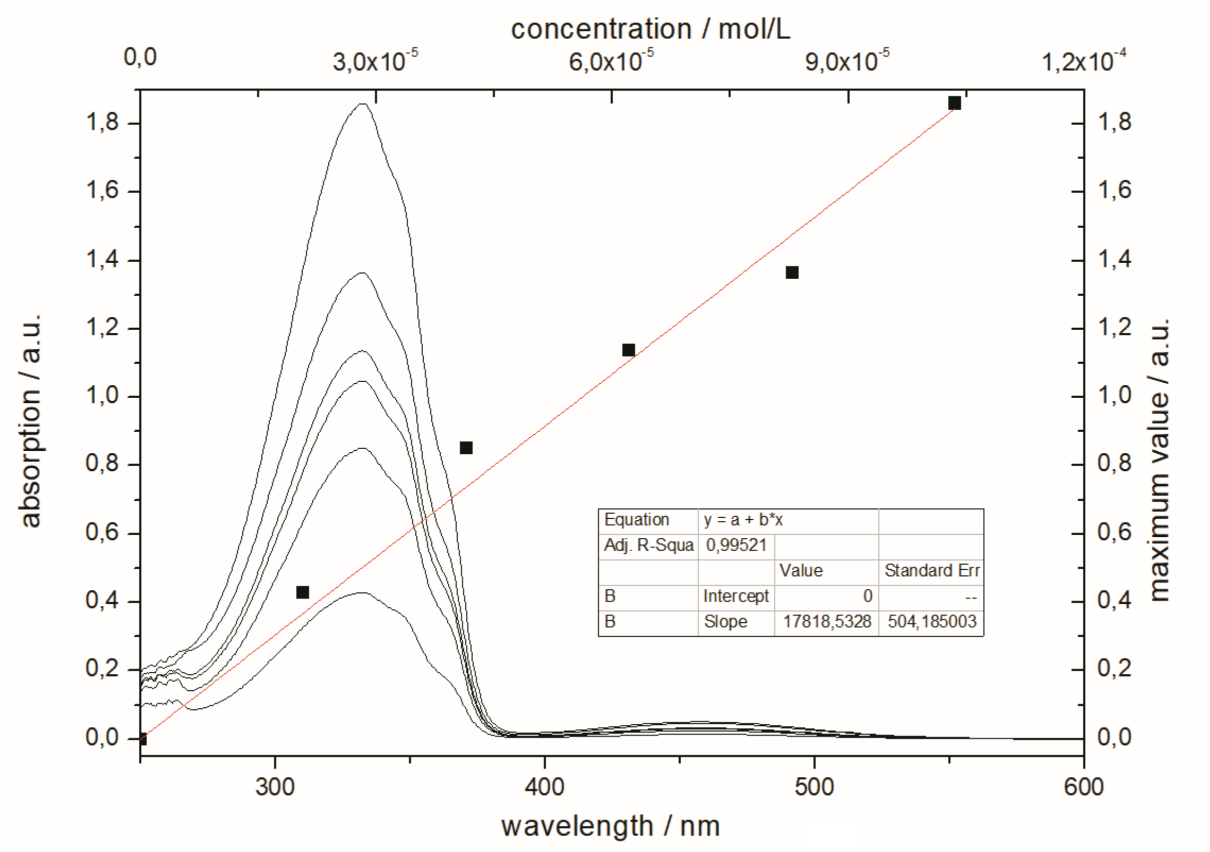

Figure SI27: Absorption spectra of compound **7** and linear fitting to according to Lambert-Beer's-law.

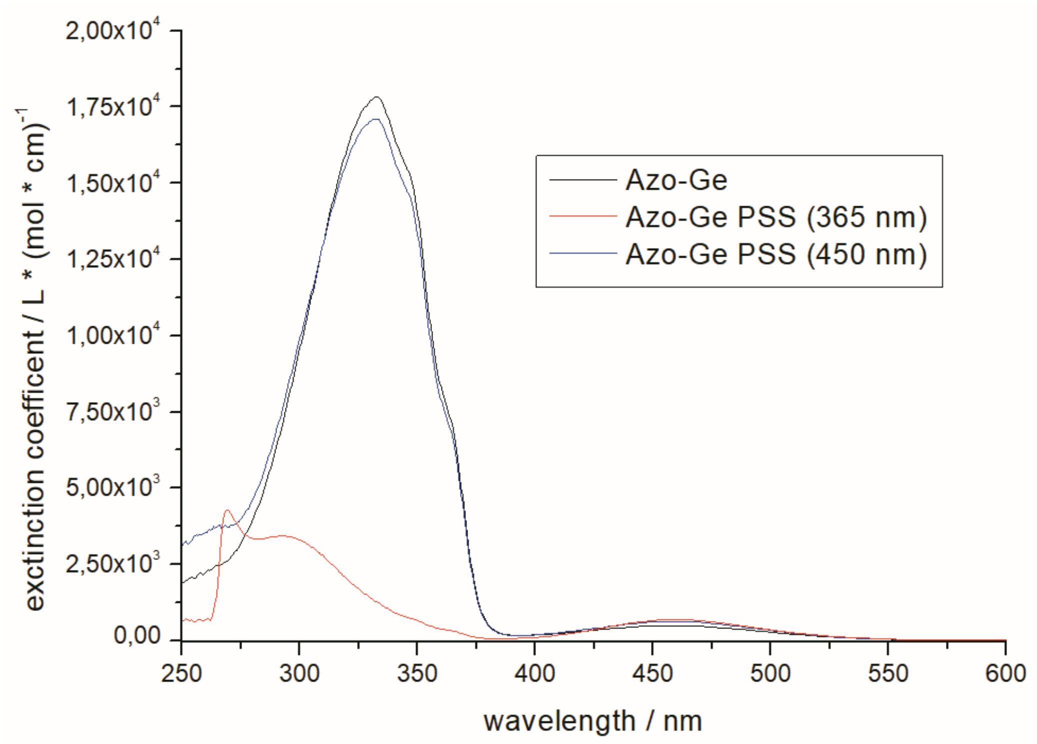

Figure SI28: Comparison of the absorption spectra of compound **7** as dissolved (black), after 3 min irradiation with 365 nm light (red) and after 15 min irradiation with 450 nm light (blue). The concentration was 6.22 x 10^{-5} M in cyclohexane.

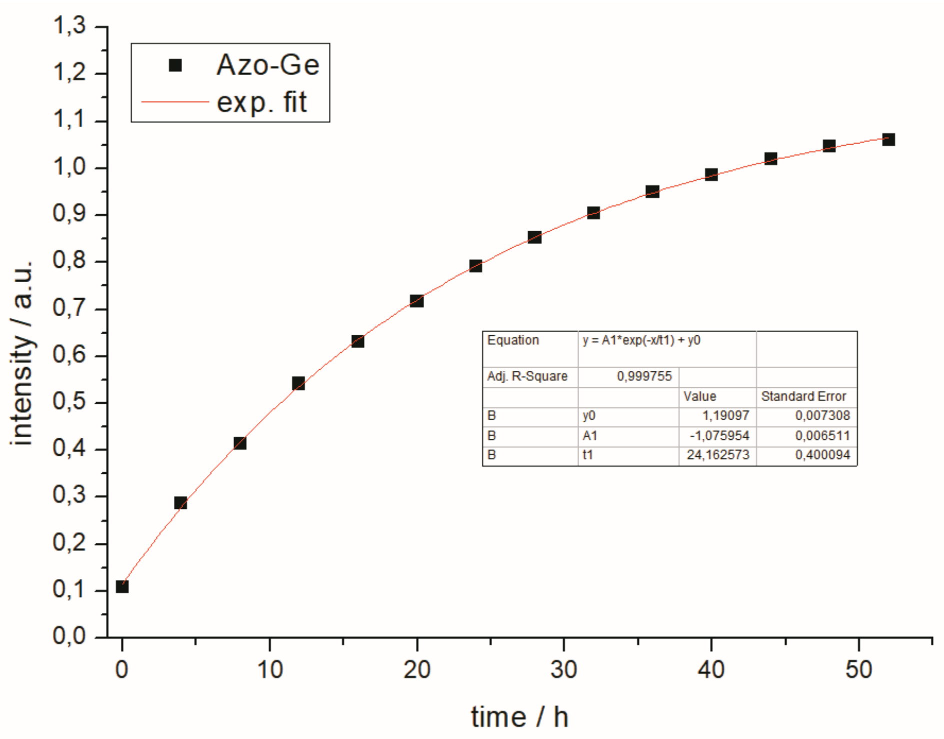

Figure SI29: Absorption value of the ππ* band from compound **7** plotted against time after irradiation at 365 nm for 3 mins. The concentration was 6.22 x 10^{-5} M in cyclohexane.

^{1}H NMR spectroscopic monitoring of the switching of **7**:

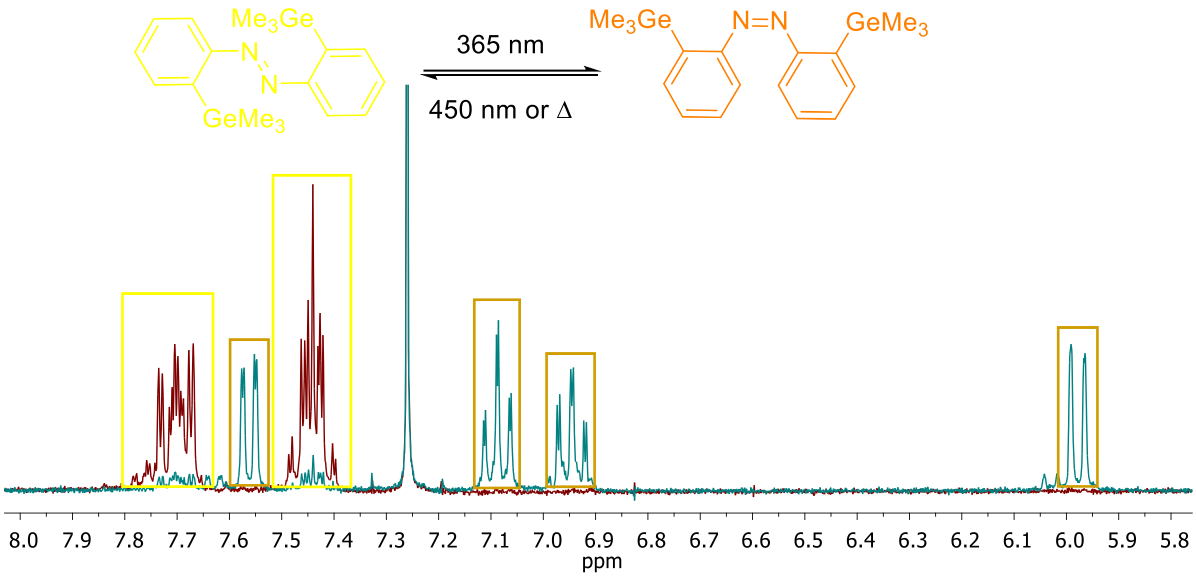

Figure SI 30: ^{1}H NMR (300 MHz) spectra of compound **7** in $CDCl_3$ before (red) and after (blue) irradiation with 365 nm (15 min) in $CDCl_3$. The concentration was 7.53 x 10^{-3} M.

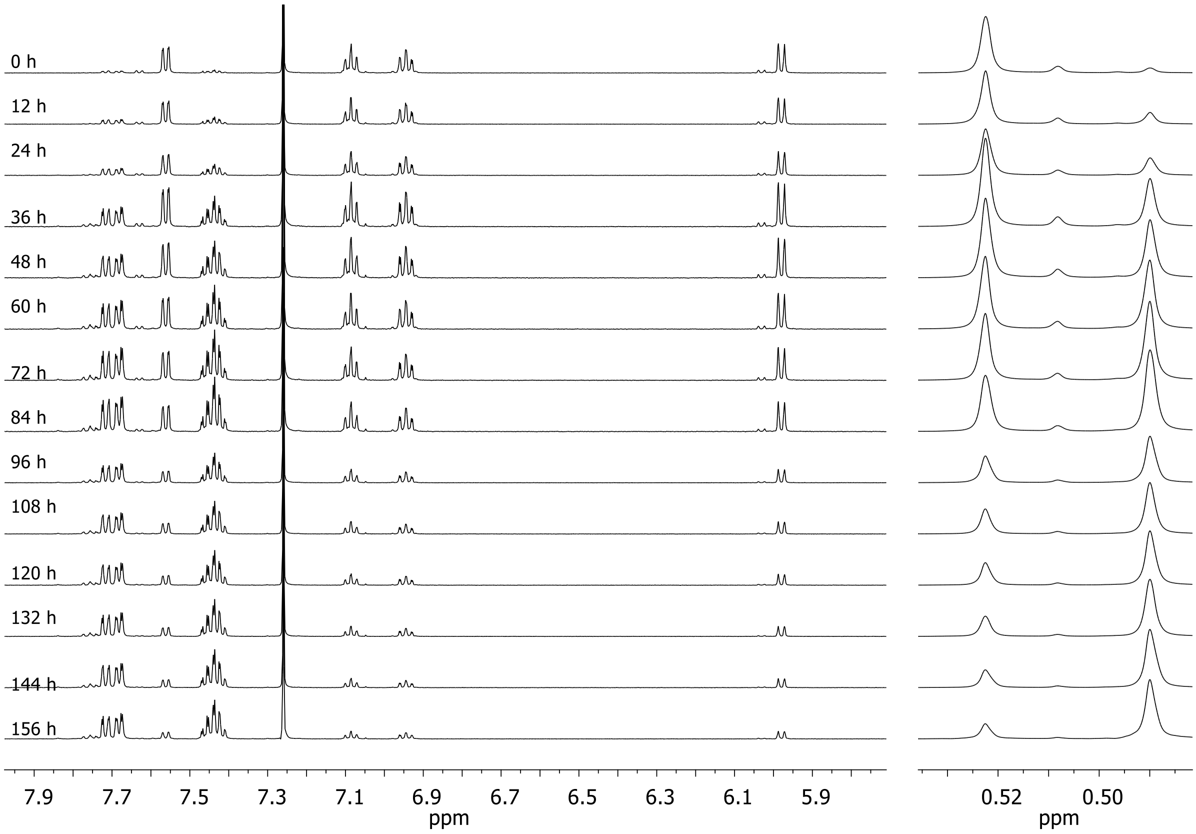

Figure SI31: ^{1}H NMR (500 MHz) spectra of **7** measured every 12 h after irradiation at 365 nm for 15 mins.

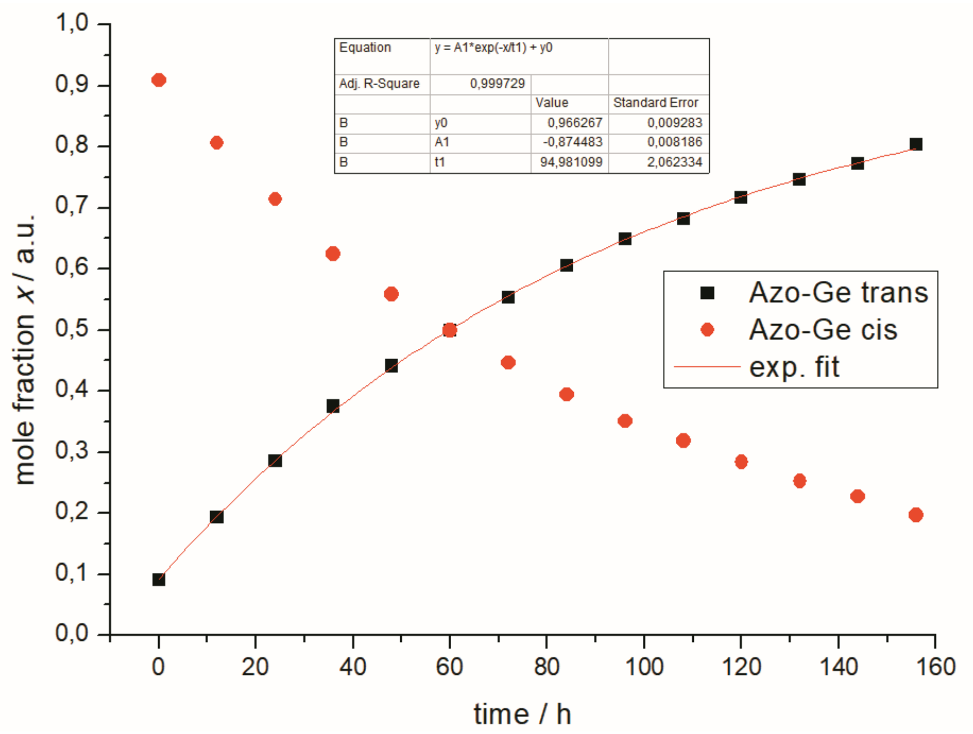

Figure SI32: Mole fraction of cis and trans-azobenzene **7** vs time gives the thermal relaxation at 300 K.

2,2'-bis(Trimethyltin)azobenzene (**8**)

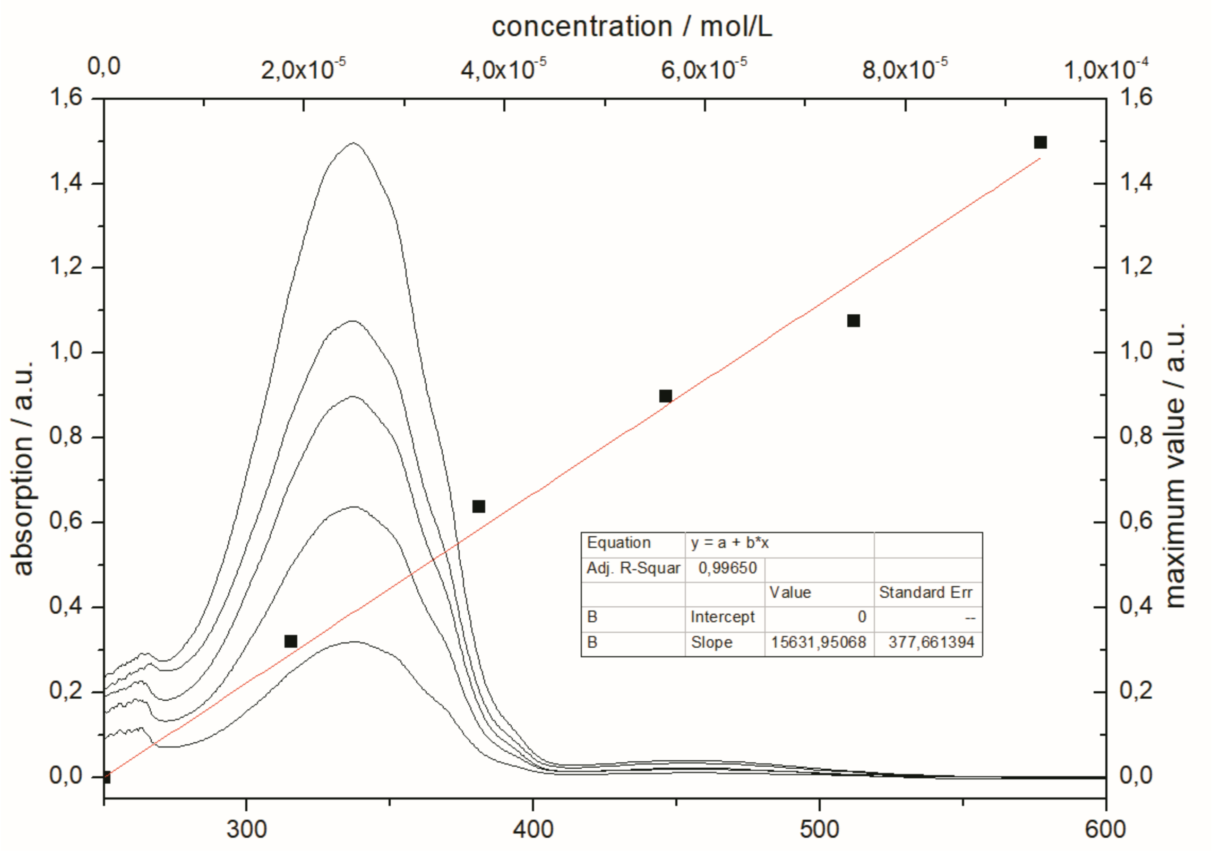

Figure SI33: Absorption spectra of compound **8** and linear fitting to according to Lambert-Beer's-law.

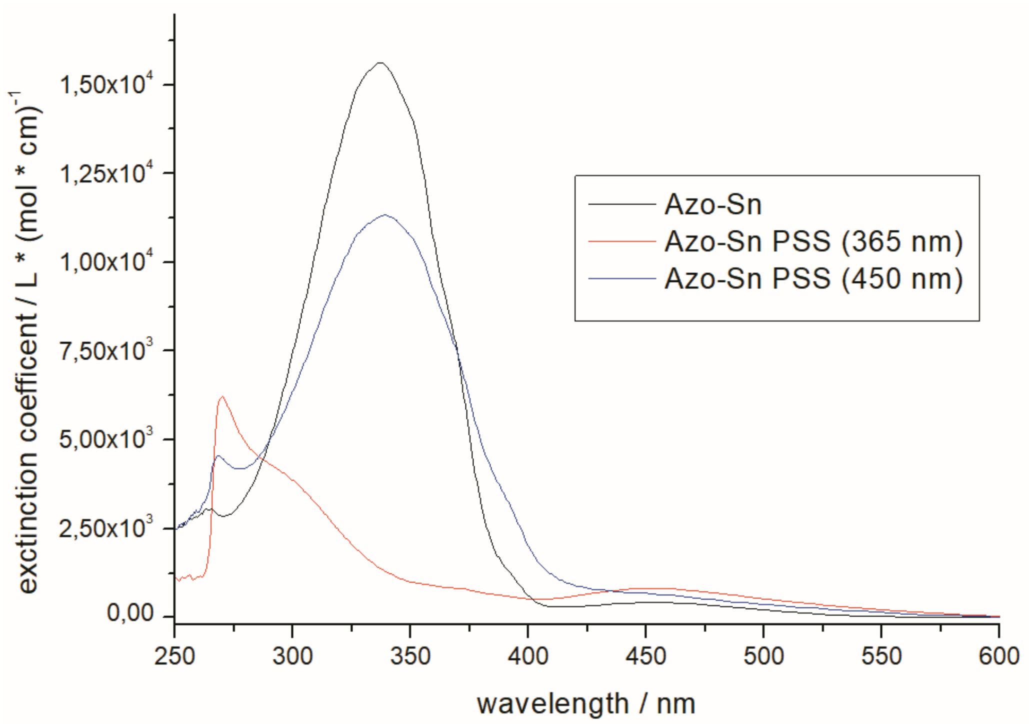

Figure SI34: Comparison of the absorption spectra of compound **8** as dissolved (black), after 3 min irradiation with 365 nm light (red) and after 15 min irradiation with 450 nm light (blue). The concentration was 5.60 x 10^{-5} M in cyclohexane.

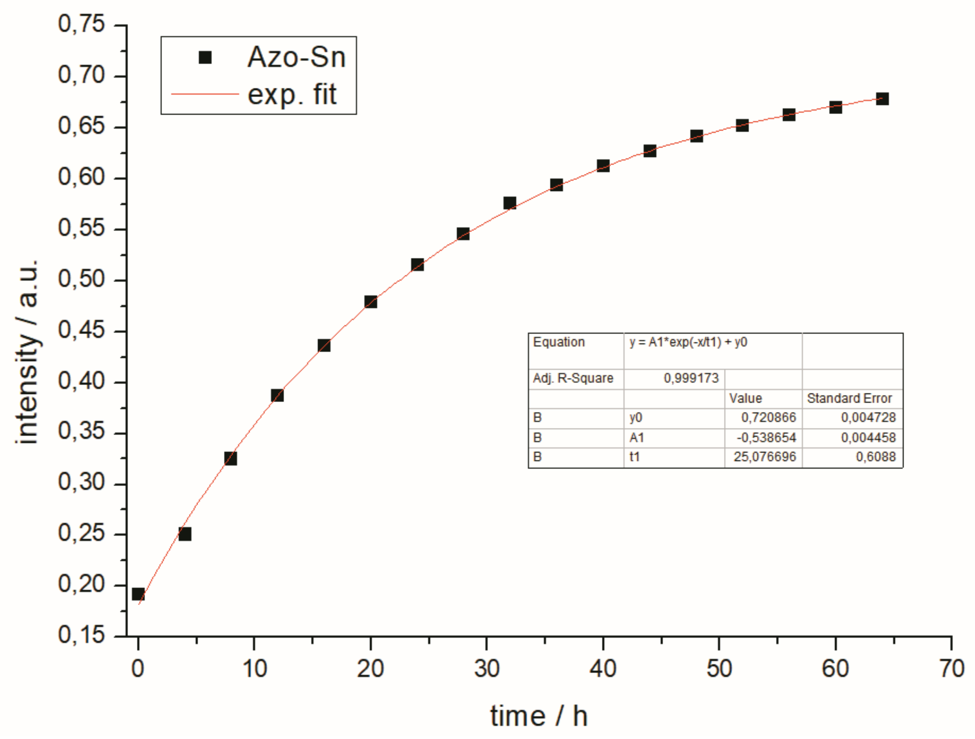

Figure SI35: Absorption value of the ππ* band from compound **8** plotted against the time after irradiation at 365 nm for 3 min. The concentration was 5.60 x 10^{-5} M in cyclohexane.

^{1}H NMR spectroscopic monitoring of the switching of **8**:

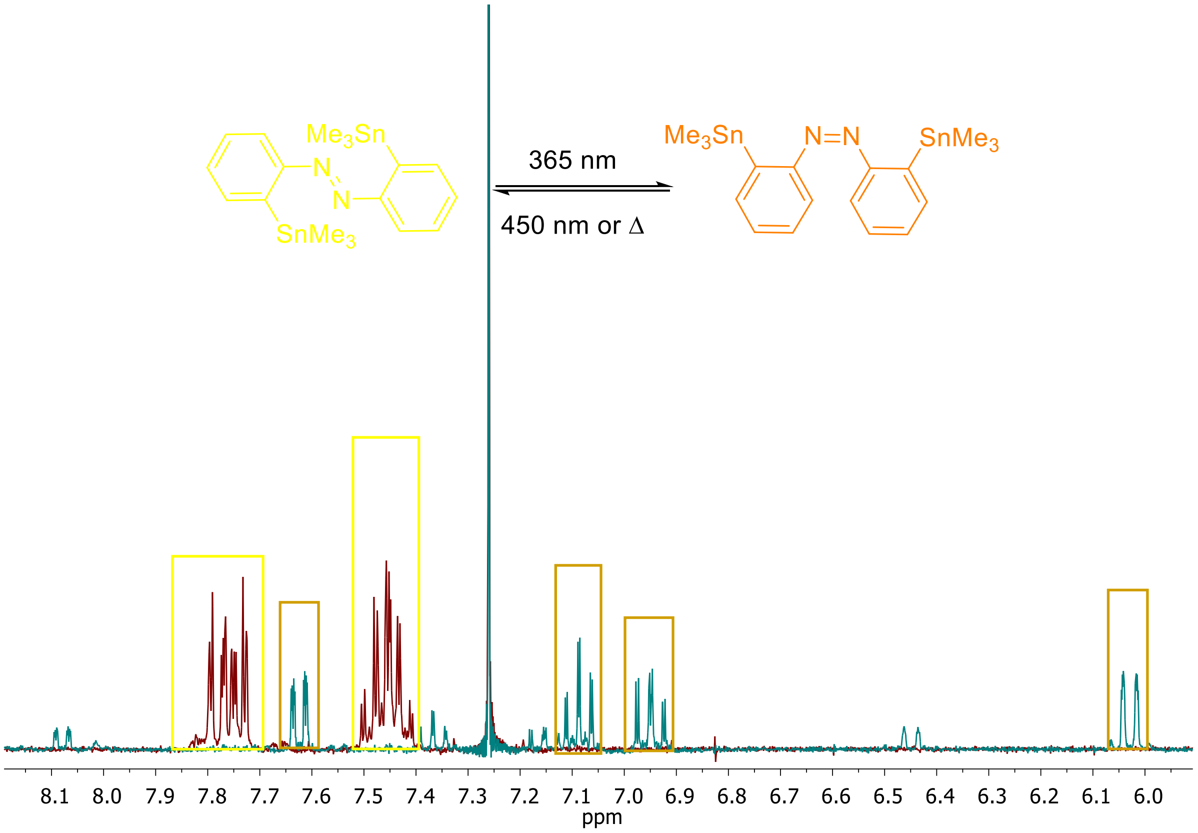

Figure SI36: ^{1}H NMR (300 MHz) spectra of compound **8** in $CDCl_3$ before (red) and after (blue) irradiation with 365 nm (15 min). The concentration was 6.56 x 10^{-3} M.

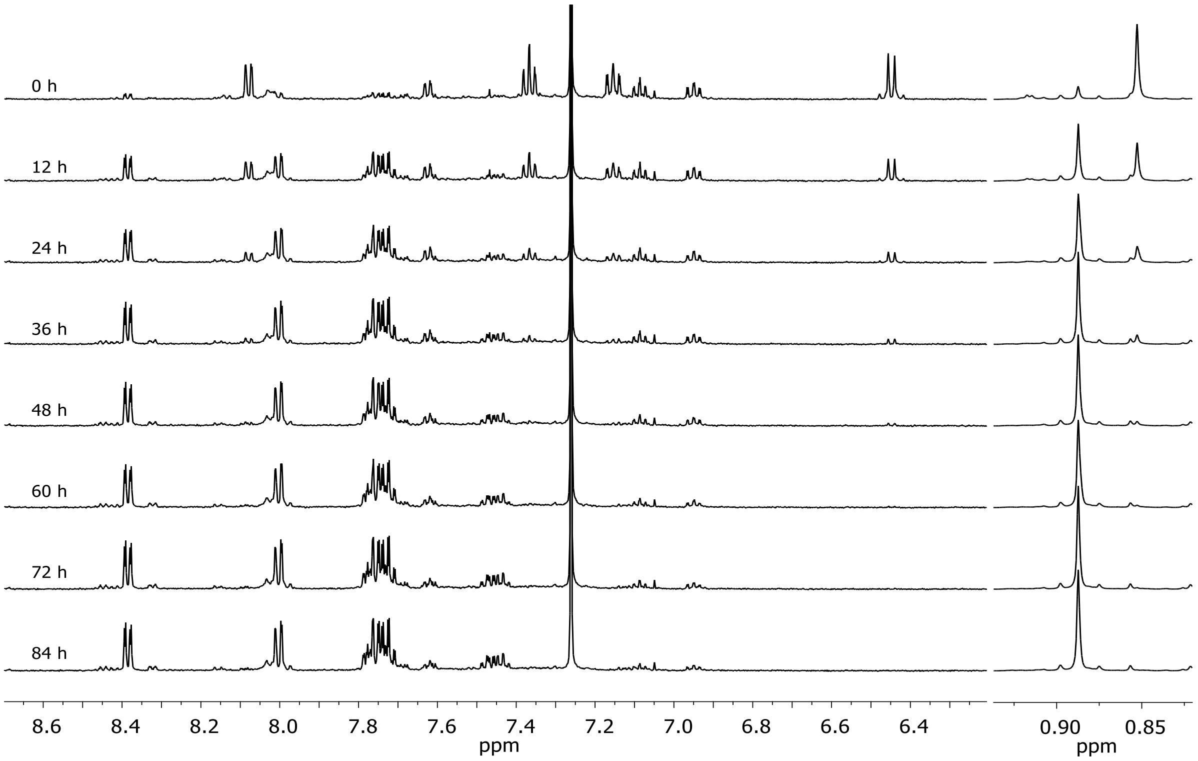

Figure SI37: ^{1}H NMR (500 MHz) spectra of **8** measured every 12 h after irradiation at 365 nm for 15 min.

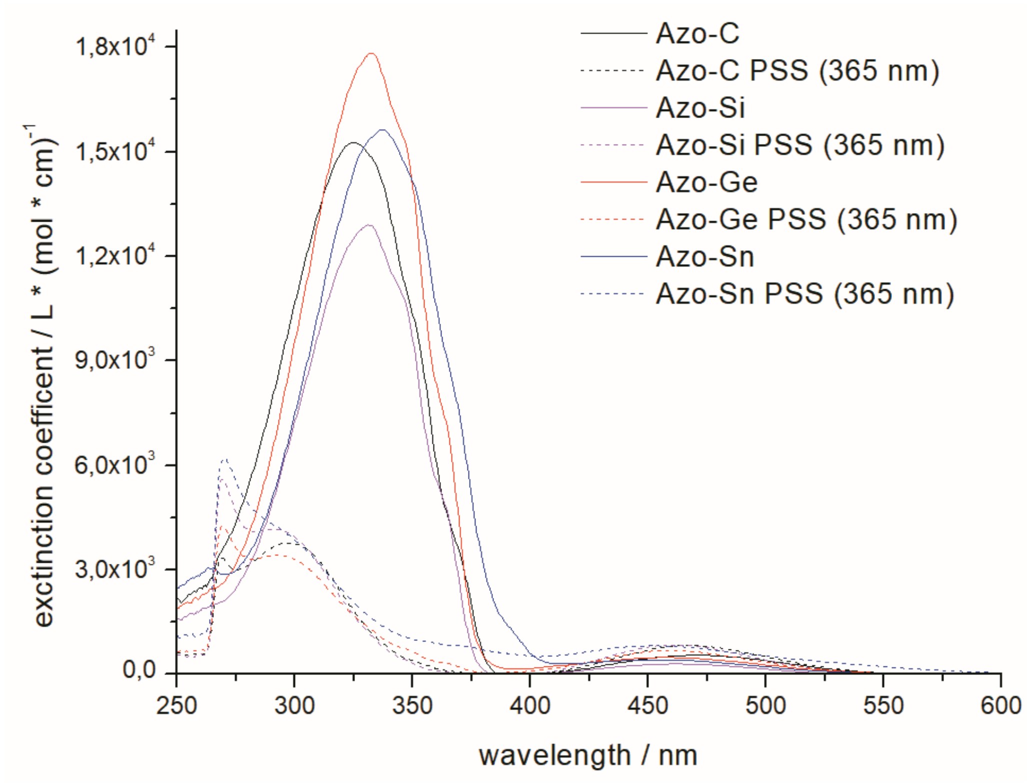

Figure SI38: Comparison of the switching behavior of all target molecules with their respective absorption spectra after irradiation at 365 nm for 3 min.

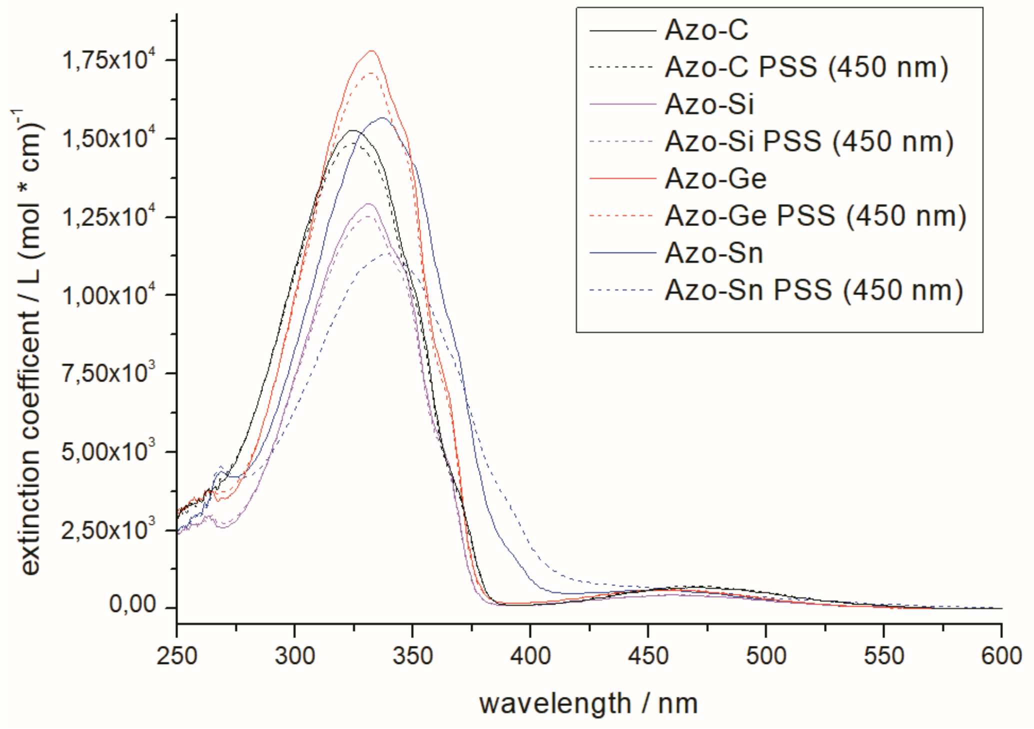

Figure SI39: Comparison of the switching behavior of all target molecules with their respective absorption spectra after irradiation at 450 nm for 15 min.

5. Thermoanalysis (DSC and TGA) and ^{1}H / $^{13}C\{^{1}H\}$ NMR Spectra of the Respective Compounds After Thermoanalysis

2,2'-Di(*tert*-butyl)azobenzene (**10**)

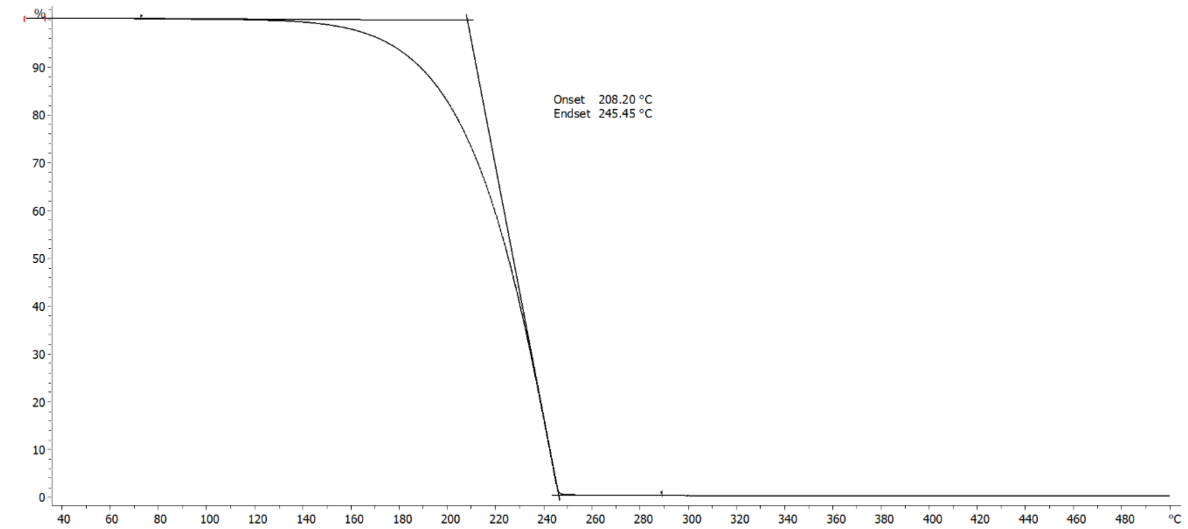

Figure SI40: Thermogravimetric analysis of compound **10** with a heating rate of 10 K / min under a nitrogen flow of 20 mL/min in a open aluminium crucible (40 µL).

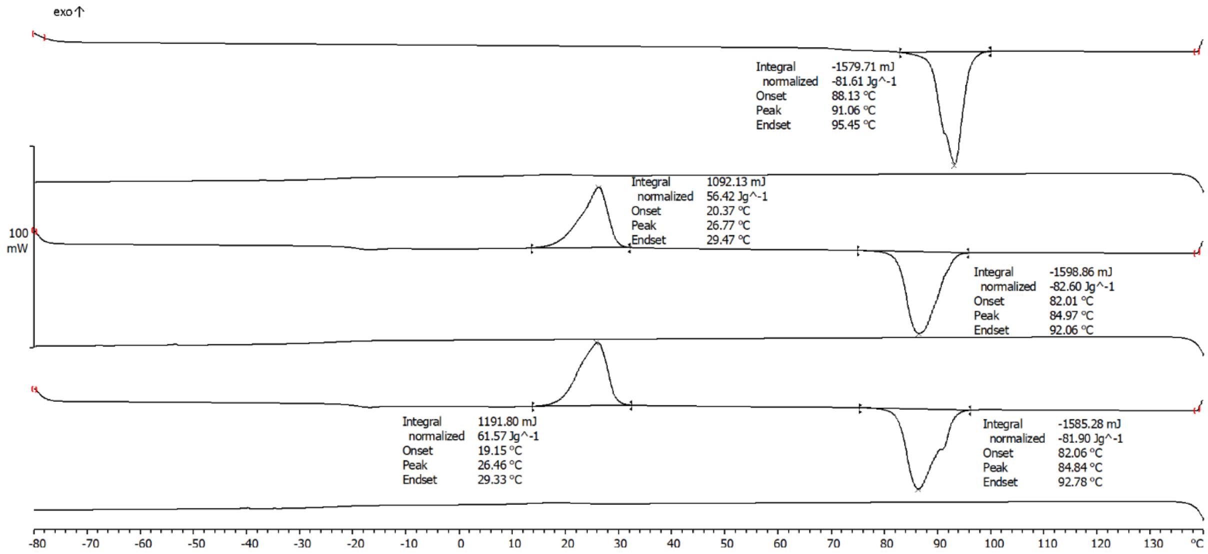

Figure SI41: Dynamic scanning calorimetry of compound **10** with a heating rate of 10 K / min under a flow of nitrogen (20 mL / min) in an aluminium crucible (40 µL) with a pierced lid.

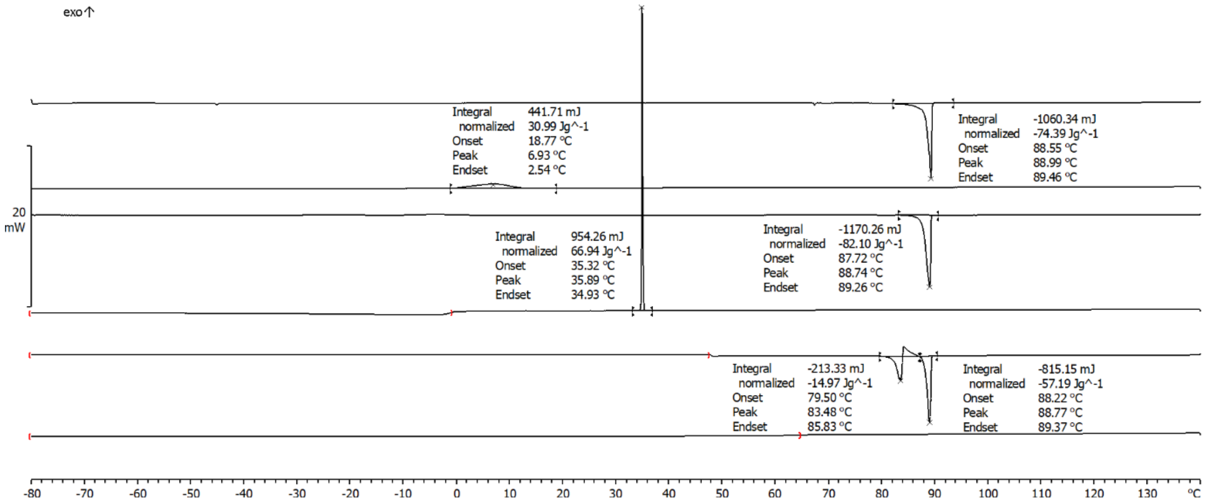

Figure SI42: Dynamic scanning calorimetry of compound **10** with a heating rate of 0.5 K / min under nitrogen flow (20 mL / min) in an aluminium crucible with a pierced lid (40 µL).

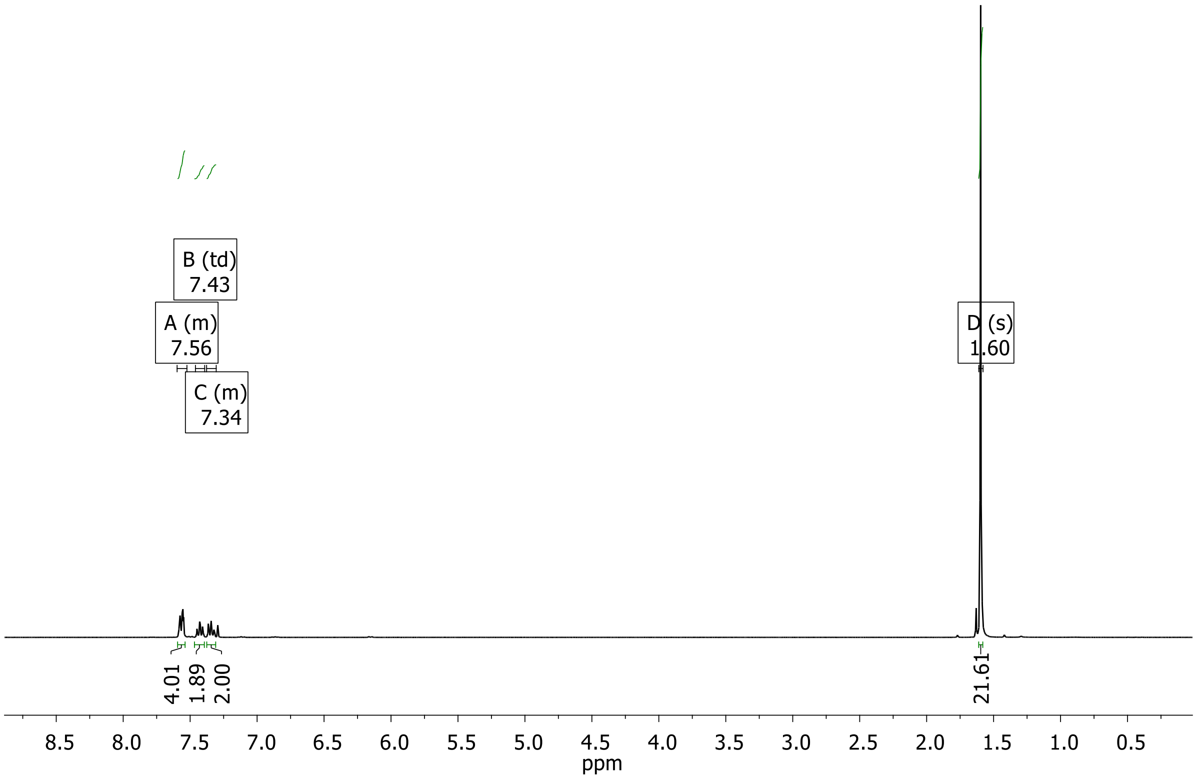

Figure SI43: ^{1}H NMR (380 MHz) spectrum of compound **10** after DSC.

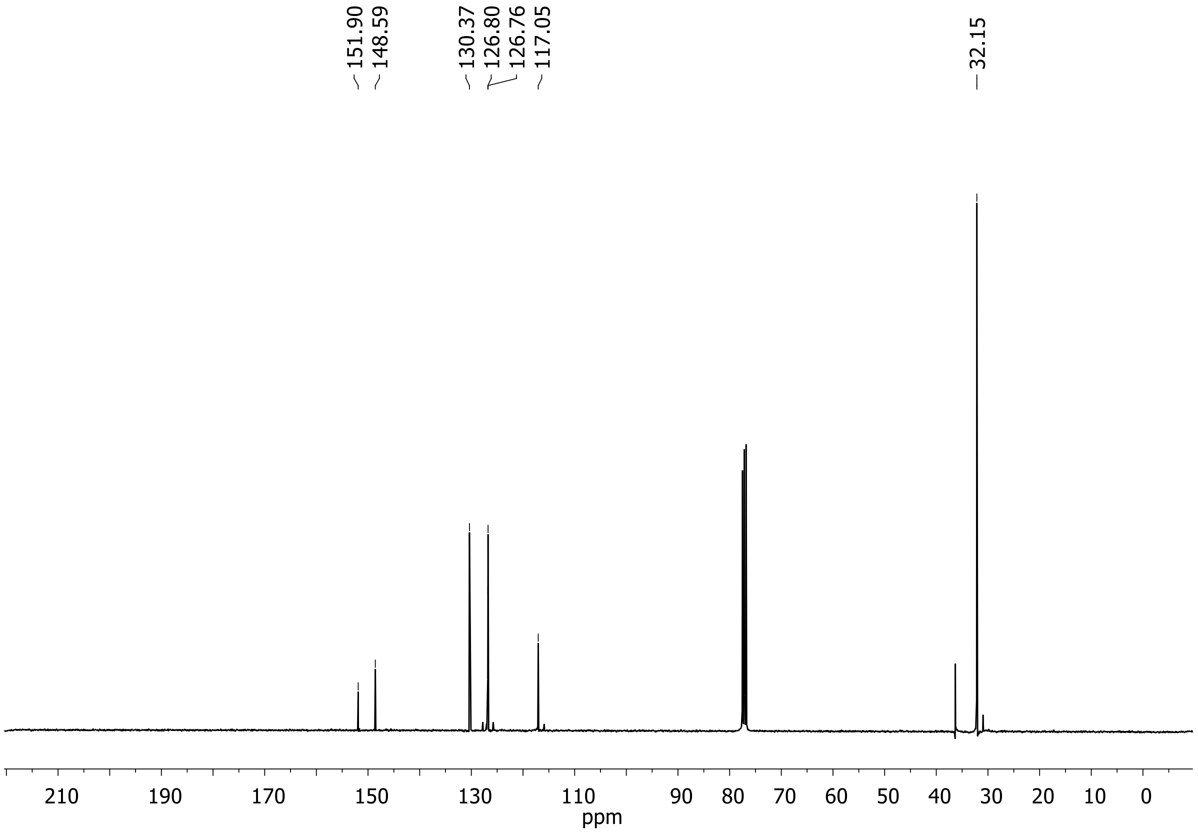

Figure SI44: $^{13}C\{^{1}H\}$ NMR (76 MHz) spectrum of compound **10** after DSC.

Due to the fact that the TG analyses showed a constant loss of mass at higher temperature we were interested in the nature of this process. Therefore we interrupted the measurement at 220 °C at measured ^{1}H and ^{13}C NMR spectra.

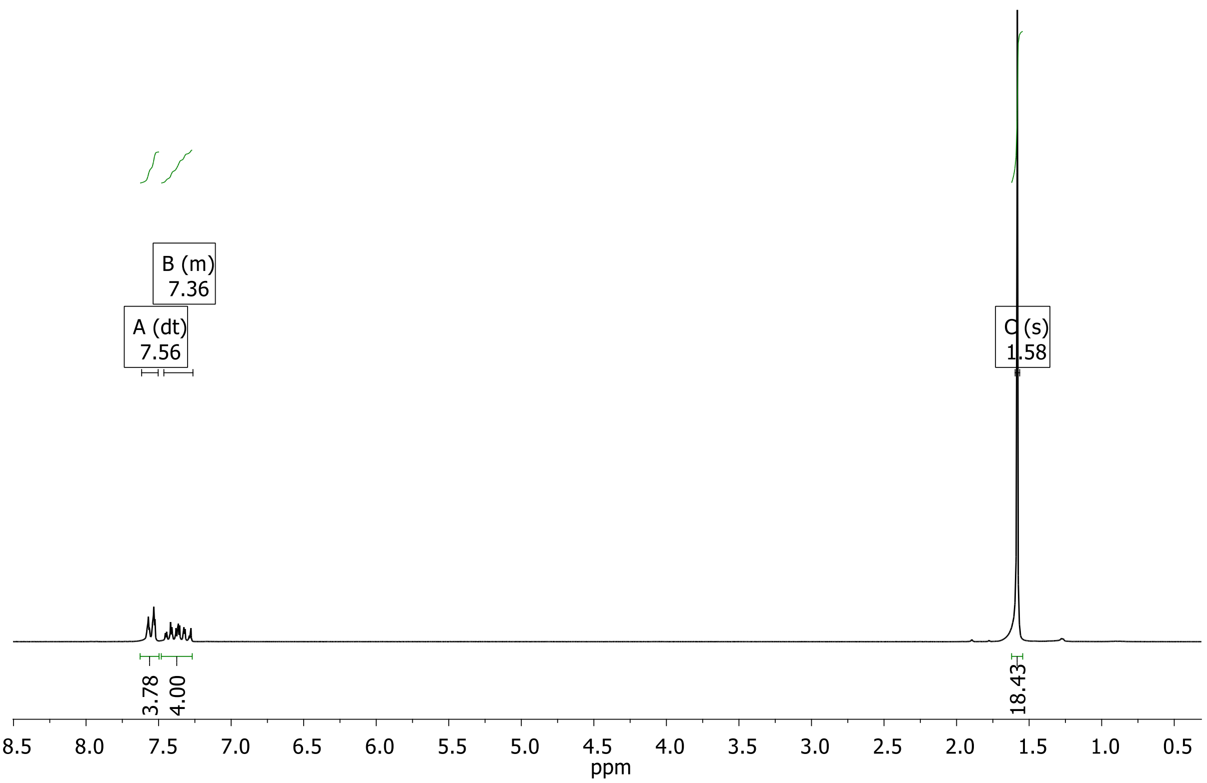

Figure SI45: ^{1}H NMR (380 MHz) spectrum of compound **10** after interrupting the TG analyses at 220°C.

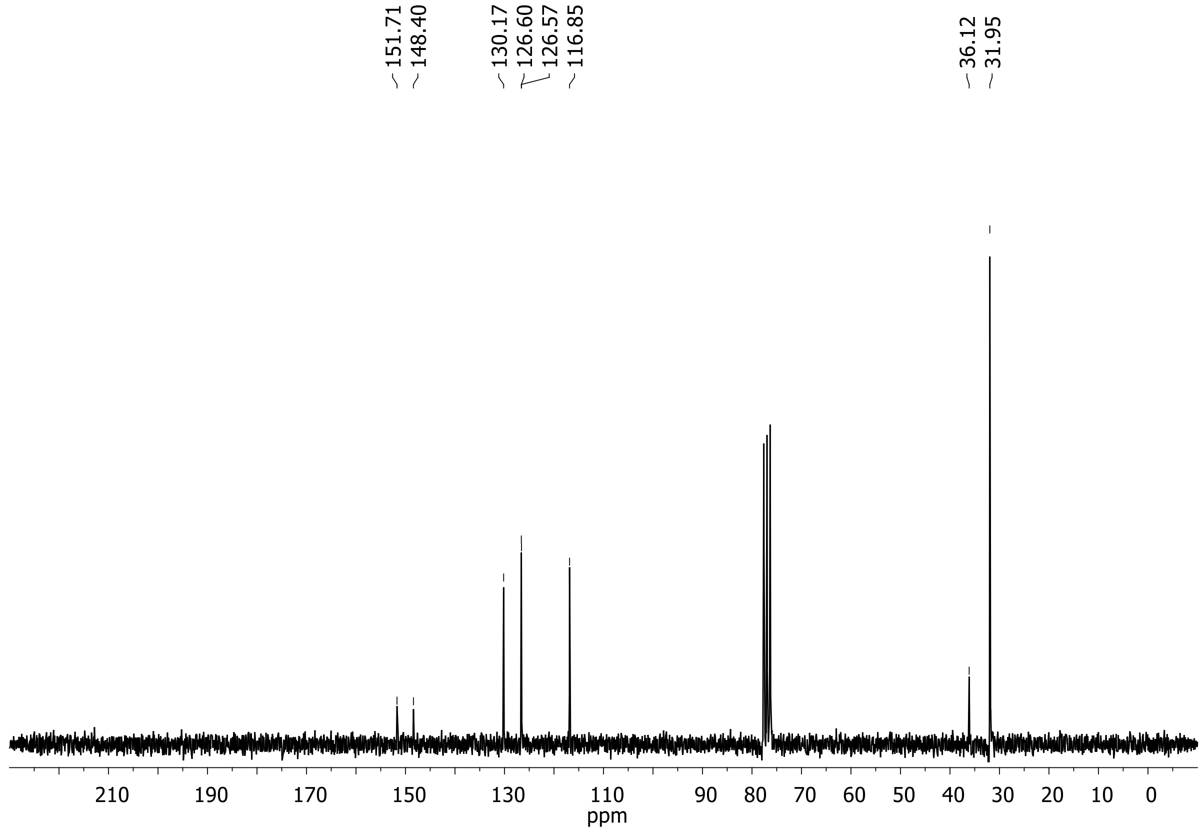

Figure SI46: $^{13}C\{^{1}H\}$ NMR (76 MHz) spectrum of compound **10** after interrupring the TG analyses at 220°C.

The obtained spectra are in agreement with the spectra of the purified materials indicating no decomposition but evaporation of the molten azobenzene.

2,2’-bis(Trimethylsilyl)azobenzene (**6**)

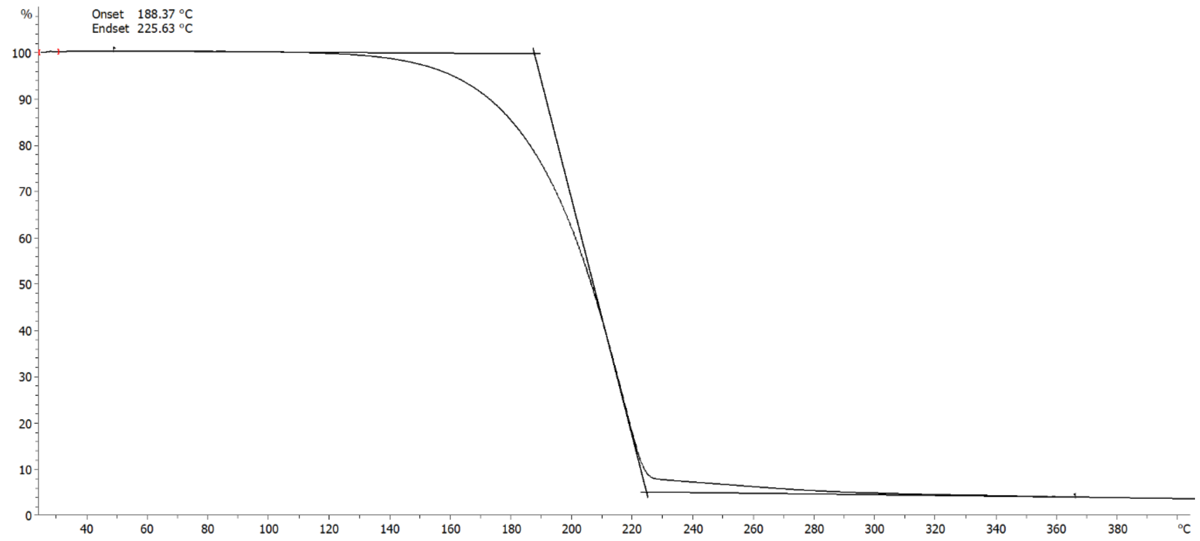

Figure SI47: Thermogravimetric analysis of compound **6** with a heating rate of 10 K / min under a nitrogen flow of 20 mL/min in a open aluminium crucible (40 µL).

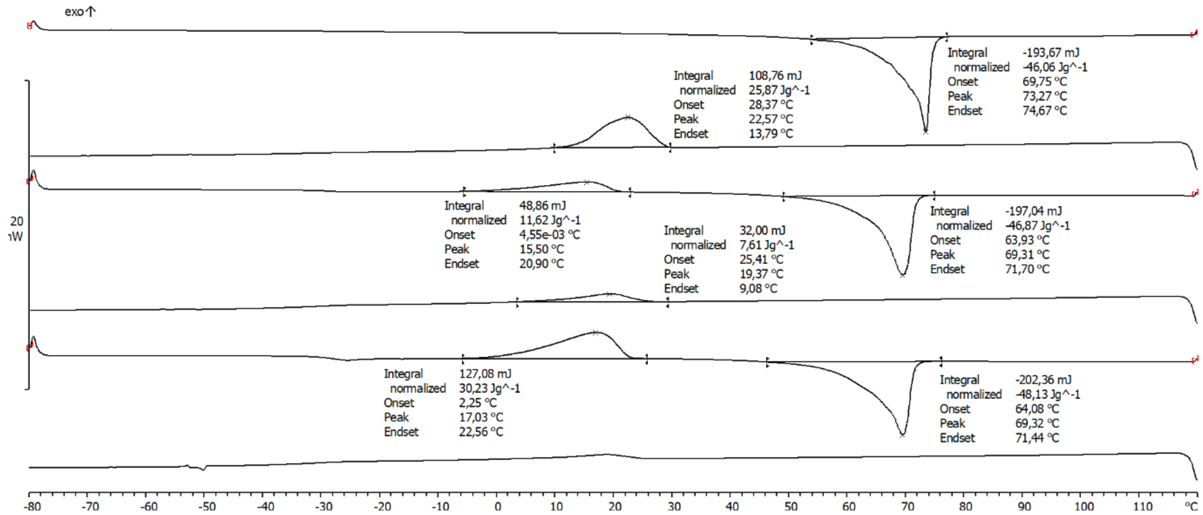

Figure SI48: Dynamic scanning calorimetry of compound **6** with a heating rate of 10 K / min under nitrogen flow (20 mL / min) in an aluminium crucible with a pierced lid (40 µL).

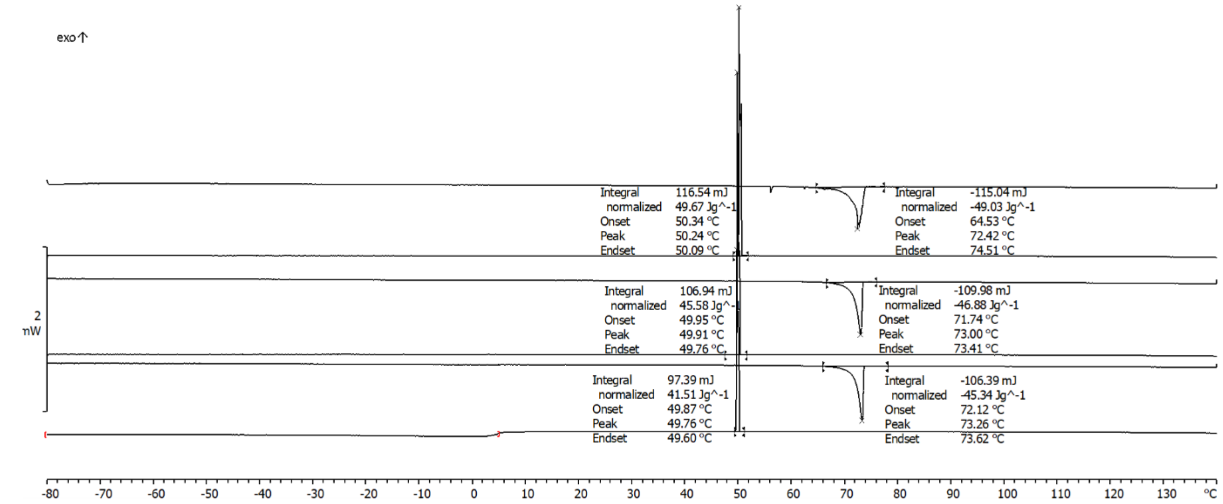

Figure SI49: Dynamic scanning calorimetry of compound **6** with a heating rate of 0.5 K / min under nitrogen flow (20 mL/min) in an aluminium crucible with a pierced lid (40 µL).

2,2‘-bis(Trimethylgermanyll)azobenzene (**7**)

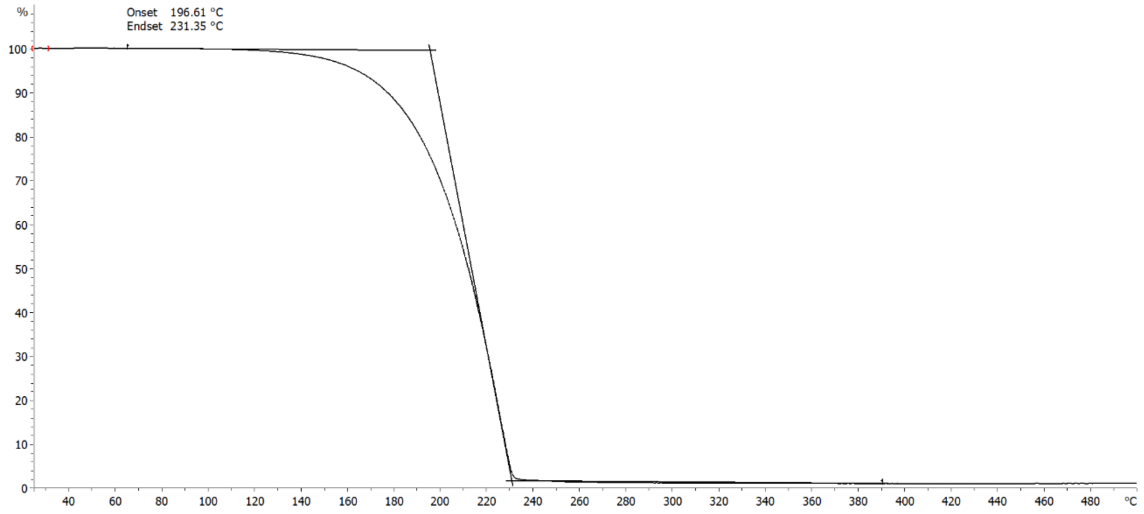

Figure SI50: Thermogravimetric analysis of compound **7** with 10 K / min under a nitrogen flow of 20 mL/min in a open aluminium crucible (40 µL).

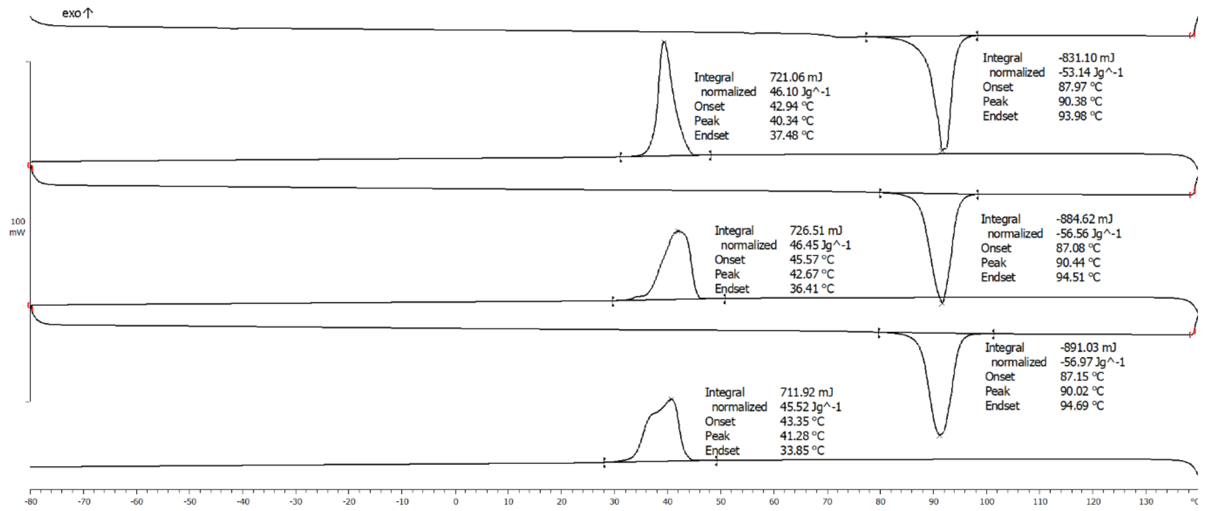

Figure SI51: Dynamic scanning calorimetry of compound **7** with a heating rate of 10 K / min under nitrogen flow (20 mL/min) in an aluminium crucible with a pierced lid (40 µL).

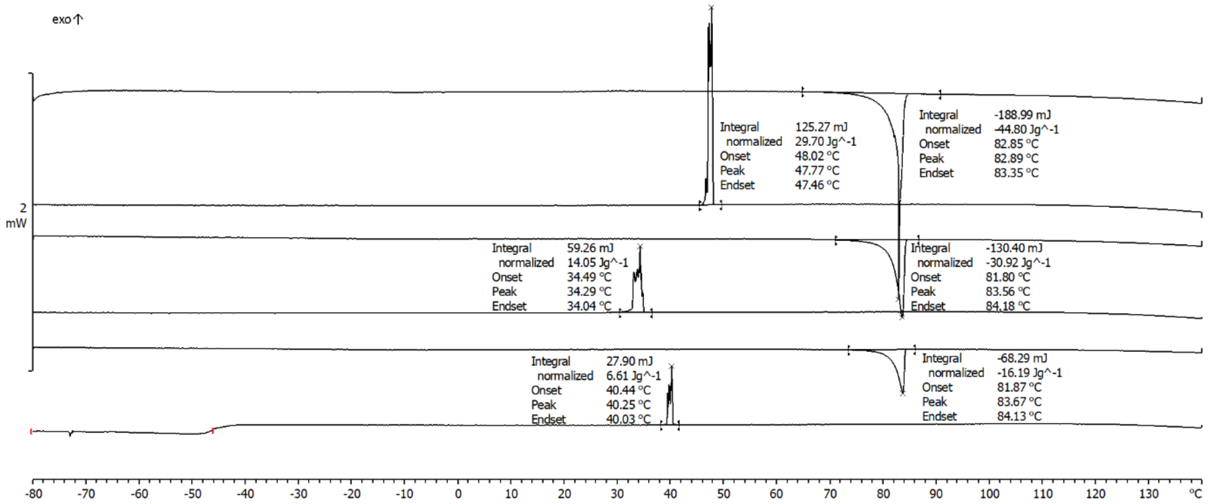

Figure SI52: Dynamic scanning calorimetry of compound **7** with a heating rate of 0.5 K / min under nitrogen flow (20 mL/min) in a closed aluminium crucible (40 µL).

2,2’-bis(Trimethylstannyl)azobenzene (**8**)

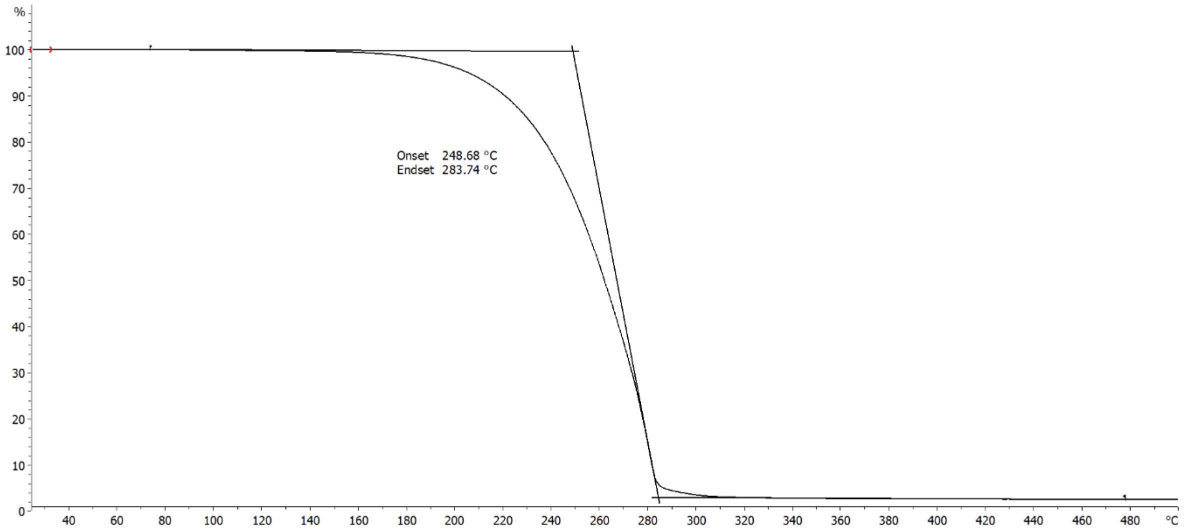

Figure SI53: Thermogravimetric analysis of compound **8** with a heating rate of 10 K / min under a nitrogen flow of 20 mL/min in a open aluminium crucible (40 µL).

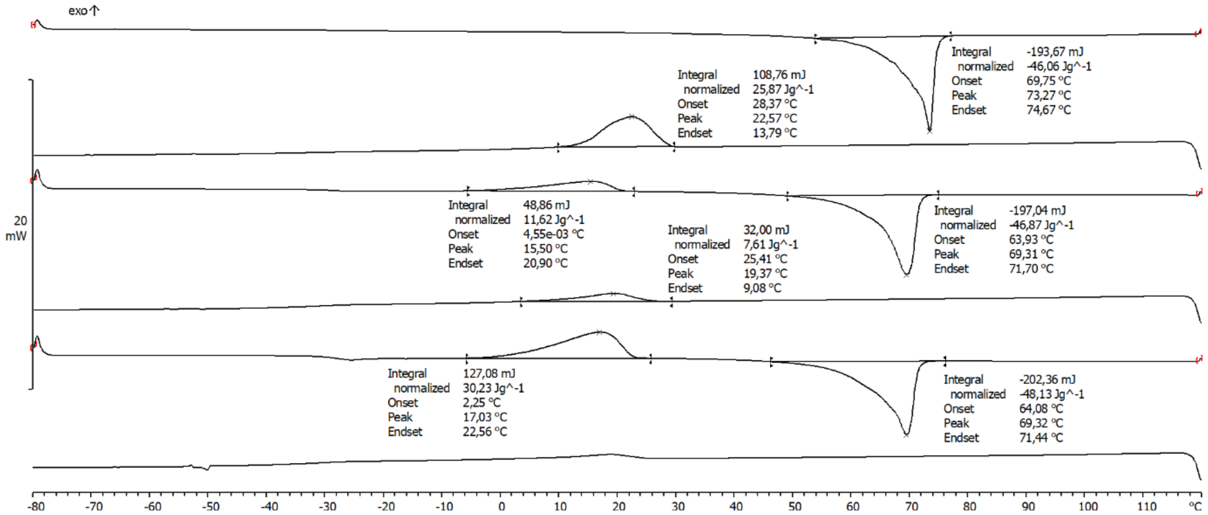

Figure SI54: Dynamic scanning calorimetry of compound **8** with a heating rate of 10 K / min under nitrogen flow (20 mL/min) in a closed aluminium crucible (40 µL).

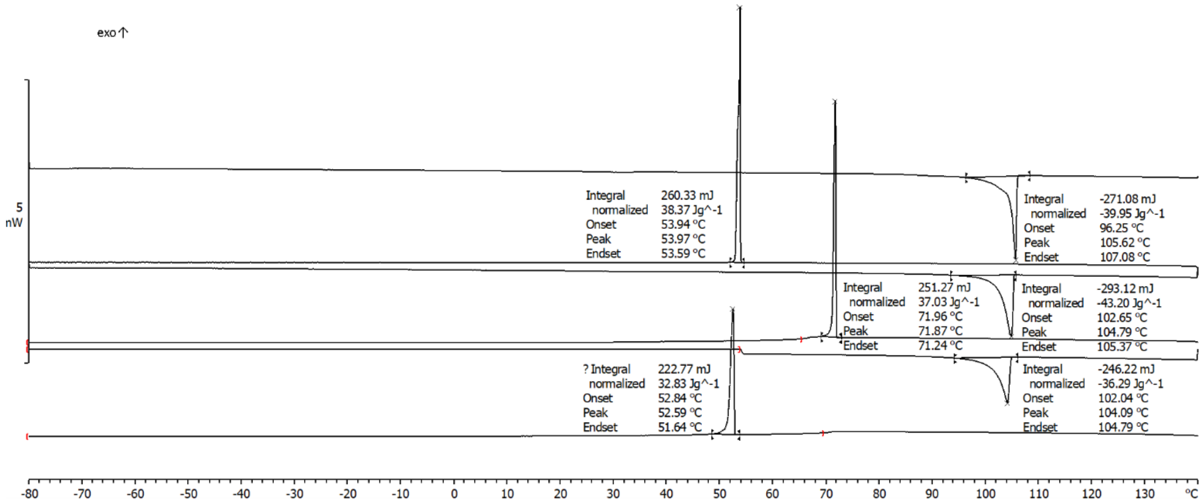

Figure SI55: Dynamic scanning calorimetry of compound **8** with a heating rate of 0.5 K / min under nitrogen flow (20 mL/min) in a closed aluminium crucible (40 µL).

1,6-Di(*tert*-butyl)phenanzine (**16**)

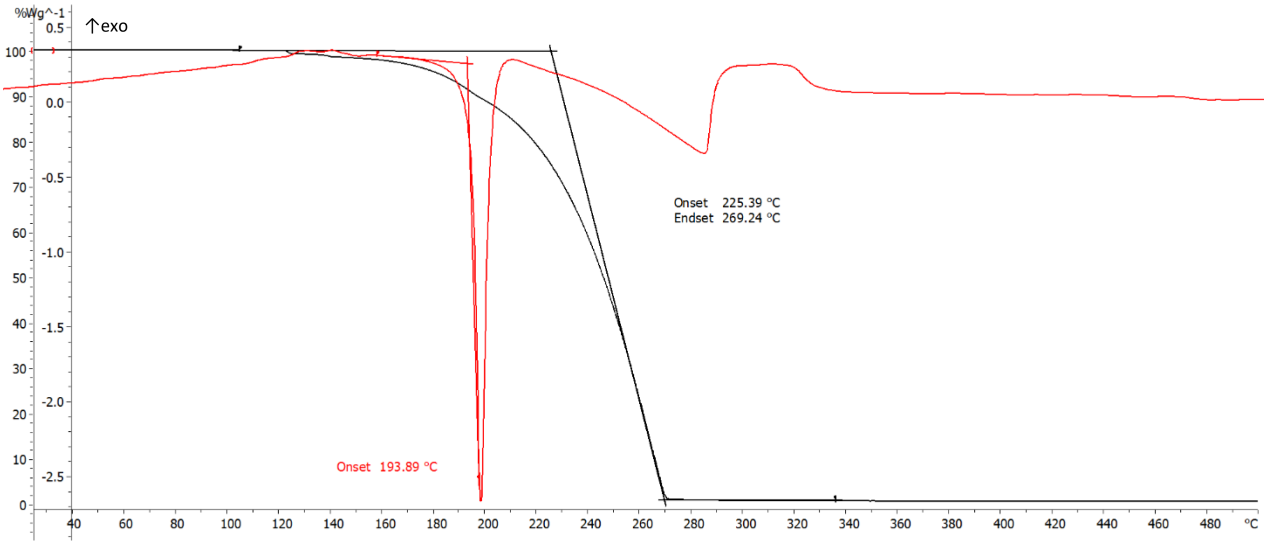

Figure SI56: Thermogravimetric analysis of compound **16** with a heating rate of 10 K / min under a nitrogen flow of 20 mL/min in a open aluminium crucible (40 µL). The red line indicates the DSC data.
